# Perhydrohelicenes and other diamond-lattice based hydrocarbons: the choreography of inversion†
†Electronic supplementary information (ESI) available. See DOI: 10.1039/c7sc01759f



**DOI:** 10.1039/c7sc01759f

**Published:** 2017-07-17

**Authors:** Roger W. Alder, Craig P. Butts, Richard B. Sessions

**Affiliations:** a School of Chemistry , University of Bristol , Bristol , BS8 1TS , UK . Email: rog.alder@bristol.ac.uk; b School of Biochemistry , University of Bristol , Bristol , BS8 1TD , UK

## Abstract

Overall inversion in fused cyclohexane oligomers **2**, **3**, and **4** (all based on *cis*-decalin **1**) occurs by a rolling process involving no more than two adjacent rings in twist-boat conformations at any time. These inverting rings move along the oligomer in processes that are precisely choreographed by the adjacent chairs. Actual inversion mechanisms can be stepwise [CC → TC → TT → C′T → C′C′], as for *cis*-decalin, but it is shown that a concerted alternative [CC → TC → C′T → C′C′] is enforced in **2**. The all-*cis*,*anti*,*cis*-isomers of perhydrohelicenes **4** are based on the diamond lattice and have remarkably low strain energies. Helix inversion in **4** is compared with that in helicenes **5**. For both, the intermediates and transition states have shapes broadly like kinked old-style telephone cables. In both cases barriers increase with the length of the system to eventually reach a plateau value of *ca.* 120 kJ mol^–1^ for **4**, much lower than that for **5** (320–350 kJ mol^–1^). While rolling inversion only requires two adjacent rings in twist-boat conformations at any instant, inversion in propellane **6** requires all three rings be converted to twist-boats, and the *S*_4_ symmetric hydrocarbon **7** requires all four rings to be converted to twist-boats. As a consequence, **7** probably has the highest barrier of any non-oligomeric *cis*-decalin derived structure (87.3 kJ mol^–1^ at B3LYP/6-31G*).

## Abstract 2

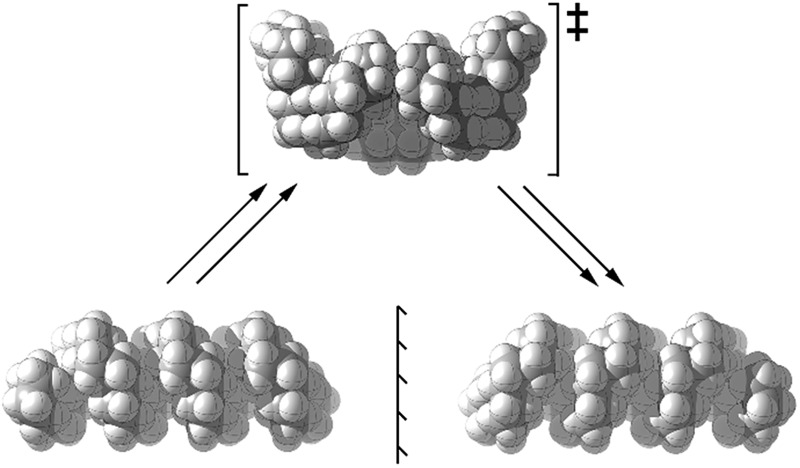
Overall inversion in fused cyclohexane oligomers **2**, **3**, and **4** (all based on *cis*-decalin **1**) occurs by a rolling process involving no more than two adjacent rings in twist-boat conformations at any time.

## Introduction

This paper examines the structure, strain, and conformational behaviour of some hydrocarbons based on *cis*-decalin **1**. We look at oligomers **2**, **3**, and **4** (the latter are saturated analogues of the well-known helicenes **5**), propellane **6**, and tetracycle **7**.
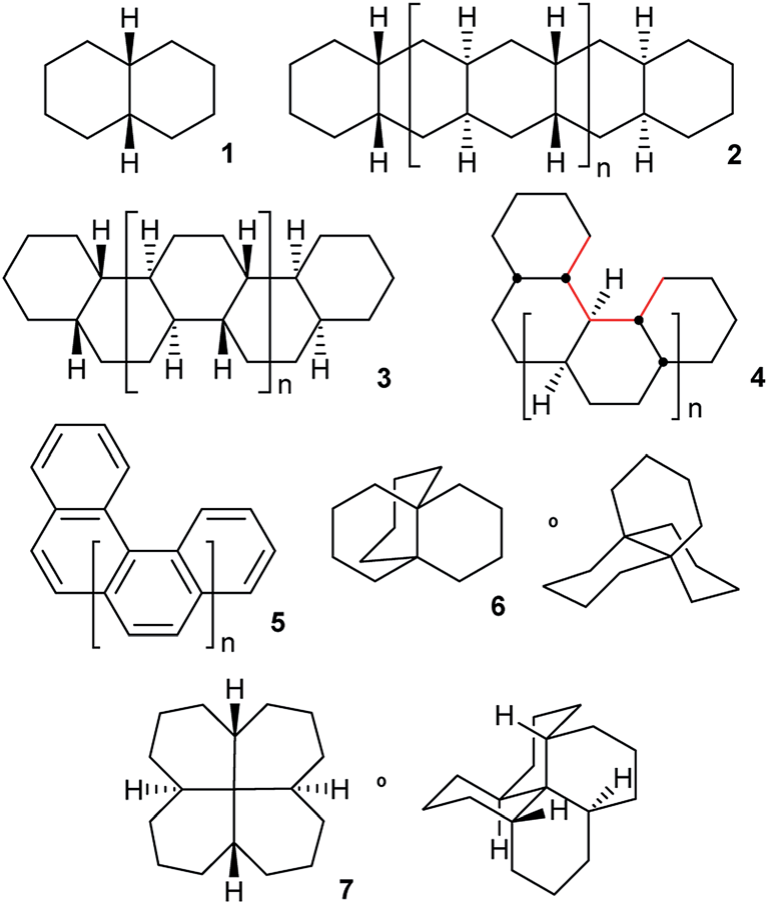


**Fig. 1:**
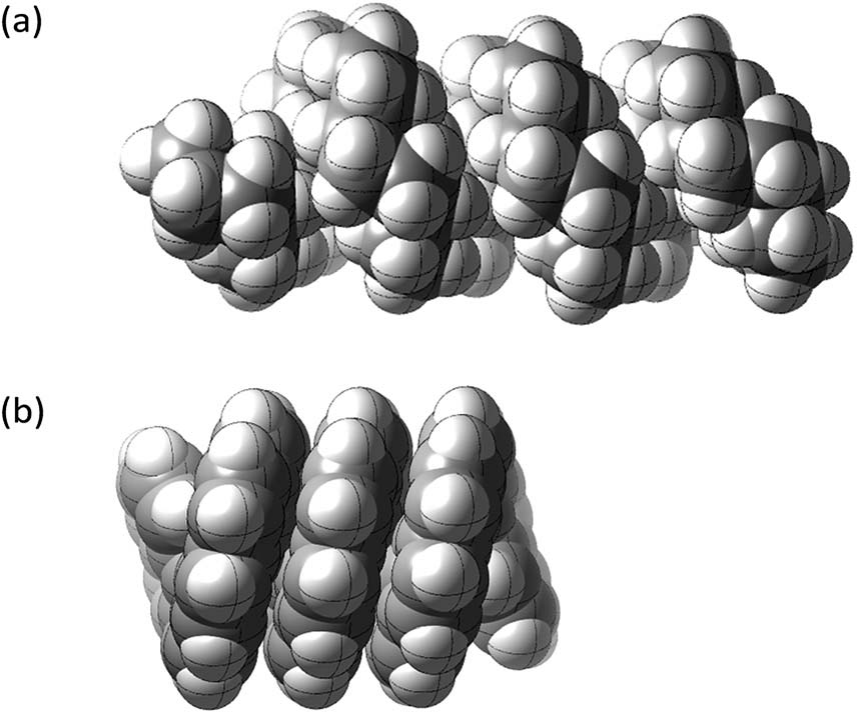
Ground state of (a) all-*cis*,*anti*,*cis*-isomer of perhydro[20]helicene; (b) [20]helicene.

Structures **2**, **3**, **4**, **6**, and **7** are based on the diamond lattice but also lack any g^+^g^–^ (*syn*-pentane) interactions in their ground states. Like *cis*-decalin itself, all the are capable of ring inversion and we examine how this can result in inversion of the whole structure. The all-*cis*,*anti*,*cis*-isomer **4** (perhydrohelicene) is particularly interesting. Its structure is of course a helix – see Fig. 1(a) for a picture of the [20]mer, but it is a much more open one than the [20]mer of helicene **5** shown in Fig. 1(b), and we show that **4** has remarkably little strain in its all-chair conformation.

The unique stability of the chair form results in a high barrier to ring inversion in cyclohexane.^1^ What is the barrier for overall inversion of polycyclic structures like **2**–**7**? We hoped to find if there is an upper limit for inversion barriers in structures of this type. One important consideration is likely to be the number of rings that must be in non-chair conformations (twist-boat or simply twist) simultaneously to achieve full inversion? We show that in oligomers **2**, **3**, and **4**, a sequential process that we call rolling inversion works: CCCC… → TCCC… → TTCC… → C′TCC… →→ C′TTC… *etc.* (T represents a twist conformation and C′ represents an inverted chair). With ≤2 twist rings required at any instant, the barrier can be quite low, although those twist rings must undergo a random walk to eventually reach the far end of the oligomer. We show later that rolling inversion fails for **6** and **7**. Inversion of **6** requires 3 rings to be in twist form simultaneously and **7** requires all four, so *S*_4_-symmetric **7** may have the highest barrier for any non-oligomeric *cis*-decalin derived structure: 87.3 kJ mol^–1^ at B3LYP/6-31G*.

One unexpected conclusion of our studies is that overall inversions are precisely choreographed by the structure and position of the chair rings adjacent to the twist rings in the rolling inversion. We expect this choreography to apply to a wider range of structures beyond those covered in this paper. We spell out this choreography in detail for **3** and **4** but each step of the rolling inversion involves the stepwise [CC → TC → TT → C′T → C′C′] mechanism established in the seminal study of *cis*-decalin **1** by Baas *et al.* ^2^ For the interior of linear oligomer **2** however, we find that the stepwise mechanism fails, and a novel pathway [CC → TC → C′T → C′C′] having a concerted TC → C′T step is required.

Finally, we will show that helix inversion in **4** involves the same type of non-bonded interactions as in **5**, but that these are far less severe, and we estimate that the limiting barrier for inversion in **4** may be only ∼120 kJ mol^–1^ compared with 320–350 kJ mol^–1^ for **5**.

## Computational methods

DFT calculations were performed using Gaussian 09.^3^ Conformational searches were carried out by Monte Carlo methods using Spartan.^4^ Illustrations were prepared using CYLview.^5^ An economical DFT method and limited basis set were required since structures as large as perhydro[12]helicene (C_50_H_78_) were studied. We have therefore limited geometry optimizations to the B3LYP/6-31G* level. It seemed possible however that differential intramolecular dispersion interactions could be significant, so single point B3LYP-gCP-D3/6-31G* energies^6^ were obtained using the web service: ; http://www.thch.uni-bonn.de/tc/gcpd3. In the text B3LYP-gCP-D3/6-31G*//B3LYP/6-31G* energies are quoted with B3LYP/6-31G* values in parentheses; both include zero point energies. In practice, inclusion of gCP-D3 corrections only leads to small differences (<9 kJ mol^–1^ and typically <4 kJ mol^–1^) except in the case of aromatic helicenes **5**, where activation energies are increased by 30–40 kJ mol^–1^. NMR chemical shifts were estimated by single point B3LYP/6-311+G(2d,p) calculations in Gaussian and approximate NMR coalescence temperatures were estimated based on these calculated shifts combined with the expected energy barriers for conformational exchange.

The energies and *xyz* coordinates for all the ground states, intermediates and TSs discussed in this paper are listed in the ESI.†

## Results and discussion

Whether inversion can occur in multi-ring systems depends on how the rings are linked. Fused-ring systems like *trans*-decalin **8** cannot undergo ring inversion to the alternative chair form and are usually described as conformationally locked, although individual rings can be converted to twist conformations so this is something of a misnomer. In oligomer **9**, the internal rings have no conformational freedom at all: boat or twist conformations are unattainable. Rings of this type will be described as rigid in this paper. *cis*-Decalin **1** is more flexible than **8**, and ring inversion results in an enantiomerization process with a barrier that is significantly higher than for cyclohexane itself because both rings must be converted to twist conformations to achieve inversion. As described by Baas *et al.* ^2^ the rate-limiting transition state (TS) involves the conversion of the last chair to a twist. Passage through a conformation with a plane of symmetry (BB) occurs as a pseudorotation (PR) of the twist, twist (TT) form and involves a lower barrier. This is the stepwise mechanism mentioned above [CC → TC → TT → C′T → C′C′].

This paper is only concerned with fused systems related to **1**, but it is worth noting that spirans like spiro[5.5]undecane **10** can always undergo ring inversion, whereas bridged-ring compounds like bicyclo[3.3.1]nonane **11** can never invert, although both rings in **11** can again be converted to twist conformations – they are also conformationally locked.
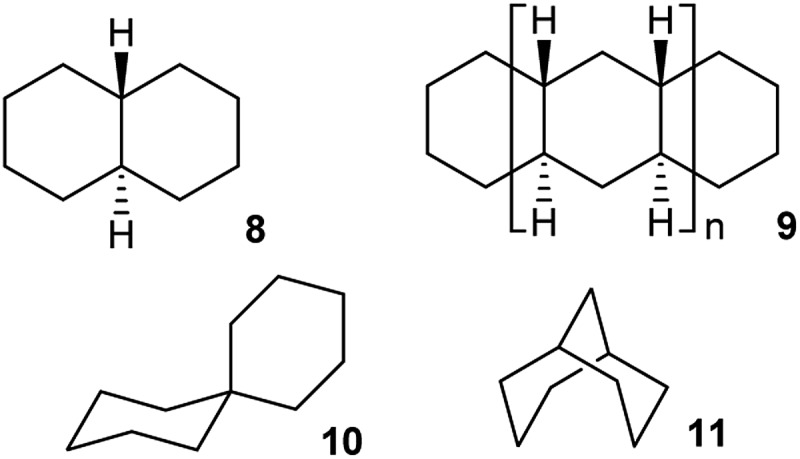


Oligomers **2**, **3**, and **4** are all-*cis*,*anti*,*cis*-isomers and adopt ground-state all-chair conformations so the carbon skeletons align with the diamond lattice and therefore have ideal bond angle and staggered conformations. However, low strain energies are only guaranteed if g^+^g^–^ (*syn*-pentane) and longer range non-bonded interactions are also absent. Isomers with *syn* ring junctions will have g^+^g^–^ (*syn*-pentane) interactions, will be strained and will not be discussed here. This restriction on our discussion is largely for economy, but also because the raised energies of the lowest energy conformations of these isomers (which might indeed not be all-chair) means that adding rings of this type may not raise inversion barriers much. To check on this we did examine one example: (4*aR*,8*aR*,9*aS*,10*aS*)-dodecahydro-4*a*,9*a*-butanoanthracene, shown below with the *syn*-pentane moiety picked out in red. The barrier for conversion of the last chair to a twist in this example is 72.1 (71.1) kJ mol^–1^ compared with 71.0 (69.8) kJ mol^–1^ for **6** itself.
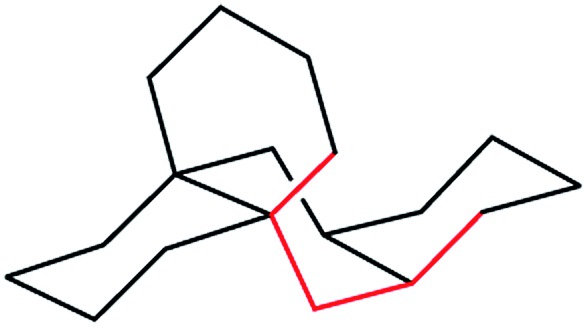


Detailed discussion in this paper will be confined to the parent hydrocarbons, but most of our conclusions should apply to substituted compounds and particularly to hetero-analogues that may be of much more practical interest. Some of these possibilities will be highlighted later.

### Pseudorotation of twist rings flanked by chairs

Fusion of one or more chair rings to a twist ring imposes strong limits on pseudorotation (PR) in that ring. Baas *et al.* ^2^ showed that for CT *cis*-decalin, PR is limited to conversion of what they term CT_–60_ to CT_+60_ *via* CT_0_ (a diagram similar to Fig. 9 in their paper is included in the ESI† for convenience).
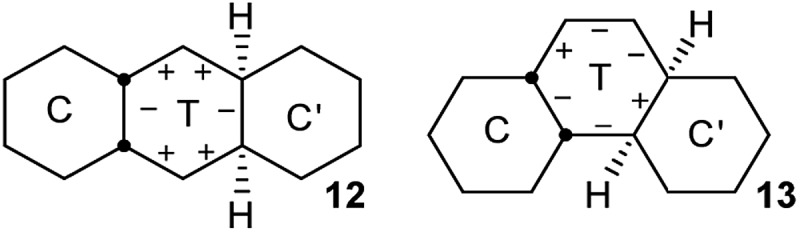


In *cis*,*anti*,*cis*-perhydroanthracene^7–9^ and *cis*,*anti*,*cis*-perhydrophenanthrene,^10^ the fusion of two chair rings effectively kills PR in the CTC′ conformations **12** and **13** shown above. These represent the core structures of intermediates with one twist ring in the inversion of **2**, **3**, and **4**. The central twist rings in **12** and **13** can pseudorotate only as far as the adjacent boat structures in the PR itinerary but cannot reach the next twist form.

The TT intermediates in the inversion of **3** and **4** are of course more flexible than the C′TC intermediates above, but even here PR is controlled by the adjacent chair rings. This leads to a strict sequence of steps for the overall inversion, and this choreography of the overall inversion is perhaps the most surprising conclusion of our study.

We show first that TT intermediates are actually impossible in the interior of **2** and that this leads to the concerted process described next.

### A concerted inversion mechanism

**Fig. 2:**
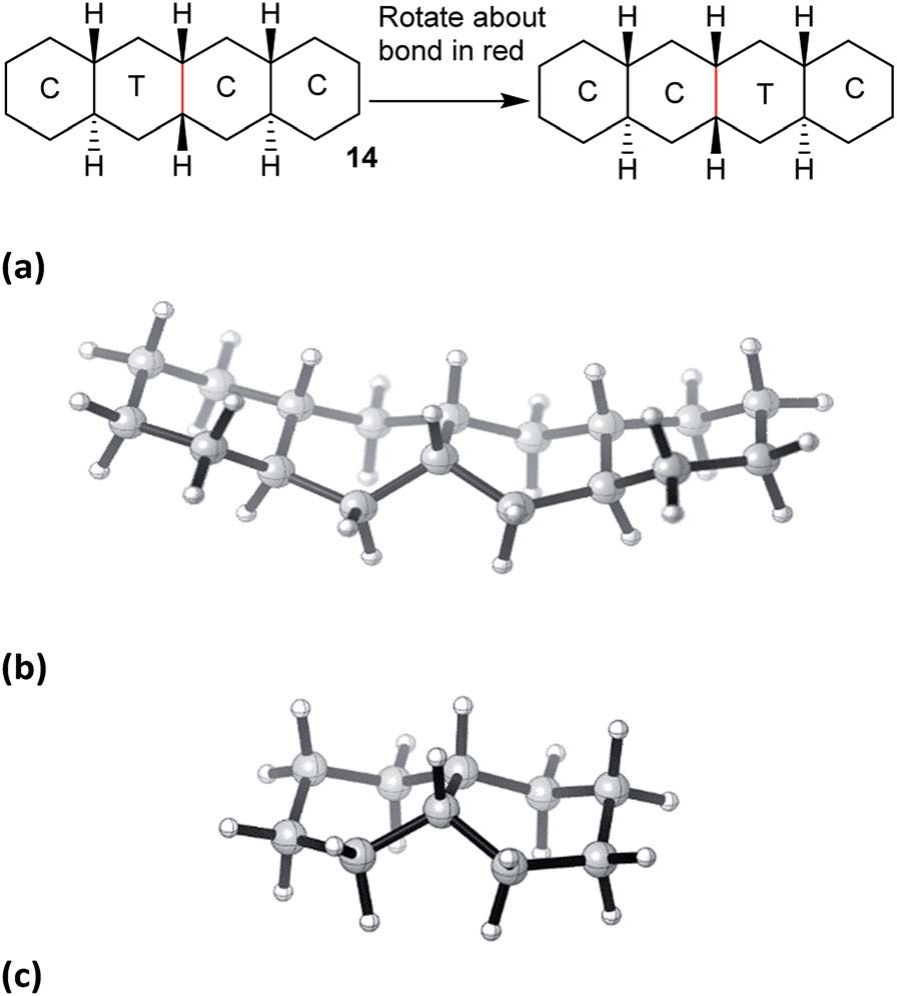
(a) The concerted mechanism of ring inversion of hydrocarbon **14**, (b) transition state for this inversion, not quite *C*_s_ symmetric, (c) core structure for the concerted inversion process.

The concerted mechanism is best introduced by considering a case where it is plain that no alternative is possible: *trans*,*cis*,*trans*-perhydrotetracene **14**. This unknown hydrocarbon^11^ cannot exist in an all chair form, and the CTCC conformation shown in Fig. 2(a) is the global minimum. More surprisingly, a conformation in which both inner rings are in twist form cannot be constructed; one must be chair and the other twist, even if the outer rings are also in twist form. Enantiomerization of **14** by the stepwise mechanism is therefore not possible.

Examination of physical models suggests that internal rotation about the C–C bond shown in red in Fig. 2(a) is the only possible conformational process in the two central rings. This exchanges the chair and twist inner rings and leads to enantiomerization.

Computational exploration of this concerted mechanism shows that such a process can indeed occur. The activation energy for **14** is lower than for *cis*-decalin **1**: 57.4 (54.5) kJ mol^–1^. This is not surprising, since **14** already contains one twist ring. In this and later cases, we have not attempted to predict coalescence temperatures which, in any case, depend on operating frequencies. Instead we will suggest broad temperature ranges where slow and fast exchange spectra are likely to be observed. In the case of **14**, calculated rate constants suggest that the ^13^C NMR spectrum would be in slow exchange at –75 °C (18 signals), but in fast exchange at ambient temperature (10 signals).

Baas *et al.* ^2^ do not discuss this concerted process for *cis*-decalin itself, but say “The high energy CC to TT transition state with a one-step inversion along the central bond is saved from uninterest not only because of its aesthetic attractiveness but also because it has two imaginary frequencies. This shows that it is a two-dimensional saddle point (a transition state between transition states)”. All our attempts to find a TS for a concerted inversion process for *cis*-decalin itself failed. Stripping the outer rings from the structure shown in Fig. 2(b) leads to the core structure shown in Fig. 2(c). This is not a stationary point on the *cis*-decalin surface; re-optimizing it as a TS actually leads to the lowest energy TS for CT to TT *cis*-decalin already reported as the rate-limiting TS by Baas *et al.* Alternatively, optimizing the core under imposed *C*_s_ symmetry led to a *C*_2v_ TS boat, boat structure that is on the pseudo-rotation pathway for TT *cis*-decalin but does not result in inversion.

All attempts to find a concerted TS for *cis*,*anti*,*cis*-perhydroanthracene^5,6^ **12** also failed and it can be concluded that the rigidity provided by both the outer chair rings in the structure shown in Fig. 2(b) is required for this structure to possess only one imaginary frequency and so sustain the concerted mechanism.
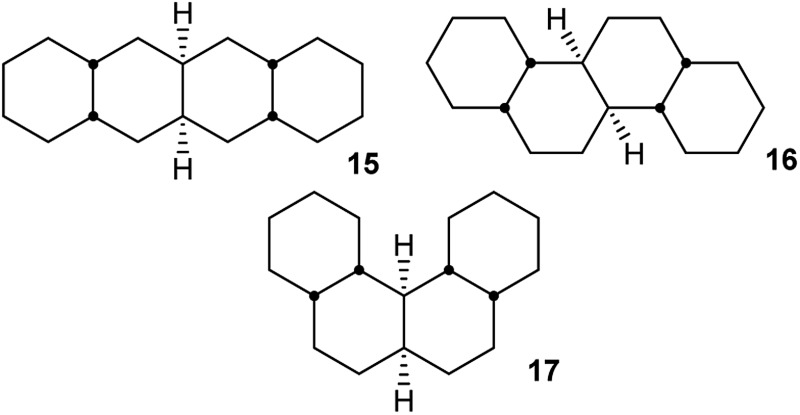


In hydrocarbon **14**, the outer rings are *trans*-fused, and the concerted mechanism might be just an oddity if it was confined to cases like this. However, it will now be shown that *cis*-fusion of pairs of chair rings to the core structure in Fig. 2(c) also supplies the required rigidity to lead to TSs for concerted mechanisms for inversion in the inner rings of some of the possible *cis*,*anti*,*cis*,*anti*,*cis* tetracyclic structures: **15**, **16**, and **17**. We will show that the concerted mechanism is enforced for **15** (and thus for all the inner rings in oligomer **2**), but the stepwise mechanism is more favourable in most other cases. Later we shall see that the concerted mechanism fails for **7** because it is only possible in this instance to fuse two true boat (not chair or twist) rings to the core structure discussed above, and these do not supply the required rigidity.

### *cis*,*anti*,*cis*,*anti*,*cis*-Perhydrotetracene **15** and ladder oligomer **2**

**Fig. 3:**
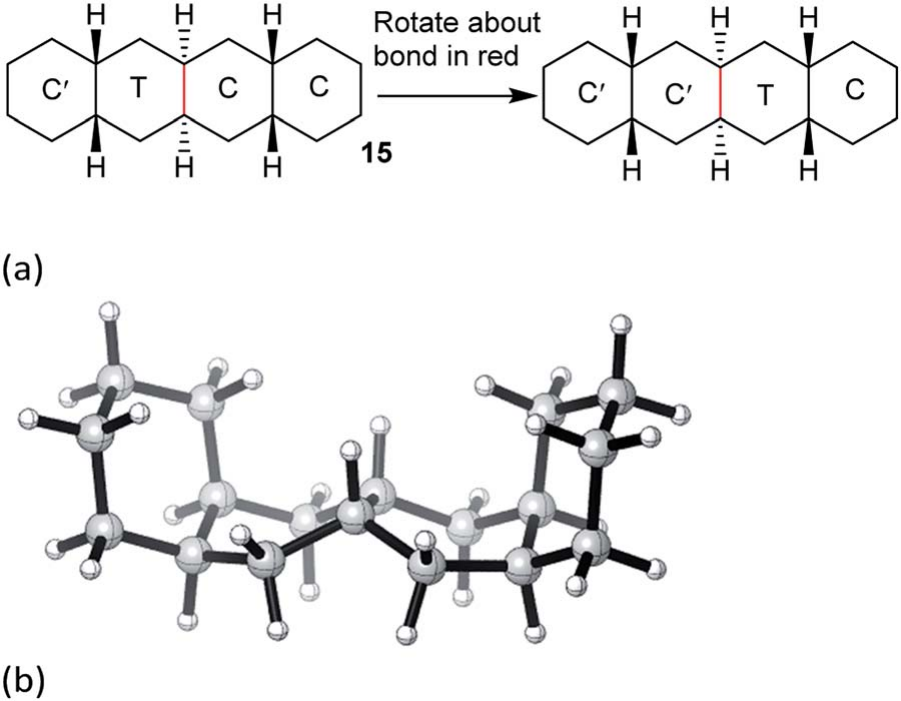
(a) Concerted inversion process for **15**, (b) transition state with *C*_s_ symmetry.

The concerted mechanism works for **15** as shown in Fig. 3. The TS lies 71.6 (69.4) kJ mol^–1^ above the all-chair ground state and 45.5 (43.2) kJ mol^–1^ above the C′TCC conformer. The TS shown in Fig. 3(b) has *C*_s_ symmetry so **15** is enantiomerized by this process; the rings to the left of the twist ring are inverted relative to those to the right. As in the case of **14** above, it is not possible to have both the inner rings simultaneously in twist conformations while the outer rings are C′ and C, so the concerted mechanism is enforced for the inner rings of **15**.

However, the concerted mechanism only works for the inner rings of **15**, because of the requirement for chair rings on either side. This means that there must be an alternative route from the CCCC global minimum to the first intermediate in Fig. 3(a). Moreover, the C′TCC conformer cannot be reached directly from the global minimum, so the lowest energy process goes from CCCC to TCCC, then to TTCC, before conversion to C′TCC. The rate-limiting TS for this sequence occurs for this last step and lies 66.9 (64.9) kJ mol^–1^ above the all-chair ground state. In conclusion, complete inversion in **15** requires a combination of stepwise and concerted mechanisms.

The question how many rings need to be converted to their twist form to permit complete inversion (and enantiomerization) in **2** can now be answered. A C′TCC…C conformer would have to be created by the stepwise mechanism, as described for **15** above. The internal twist ring would then travel though the structure by the concerted process, presumably as a random walk up and down **2**, but eventually inversion would be completed. Thus, complete inversion in **2** would not involve creating conformers with more than two rings in twist form at any one time and the highest energy barrier need not be significantly larger than for that of **15**. Ring inversion of **2** and **15** will be fast on the laboratory time scale at ambient temperature, but the ^13^C NMR will be in slow exchange. Fast exchange should be reached at ∼150 °C.

### Angular hydrocarbon **16** and related ladder oligomer **3**

**Fig. 4:**
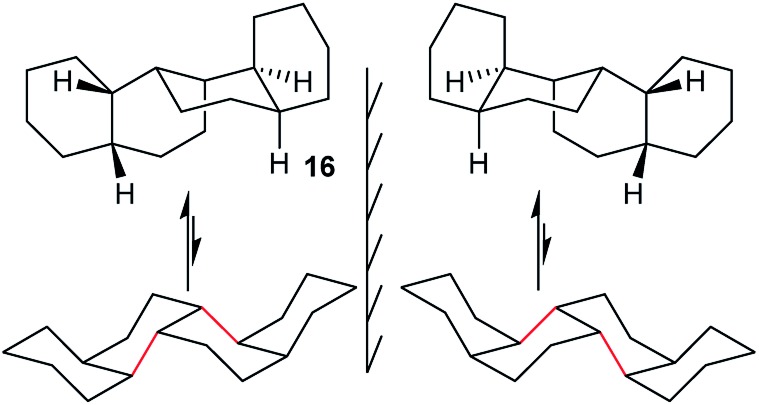
All-chair conformers for *cis*,*anti*,*cis*,*anti*,*cis*-perhydrobenzo[*c*]phenanthrene **16** and their enantiomers.

In *cis*,*anti*,*cis*,*anti*,*cis*-perhydrobenzo[*c*]phenanthrene **16**, inversion interconverts two different conformers (Fig. 4), with the lower conformer disfavoured by two extra gauche interactions associated with the bonds picked out in red; the energy difference is 12.7 (15.1) kJ mol^–1^. Enantiomers remain separate, but axial and equatorial hydrogen atoms change places. In longer structures like **3** every sequence of three rings has the same conformation so, at equilibrium, all the rings in **16** (and **3**) would be in the upper conformations of Fig. 4. While inversion in **16** (and especially **3**) will therefore be of no practical significance, it is useful to consider the choreography of this for comparison with the perhydrohelicenes discussed later.

**Fig. 5:**
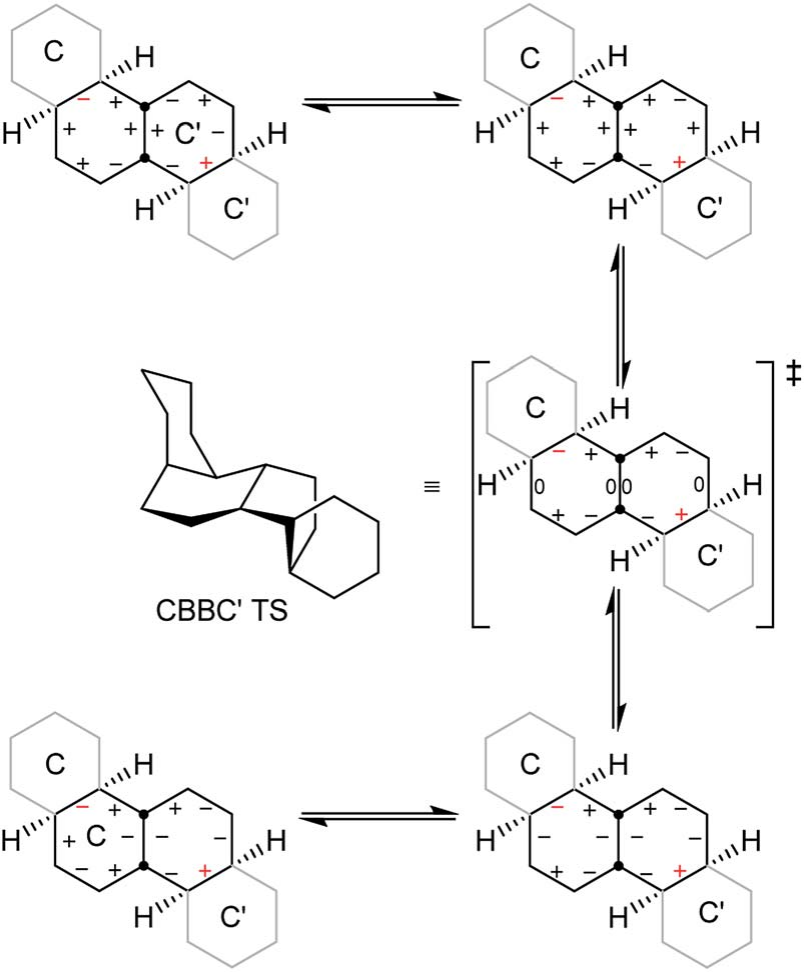
Conversion of CTC′C′ to CCTC′ for **16**. Positive and negative torsion angles in the central rings are shown as + and –, and those locked by the adjacent chair rings are shown in red.

A TS for the concerted mechanism for inversion of the inner rings of **16** can be located, Δ*E* ^‡^ is 82.5 (79.5) kJ mol^–1^ from the more stable conformer. However, with their unconstrained –CH_2_CH_2_– segments, the inner rings of **16** do have more conformational freedom and can both be twist simultaneously. A stepwise inversion process for the inner rings of **16** (and thus of **3**) is therefore possible and we show below that this is more favourable. Inversion is strictly choreographed by the flanking chair rings and this can be explained with reference to Fig. 5 below. One terminal chair ring is inverted with respect to the other, and this determines the signs of the torsion angles shown in red in the central rings. As discussed already, the twist ring in the first CTC’C’ intermediate cannot pseudorotate, and so only one CTTC′ intermediate can be formed by the chair to twist process. Pseudorotation can then only proceed *via* the CBBC′ transition state shown in the centre of Fig. 5 to the alternate CTTC′, which leads to CCTC′ by twist to chair interconversion. Baas *et al.* ^2^ showed that there were two pseudorotation pathways for *cis*-decalin itself, one with *C*_1_ symmetry, and one with *C*_2_ symmetry. The CTTC intermediates and CBBC′ TS in Fig. 5 are on the *C*_1_ pathway but only this part of that pathway can be reached. We show later that inversion in **17** and perhydrohelicenes **4** must proceed *via* intermediates on the Baas *C*_2_ pathway.

The stepwise mechanism is therefore now possible for all the rings and is lower in energy with the rate-limiting TS for inversion of the inner rings (C′TCC to C′TTC) lying only 61.8 (63.0) kJ mol^–1^ above the most stable conformer.

Before passing on to the rather special case of the perhydrohelicenes, we note that any other polycyclic *cis*-fused cyclohexane derivative *e.g.* with a random mixture of linear and angular segments should be able to undergo rolling inversion with barriers not much different from **15** and **16**. Linear segments may require the concerted mechanism, but elsewhere the stepwise mechanism is likely to dominate.

### Perhydrohelicenes: structure and strain

Structure **17** can be regarded as *cis*,*anti*,*cis*,*anti*,*cis* perhydro[4]helicene. Helicenes have been extensively studied,^12^ but saturated analogues have received limited study and the products of per-hydrogenation of the parent helicenes have not been previously discussed. Schreiner, Fokin and co-workers^13^ have reported the preparation of helically chiral [123]tetramantane, and oligomers derived from this can be consider to be rigid analogues of **17** and **4**. The properties of polytwistane, as a nonrepeating, alternating s-helix with an irrational periodicity have also been discussed.^14^ and Allen, Quanz and Schreiner^15^ have also computed the properties of polytriangulane which they describe as a σ-helicene. While fully saturated structures cannot possess the interesting chiroptical properties of aromatic helicenes, partially saturated, heterocyclic and/or functionalized derivatives of **4** may well hold significant interest, as discussed later.

Formal hydrogenation of a helicene so that the ring junctions are all *cis*,*anti*,*cis* leads to a helical structure that is based on the diamond lattice – see **4**, **17** and Fig. 1(a). A polymer would have 3_1_ helical symmetry with a repeat distance in an idealized structure exactly four times the C–C bond distance in diamond (*i.e.* 6.16 Å). For the optimized twenty-ring sequence shown in Fig. 1(a), the repeat distance is 6.3 Å and there is remarkably little strain. In particular, there are no *syn*-pentane (g^+^g^–^) interactions and, unlike helicenes, there is little long-range non-bonded repulsion.

The strain energy in the interiors of oligomers **2**, **3**, and **4** can be assessed by comparison with strain-free oligomer **9**. To do this, the MMFF force field energies for the four-ring “tetramers”, *i.e.* **15–17**, were subtracted from those for the corresponding “octamers”, the result divided by four and subtracted from the corresponding value for the corresponding all *trans*,*syn*,*trans*-oligomers **9**. This leads to a value of strain energy for **2** of 9.1 kJ mol^–1^ per ring. For **3**, the values are 9.6 kJ mol^–1^ for the more stable and 16.5 kJ mol^–1^ for the less stable conformer. For comparison, the *cis*-/*trans*-energy difference in decalin, corresponding to three extra gauche interactions, is 8.0 kJ mol^–1^. The strain energy per ring for the interior of helical oligomer **4** is 13.0 kJ mol^–1^; note that **4** contains only half the number of extra gauche interactions as does the less stable conformer of **3**.

The right-handed (RH) helix of **4** has –60° torsion angles about the central CH–CH bonds of each of the decalin units in the helix. It is based on an g^–^ag^–^ag^–^a… sequence of torsion angles for the C(H)–C(H)–C(H)–C(H) bonds along the inner rim of the helix, picked out in red in **4**. These inner rim C–C–C–C torsions are useful in understanding how ring inversions relate to the overall helix inversion.

**Fig. 6:**
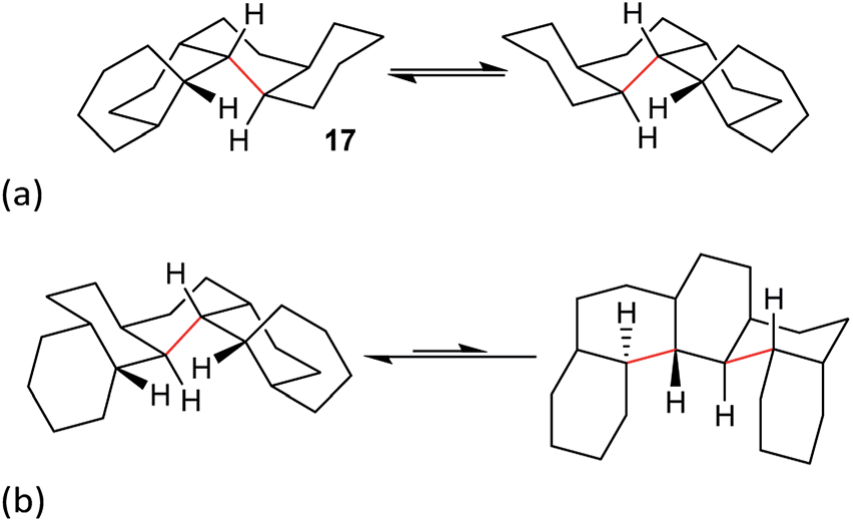
(a) Ring inversion in perhydro[4]helicene **17** results in enantiomerization, (b) ring inversion in perhydro[5]helicene favours the LH structure with one fewer extra gauche interaction (picked out in red).

Perhydrohelicenes with even numbers of rings, like **17**, have *C*_1_ symmetry but can enantiomerize *via* ring inversion. As shown in Fig. 6(a), the RH helix form of **17** has ag^–^ inner rim torsion angles; after helix inversion these become g^+^a. The two ends of **17** are not the same, so a detailed mechanism for helix inversion starting from one end of **17** is different from that if inversion starts for the other end. We return to this point later.

Perhydrohelicenes with an odd number of rings have *C*_2_ symmetry; ring inversion interconverts conformational diastereomers, see Fig. 6(b). Note that, unlike **3**, the difference in energy between the two conformers never exceeds one extra gauche bond (picked out in red in Fig. 6(b)) whatever the length of the perhydrohelicene.

*trans*-Fused rings can clearly occur in short perhydrohelicenes but, in any extended structure like **4**, the interior must have some regular repeating sequence and we believe there is no low-energy alternative to the *cis*,*anti*,*cis* sequence. Other helical sequences can clearly be found within the diamond lattice, such as g^–^g^–^g^–^g^–^… but these do not work with the fused rings discussed here. Molecular dynamics sampling of the conformational space for the 4-, 5-, 6-, 8-, and 13-perhydrohelicenes did not thrown up any exceptions to the *cis*,*anti*,*cis* sequence. Aggarwal and co-workers^16^ recently reported elegant stereoselective syntheses of several isomers of oligomeric Ar(CHMe)_*x*_OPNB, and it is interesting that the all-*syn* isomer with *x* = 10 adopts a helical conformation whose main chain is identical to the inner rim of the perhydrohelicenes discussed here.

### Helix inversion mechanisms in helicenes and perhydrohelicenes

**Fig. 7:**
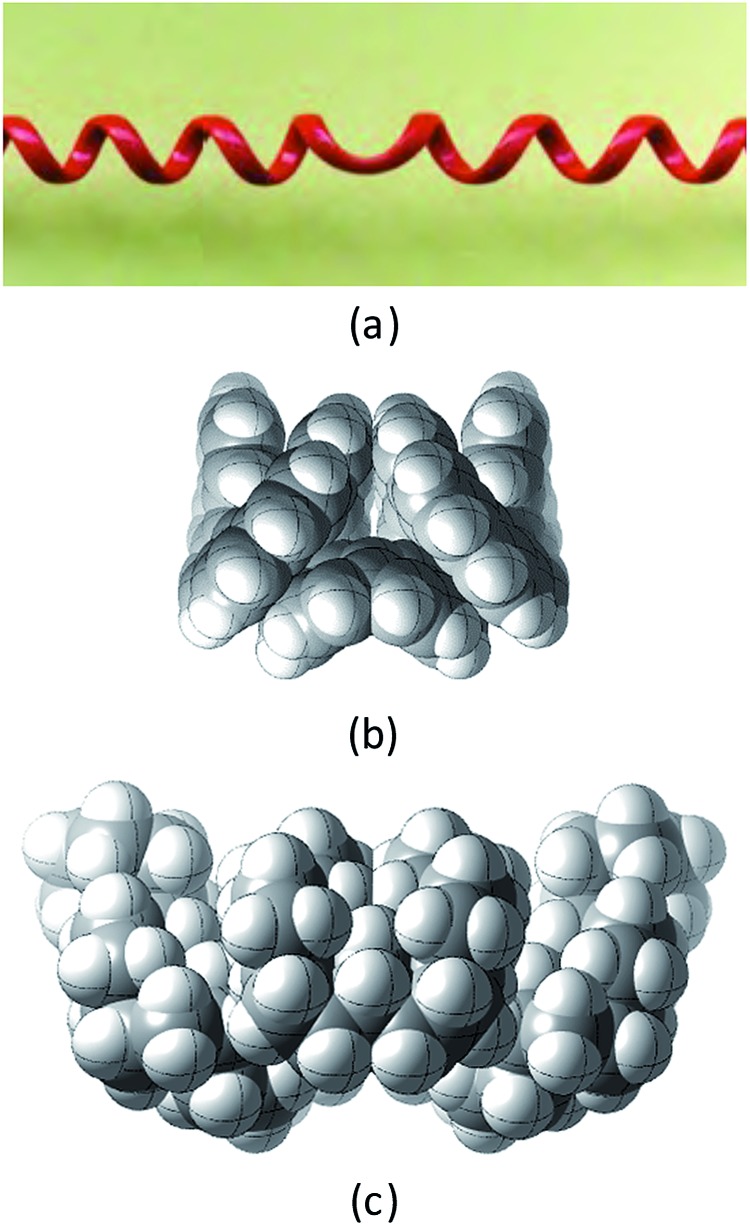
(a) A kinked telephone cable, (b) *C*_s_ TS for [20]helicene inversion, (c) *C*_s_ TS for all *cis*,*anti*,*cis* perhydro[20]helicene inversion.

The inversion of helices (reversal of screw-sense) necessarily involves intermediate structures that resemble a kinked old-style telephone cable where the sense of the helix changes sign, see Fig. 7(a). Partially inverted helices are frequently observed in the botany of tendrils, as noted by Darwin^17^ and called perversion. Fig. 7(b) and (c) show structures with *C*_s_ symmetry that lead to enantiomerization of [20]helicene and perhydro[20]helicene.

The image of the *C*_s_ TS for [20]helicene in Fig. 7(b) shows that there must be severe non-bonded interactions between the rings adjacent to and on either side of the inverting rings. This and the consequential extra bending of the aromatic rings is the principal source of the high barrier to inversion in helicenes. As one moves further away from the inverting rings, the structure gradually resumes the normal helical structure. It can therefore be expected that the barrier to inversion will approach an asymptotic limit as the number of rings increase. Although perhydrohelicenes, Fig. 7(c), are more complex, once again there are strong non-bonded interactions between the rings adjacent to and on either side of the inverting rings, and an asymptotic approach to a limiting barrier is also to be expected.

There has been limited computational work on inversion barriers in helicenes.^18–20^ We have located a *C*_s_ TS (one imaginary frequency displaying a rocking motion) for inversion at the central two rings of [12]helicene at the B3LYP/6-31G* level with a barrier of 274.9 (243.4) kJ mol^–1^. For [20]helicene, we have been unable to calculate vibrational frequencies but the energy difference is now 313.4 (275.4) kJ mol^–1^. For an infinite helicene this energy difference might be around 320–350 kJ mol^–1^. Barriers will be low near each end and build up to that found for the centre, so we suggest that this value is a reasonable estimate for the limiting overall energy barrier. Between each TS, there will be at least one intermediate. For [12]helicene we located the minimum adjacent to the central *C*_s_ structure. This lies just 19 kJ mol^–1^ below the *C*_s_ structure. The reaction profile for helicenes is therefore likely to be fairly smooth, a contrast with the perhydrohelicenes, as discussed below.

### Helix inversion mechanisms in perhydrohelicenes

For perhydrohelicenes, intermediates and transition states in the inversion still have shapes (Fig. 7(c)) broadly like kinked cables, but the rolling process is superimposed on this. Just as with **16** above, adjacent chair rings on either side control the inversion process, but the choreography for perhydrohelicenes is more complex and intriguing. In terms of the C(H)–C(H)–C(H)–C(H) torsion angles along the inner rim of the helix described earlier, complete helix inversion corresponds to conversion of g^–^ag^–^ag^–^a… to ag^+^ag^+^ag^+^a…. Inversion sequences for [6]-, [7]-, and [8]-perhydrohelicene are shown below. Because the two termini of a perhydrohelicene with an even number of rings are different, starting the inversion from one end of the structure results in a different sequence from that resulting from starting at the other end. There is only one sequence however for perhydrohelicenes with odd numbers of rings. Torsion angle listings in square brackets (*e.g.* [a***g*** ^***–***^ ***g*** ^***+***^a]) correspond to TS structures with an eclipsed bond between two boat-form rings (torsion angles within boat rings are given in bold italics).
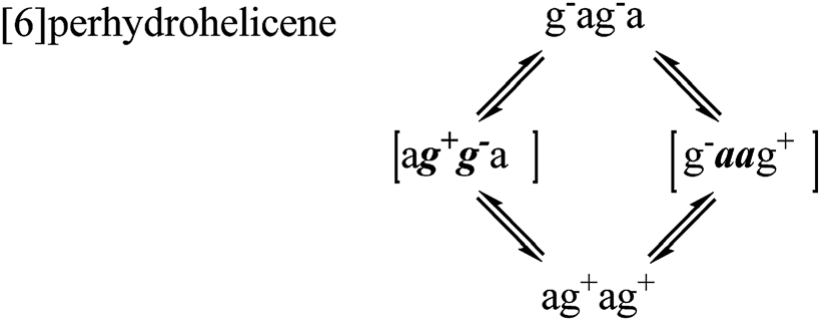
 
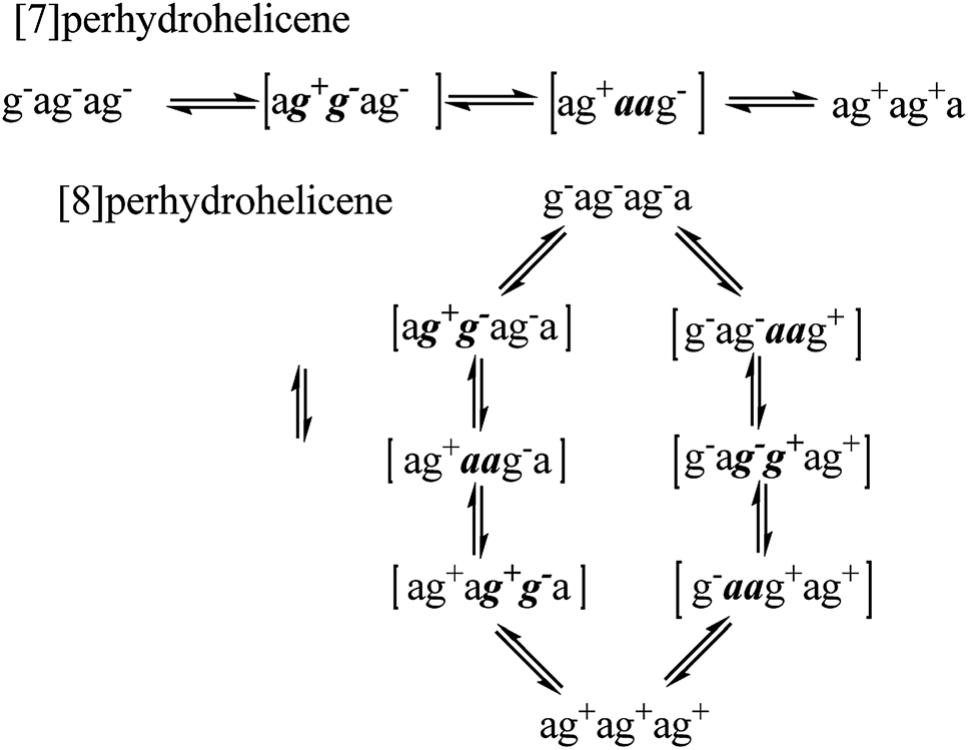


As the rolling inversion process proceeds along the chain of rings, individual inversions alternate between the [a***g*** ^***–***^ ***g*** ^***+***^a] and [g^+^ ***aa***g^–^] cases. This alternation is associated with the [g^–^ag^–^ag^–^a…] sequence of torsion angles along the inner rim of the ground state and, ultimately, the *cis*,*anti*,*cis*,*anti*… sequence of ring fusions.

**Fig. 8:**
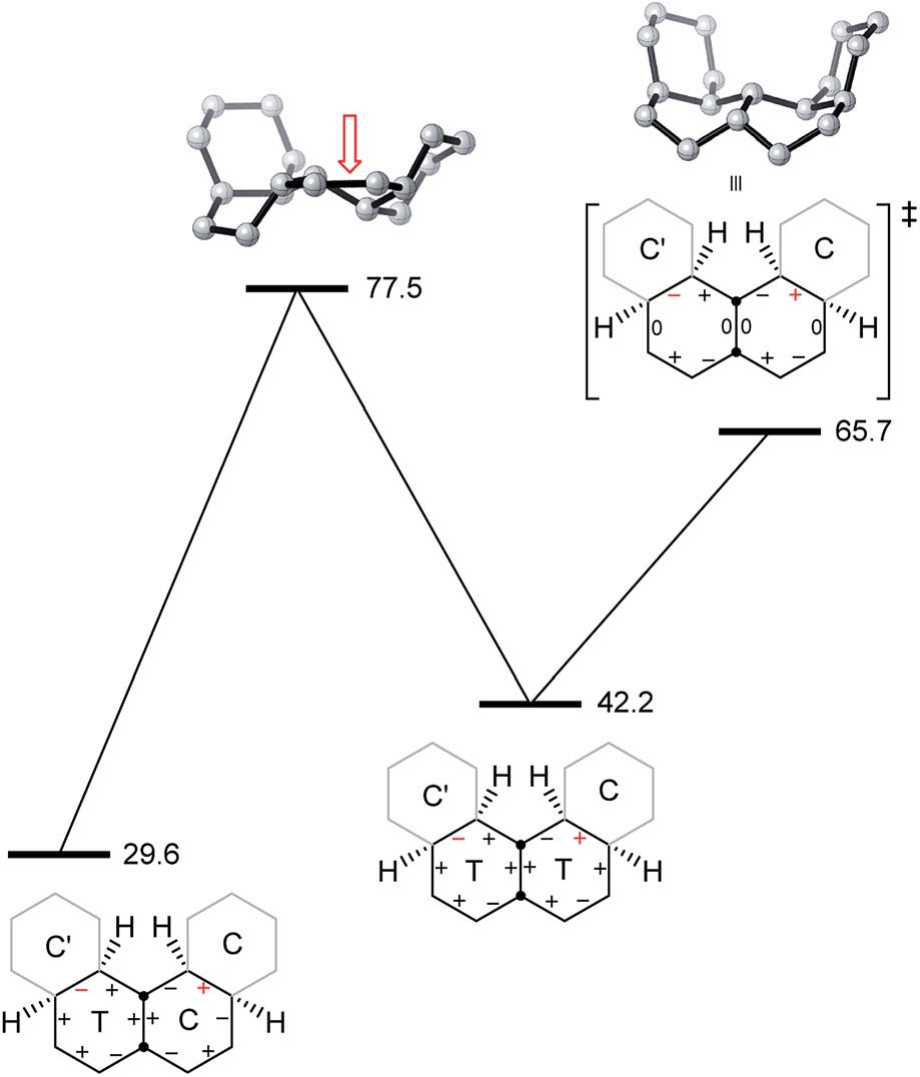
Potential energy diagram for the [a***g*** ^***–***^ ***g*** ^***+***^a] pathway from C′TCC to the *C*_s_ TS for enantiomerization of **17**; energies (in kJ mol^–1^) are relative to the CCCC global minimum. Torsion angles that are controlled by the adjacent chair rings are shown in red. The red arrow highlights the half-chair conformation of the ring undergoing the chair-to-twist change in the first TS.

**Fig. 9:**
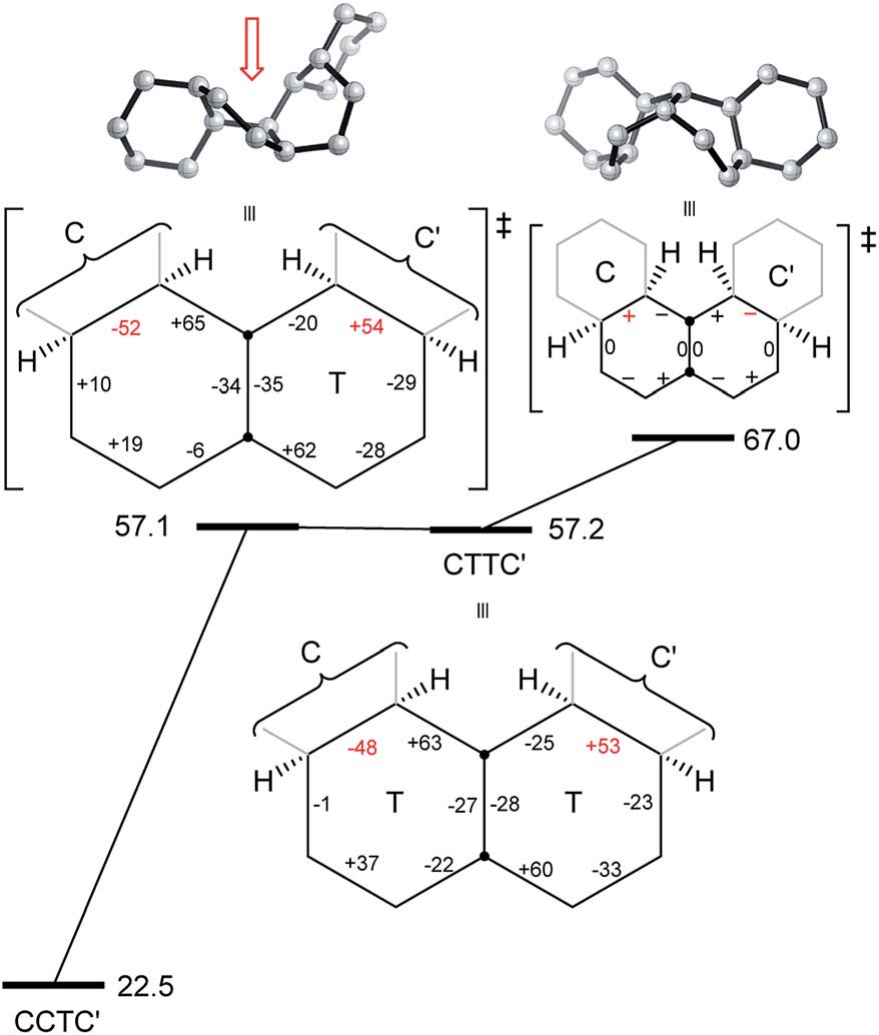
Potential energy diagram for the [g^+^ ***aa***g^–^] pathway from CCTC′ to the *C*_s_ TS for enantiomerization of **17**; energies (in kJ mol^–1^) are relative to the CCCC global minimum. Torsion angles that are controlled by the adjacent chair rings are shown in red. The red arrow highlights the half-chair conformation of the ring undergoing the chair-to-twist change in the first TS. Torsion angles within the two twist rings are shown for the CTTC′ intermediate and the chair-to-twist TS.

We now examine the [a***g*** ^***–***^ ***g*** ^***+***^a] and [g^+^ ***aa***g^–^] pathways for the simplest example: *cis*,*anti*,*cis*,*anti*,*cis*-perhydro[4]helicene **17**. These are illustrated in Fig. 8 and 9 respectively. The [a***g*** ^***–***^ ***g*** ^***+***^a] pathway (Fig. 8) corresponds to the classic stepwise process, essentially the same as established for *cis*-decalin itself, with the chair-to-twist process being rate-limiting, although the activation energies are raised by extra non-bonding interactions involving the other rings. The [a***g*** ^***–***^ ***g*** ^***+***^a] pathways for the larger perhydrohelicenes (6-, 8-, 10-, and 12-) are identical in general form.

The [g^+^ ***aa***g^–^] pathway for **17** is significantly different, as shown in Fig. 9. Now the rate-limiting step involves the *C*_s_ enantiomerization process, and an activated chair-to-twist TS can barely be distinguished! This behaviour is puzzling when is it found that for the corresponding conformation of *cis*-decalin itself the pathway is “normal” *i.e.* stepwise and like that in Fig. 8. The explanation for the behaviour seen in Fig. 9 comes from careful examination of the torsion angles within the central rings of the chair-to-twist TS and the CTTC′ intermediate (see Fig. 9). The CTTC′ intermediate is structurally very similar to the “half-chair” conformation of the TS; note especially the sequence of small torsion angles in the left-hand ring. An almost unactivated conversion to the TS and thus on down to the CCTC′ structure is therefore understandable, and indeed this process can easily be followed using a torsion angle driver in molecular mechanics. Animation of the computed lowest energy vibration for the intermediate (51 cm^–1^) shows it to be essentially identical to the imaginary vibration (109 cm^–1^) of the TS. Noted that the potential energy of the TS (Fig. 9) is actually slightly lower than that of the CTTC′ intermediate, although Δ*G* is higher, of course.

We found that, in the corresponding pathways for all the larger perhydrohelicenes, distinct CTTC′ intermediates and chair-to-twist TSs could not be found despite extensive searches; the *C*_s_ enantiomerization TS goes straight down to a CCTC′ structure. The whole process is thus effectively concerted, though not for the same reason as in the case of **14** above.

The alternative BB transition states with *C*_s_ symmetry for **17** seen in Fig. 8 and 9 have essentially the same potential energy. While the [a***g*** ^***–***^ ***g*** ^***+***^a] structure would be expected to be lower in energy as far as the boat rings themselves are concerned, the [g^+^ ***aa***g^–^] structure avoids a g^+^g^–^ interaction between the outer chair rings as can be seen by comparison of Fig. 8 and 9 (see also Table 1 below).

**Table 1:** Perhydrohelicenes activation energies^*a*^

No. of rings	4	6	8	10	12
CT to TT TS for [a***g*** ^*–*^ ***g*** ^*+*^a]	77.5	71.9	94.5	90.4	94.9
[a***g*** ^*–*^ ***g*** ^*+*^a] *C*_s_ TS	65.7	68.9	82.2	74.7	80.5
[g^+^ ***aa***g^–^] *C*_s_ TS	67.0	79.2	76.9	80.9	75.4

^*a*^B3LYP/6-31G*//B3LYP-gCP-D3/6-31G* activation energies (kJ mol^–1^) for the ring inversion processes associated with the central pair of rings in perhydrohelicenes containing 4, 6, 8, 10, and 12 rings.

As a footnote to this discussion, we note that the concerted mechanism described for **14** earlier is possible and competitive for perhydro[4]helicene, **17**; the *C*_s_ TS is 73.4 (66.5) kJ mol^–1^ above the all-chair ground state. The concerted mechanism rapidly becomes unfavourable however in larger perhydrohelicenes as models show that it leads to even more severe non-bonded interactions between chair rings near the inverting centre than the [g^+^ ***aa***g^–^]-type structure.

It is helpful to examine physical molecular models to appreciate what is going on in these inversions. As discussed earlier, C′TC intermediates can barely pseudorotate due to the fusion with two chair-form rings, and the inner rim torsion is close to 120°. On one side of this ring will be a chair with an internal C(H)–C(H)–C(H)–C(H) torsion which is anti while the chair on the other side the internal C(H)–C(H)–C(H)–C(H) torsion will be gauche. Flipping the former to a twist leads to the [a***g*** ^***–***^ ***g*** ^***+***^a] structure with (local) *C*_s_ symmetry, while flipping the latter gives the [g^+^ ***aa***g^–^] structure. What is clear is that complete helix inversion requires passage through both types of boat, boat structures.

With the choreography of the inversion processes now clear, we can turn to the results from calculations. Our principle aim has been to obtain an estimate of the limiting value for the barrier to helix inversion in a long perhydrohelicene. We have therefore not attempted to follow these helix inversion sequences all the way by calculation, but have concentrated on optimising (at the B3LYP/6-31G* level) the TS structures associated with ring inversion at the central rings of perhydrohelicenes containing 4, 6, 8, 10, and 12 rings and the related intermediates. Activation energies for the three significant TSs (see Fig. 8 and 9) are listed in Table 1.

The data in Table 1 shows that the barriers do increase as the length of the perhydrohelicenes increase, but it is notable that none of the barriers are a smooth function of the number of rings. We have no complete explanation of this, but have identified one factor that is surely partially responsible. The [ag^+^ ***aa***g^–^a] *C*_s_ TS for perhydro[8]helicene has only 2 extra gauche interactions along its inner rim, whereas the [g^–^a***g*** ^***–***^ ***g*** ^***+***^ag^+^] *C*_s_ TS has 4, the same as for the ground state: [g^–^ag^–^ag^–^ag^–^]. This is the situation for perhydrohelicenes containing 4*n* rings, whereas for perhydrohelicenes with 4*n* + 2 rings, the number of extra gauche interactions is the same for both *C*_s_ transition states. Note that if we had examined the TSs for inversion involving the pair of rings one removed from the middle of the chain, the situation would have been reversed.

We have not studied perhydrohelicenes with odd numbers of rings computationally, but it seems unlikely that the activation energy for, say, perhydro[7]helicene is very different from that for the average of perhydro[6]- and perhydro[8]helicene. As noted for the helicenes above, it seems probable that the energy barriers associated with the central section of the helix will be the highest, or at least be a good approximation to the highest barrier.

The half-life for enantiomerization of perhydro[12]helicene can be estimated at about 14 h at 298 K so it is unlikely that any unsubstituted perhydrohelicene will be optically stable at ambient temperatures. It should be possible however to follow the inversion of perhydrohelicenes by ^13^C NMR. The computed spectrum for **17** shows two signals at 24.9 and 25.7 ppm that are >3 ppm upfield of the remainder. These will average with those at 31.8 and 32.1 ppm respectively when inversion is fast.
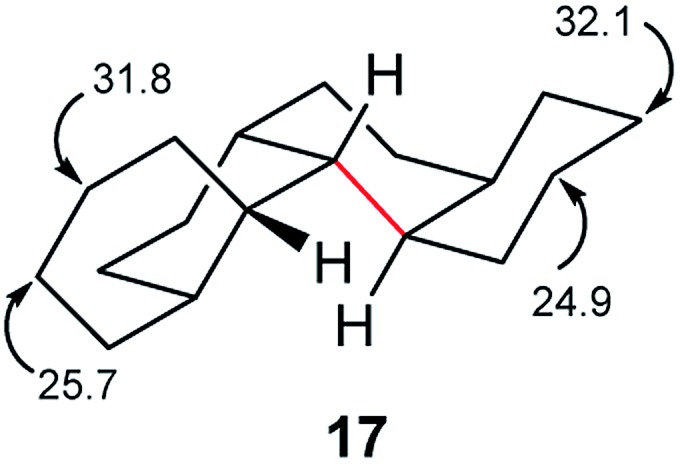


Perhydrohelicenes containing 6, 8, 10, and 12 rings also show corresponding signals in their terminal rings that are clearly upfield of the remainder of the spectrum, so finding the onset of fast inversion should be straightforward. Based on computed rate constants, we suggest that the onset of coalescence in the NMR spectrum should occur at about 400 K for **17**, rising to over 500 K for perhydro[12]helicene.

Perhydrohelicenes are very intriguing. Many of the conclusions drawn above would be expected to apply to all structures with the general formula 18 below, the *cis*,*anti*,*cis*,*anti*… sequence of ring fusions being more or less fundamental to the helical shape and potential for inversion. It is worth noting that large groups in the X positions of **18** are forced towards the interior during the helix inversion process, so barriers are likely to rise significantly in such cases. Five-membered and seven-membered (or larger) rings seem possible and many derivatives and hetero-analogues can be imagined such as polyaniline derivative **19**. Molecular mechanics models for a cyclopentane analogue *i.e.* **18** (X = CH_2_) show a much wider and more flexible helix than **4**, with some free space down the middle and a multitude of conformations with closely similar energies, although we have not attempted any DFT calculations.
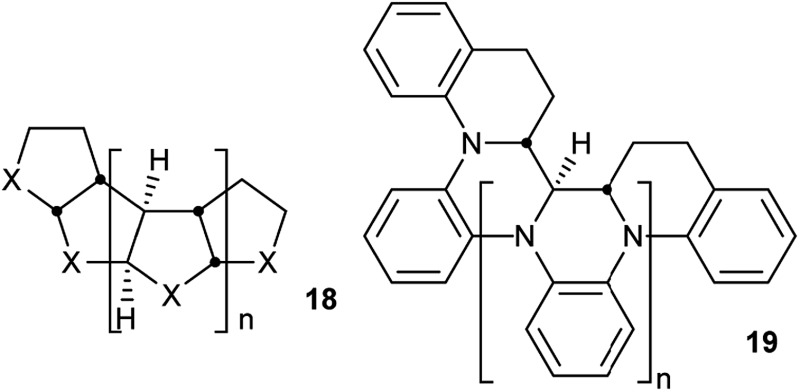


Synthesis of any perhydrohelicene would obviously be very challenging, but a possible route to an aminal derivative is suggested below (we have confirmed that the aminal shown would adopt the same conformation as **3**).
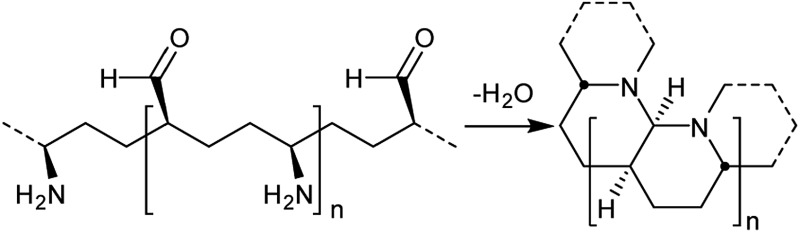


Elegant examples of screw-sense inversion in helices (and with a comparable barrier: 70 kJ mol^–1^) have recently been described by Clayden and co-workers.^21^ These oligoureas of meso cyclohexane-1,2-diamine form helices through hydrogen-bonding and so are very different structurally, but overall inversion again involves ring inversion of each of the cyclohexane rings.

### Inversion mechanism for **7**

It was stated in the introduction that rolling inversion fails for **6** and **7**. While rolling inversion only requires two adjacent rings to be in twist boat conformations at any instant, inversion in propellane **6** clearly requires that all three rings be converted to twists, with chair-to twist conversion of the last ring being rate-limiting. An experimental barrier, Δ*G* ^‡^ 65.3 kJ mol^–1^, has been reported for **6**.^22^ At the B B3LYP/6-31G*//B3LYP-gCP-D3/6-31G* level the barrier (Δ*E* ^‡^) is 71.1 (69.8) kJ mol^–1^ for **6**. Can structures be found that require more than 3 rings to be in twist boat conformations at any instant? The only example we have been able to come up with that does not also introduce (g^+^g^–^) interactions is **7**, which can be regarded as *trans*,*trans*,*trans*,*trans*-[6.6.6.6]fenestrane. The chemistry of fenestranes have been extensively investigated,^23^ but this has largely focussed on the all-*cis* isomers where the central carbon atoms may approach planar tetra-coordinate geometry; we are not aware of any studies related to **7**.

**Fig. 10:**
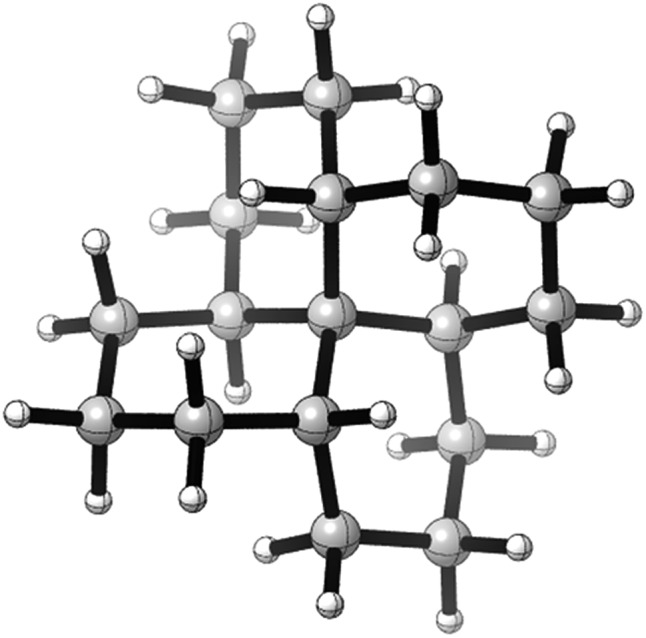
*S*_4_ symmetric hydrocarbon **7** (systematic name hexadecahydronaphtho[1,8-*de*]naphthalene).

The beautiful *S*_4_ symmetric hydrocarbon **7,** Fig. 10, is the most stable stereoisomer of this gross structure, in spite of being built of *cis*- rather than *trans*-decalins. No additional *cis*-fused rings can be added to **7** without introducing *syn*-pentane (g^+^g^–^) interactions that would destabilize the ground state. Hydrocarbon **7** is of course achiral but, just like in cyclohexane itself, inversion is a topomerization process, *i.e.* it interchanges the axial and equatorial hydrogen positions in the CH_2_ groups, and so should be easily detected by ^1^H NMR.

All our attempts to find a rolling inversion process for **7** failed. *A priori*, one might imagine that, in a TCCC conformation, the twist ring might move round the structure by rotation about an adjacent C_q_–CH bond, but this is not supported by examination of models. Building a conformation for **7** from the core structure shown in Fig. 2(c) is only possible if the added rings are in true boat conformations with severe interactions between these rings, more or less assuring a very high energy structure. Optimizing this structure under *C*_s_ symmetry with B3LYP/6-31G* leads to an energy 181 kJ mol^–1^ above the global minimum. It seems that inversion in **7** must be entirely stepwise.

Fourteen minima with energies less than 100 kJ mol^–1^ above the CCCC ground state were located for **7** by conformational searches. These are shown with their B3LYP/6-31G* energies in the ESI.† In addition, several TTTT conformations of higher energy were found (a TTTT conformation with *S*_4_ symmetry is easily constructed but is very high in energy and has 3 imaginary frequencies).

**Fig. 11:**
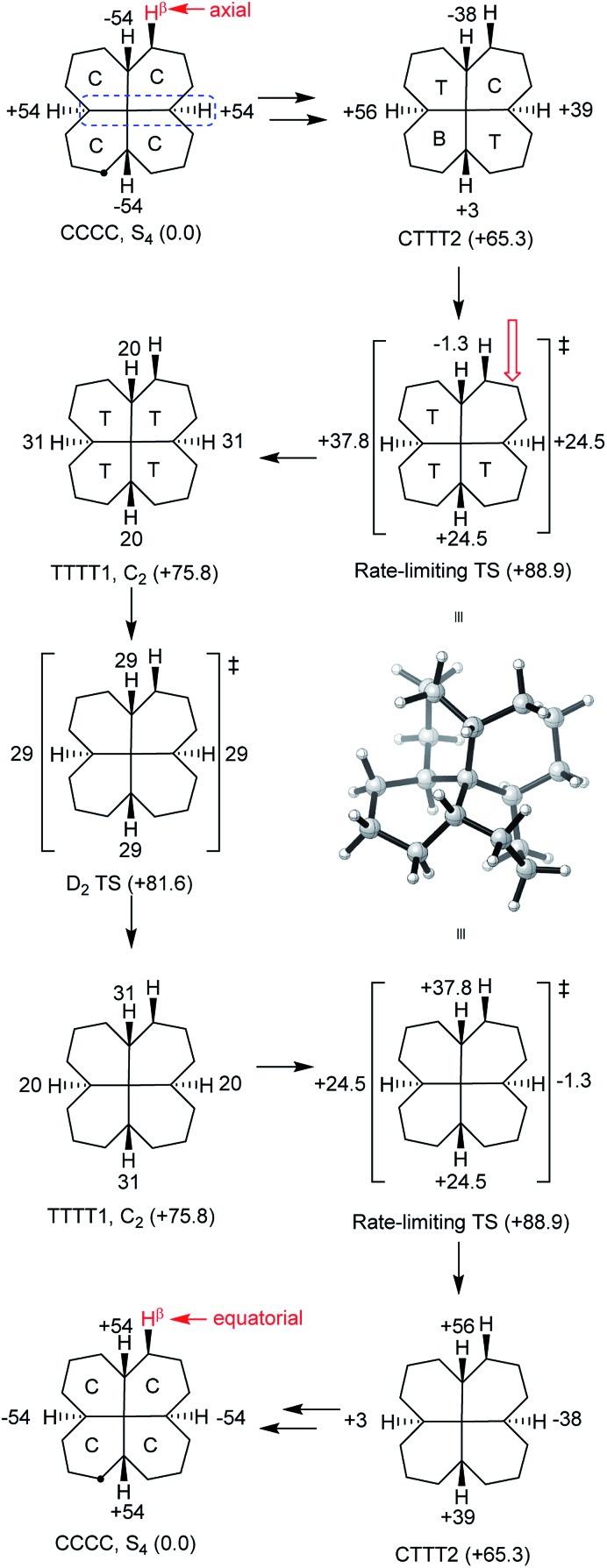
Proposed mechanism for chair-to-chair inversion in **7**. C–C_q_–C–H torsion angles are shown and one example is picked out in blue in the top left structure. The red open arrow shows the ring that is flipping in the rate-limiting TS. Relative B3LYP-gCP-D3/6-31G*//B3LYP/6-31G* energies are listed in parentheses under the structures and a perspective view of the rate-limiting TS is shown in the center.

The lowest energy inversion mechanism for **7** is shown in Fig. 11, and this mechanism resembles the *cis*-decalin proposal of Baas *et al.* ^2^ *i.e.* that the rate-determining process will involve conversion of the last chair ring to a twist conformation. The rate-limiting TS (Δ*E* ^‡^ +88.9 (87.3) kJ mol^–1^) is for conversion of CTTT2, the second lowest energy structure with three twist rings to TTTT1, the lowest energy all-twist structure. We found several other TSs for conversion of CTTT conformations to TTTT, but these were ≥9 kJ mol^–1^ higher in energy than that shown in Fig. 11. The TS for conversion of TTTT1 to itself but with the *C*_2_ axis rotated through 90° has *D*_2_ symmetry and is the symmetry point in the overall inversion scheme. By following the C–C_q_–C–H torsion angles through Fig. 11, it can be seen that this sequence provides a smooth pathway for ring inversion. It is notable that inversion of **7** shows no sign of the choreography found for **2**, **3** or **4**. There are many pathways from CCCC up to CTTT2, *via* CCCT and the CCTT and/or CTCT conformations, but these have not been investigated in detail, since the TSs for these are certainly lower in energy than the highest TS depicted in Fig. 11.

As already noted, inversion in **7** should be observable by ^1^H NMR, and might be conveniently studied by NOESY (EXSY) spectroscopy. Dynamic ^13^C NMR could also be used, since in the *S*_4_ ground state, the CH_2_ carbon atoms adjacent to the CH groups in each ring are non-equivalent. The calculated rate constants suggest that temperatures in the range of 100–200 °C will be required in order to observe this exchange.

**Fig. 12:**
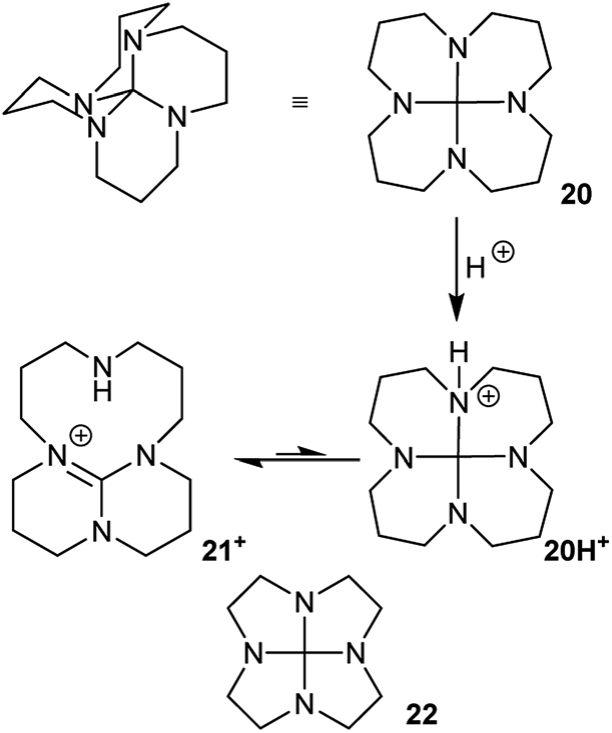
3,6,9,12-Tetraaza-hexadecahydronaphtho[1,8-*de*]naphthalene **20**, its protonation to TBD derivative **21^+^**, and lower homologue **22**.

Hydrocarbon **7** is unknown, and it would be a challenging target for synthesis. Probably the most accessible, and indeed interesting analogue would be the 3,6,9,12-tetraaza-derivative **20**, Fig. 12. This might be prepared from 1,5,9,13-tetraazacyclohexadecane^24^ as suggested in Fig. 12 *via* **21^+^**; a route that is already established^25^ for the lower homologue **22**. Compound **20** could potentially adopt other structures *via* nitrogen inversion, but calculations confirm that the *S*_4_ geometry is again strongly favoured; each lone pair is antiperiplanar to one C–N bond, so the operation of the anomeric effect will be limited. Ring inversion in **20** should be readily observable by NMR.

As has been already demonstrated for **22**,^25^ protonation of **20** should lead to rapid C–NH^+^ bond cleavage of **20H^+^** to the bridged 1,5,7-triazabicyclo[4.4.0]dec-5-ene (TBD) derivative **21^+^**. The best conformer of **21^+^** is calculated to be 78 kJ mol^–1^ more stable than **20H^+^**; the activation energy is <3 kJ mol^–1^ (B3LYP/6-31G*). As a result of this, **20** is predicted to behave as an exceptionally strong base with a proton affinity (PA) of 1091 kJ mol^–1^, comparable to the PA of NHC carbenes^26^ and well above the PA of TBD itself (1066 kJ mol^–1^ at B3LYP/6-31G*) or most proton sponges. The calculated PA for **22** (1097 kJ mol^–1^) is slightly higher than for **20**.

## Conclusions

Complete inversion of a range of multi-ring cyclohexane derivatives (**2**, **3**, **4**, **6**, and **7**) has been studied. Oligomers **2**, **3** and **4** invert by a process we call rolling inversion with no more than 2 rings in twist conformations at any one time. The sequences of conformational changes in these twist rings are shown to be precisely choreographed according to the positioning of the adjacent chair rings. We suggest that this choreography is likely to be found to apply in a wider range of similar structures.

*cis*,*anti*,*cis*-Perhydrohelicenes **4** are compared with helicenes **5**. In their ground states **4** are remarkably strain-free, a complete contrast with **5**, but complete screw-sense inversion of **4** does resemble that for **5** in that intermediates and transition states have shapes broadly like kinked old-style telephone cables. Limiting barriers for **4** are substantial (*ca.* 120 kJ mol^–1^) but much lower than for **5** (320–350 kJ mol^–1^).

Propellane **6** and *S*_4_ symmetric hydrocarbon **7** cannot partake of rolling inversion and all rings must be simultaneously in twist conformations to achieve overall inversion, and so **7** probably has the highest inversion barrier for any non-polymeric multi-ring structure built from cyclohexane rings. It is notable that inversion of **6** and **7** is not subject to the choreography seen for **2**, **3** and **4**.
