# Supplementary material for: Perhydrohelicenes and other diamond-lattice based hydrocarbons: the choreography of inversion
Source: Chem Sci. 2017 Jul 17;8(9):6389–99. doi: 10.1039/c7sc01759f (PMC5628603; doi:10.1039/c7sc01759f)
Supplement: Supplementary file 1 [file SC-008-C7SC01759F-s001.pdf]

Electronic Supplementary Material (ESI) for Chemical Science.
This journal is © The Royal Society of Chemistry 2017

Perhydrohelicenes and other diamond-lattice based hydrocarbons: the choreography of inversion

Roger W. Alder and Craig P. Butts
School of Chemistry
University of Bristol
Bristol
BS8 1TS

Richard B. Sessions
School of Biochemistry
Medical Sciences Building
University Walk
Bristol
BS8 1TD

ELECTRONIC SUPPLEMENTARY INFORMATION

Table of Contents

Perhydrohelicenes and other diamond-lattice based hydrocarbons: the choreography of inversion 1

ELECTRONIC SUPPLEMENTARY INFORMATION 1

Full citation for reference 3 3

Pseudorotation in CT cis-decalin 3

Energies and Geometries 4

(4aR,8aR,9aS,10aS)-Dodecahydro-4a,9a-butanoanthracene 4

trans,cis,trans-Perhydrotetracene 14 5

Perhydrotetracene 15 7

Perhydrochrysene 16 9

Helicenes 12

Helicene 12 12

Helicene 20 13

Perhydrohelicenes 16

B3LYP/6-31G* activation energies 16

Perhydro[4]helicene 16

Perhydro[6]helicene 19

Perhydro[8]helicene 22

Perhydro[10]helicene 26

Perhydro[12]helicene 30

Perhydro[20]helicene 36

Propellane 6 40

S4 hydrocarbon 7 41

Structures and relative B3LYP/6-31G* energies (kJ mol^{-1}) for conformers of hexadecahydronaphtho[1,8-de]naphthalene 7 45

Full citation for reference 3

Gaussian 09, Revision B.01, M. J. Frisch, G. W. Trucks, H. B. Schlegel, G. E. Scuseria, M. A. Robb, J. R. Cheeseman, G. Scalmani, V. Barone, B. Mennucci, G. A. Petersson, H. Nakatsuji, M. Caricato, X. Li, H. P. Hratchian, A. F. Izmaylov, J. Bloino, G. Zheng, J. L. Sonnenberg, M. Hada, M. Ehara, K. Toyota, R. Fukuda, J. Hasegawa, M. Ishida, T. Nakajima, Y. Honda, O. Kitao, H. Nakai, T. Vreven, J. A. Montgomery, Jr., J. E. Peralta, F. Ogliaro, M. Bearpark, J. J. Heyd, E. Brothers, K. N. Kudin, V. N. Staroverov, T. Keith, R. Kobayashi, J. Normand, K. Raghavachari, A. Rendell, J. C. Burant, S. S. Iyengar, J. Tomasi, M. Cossi, N. Rega, J. M. Millam, M. Klene, J. E. Knox, J. B. Cross, V. Bakken, C. Adamo, J. Jaramillo, R. Gomperts, R. E. Stratmann, O. Yazyev, A. J. Austin, R. Cammi, C. Pomelli, J. W. Ochterski, R. L. Martin, K. Morokuma, V. G. Zakrzewski, G. A. Voth, P. Salvador, J. J. Dannenberg, S. Dapprich, A. D. Daniels, O. Farkas, J. B. Foresman, J. V. Ortiz, J. Cioslowski, and D. J. Fox, Gaussian, Inc., Wallingford CT, 2010.

Pseudorotation in CT cis-decalin

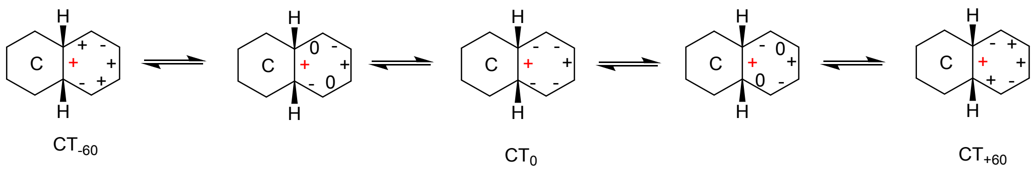

After Figure 9 in J. M. A. Baas, B. van de Graaf, D. Tavernier, and P. Vanhee, *J. Am. Chem. Soc.*, **1981,** *103,* 5014-5021.

Energies and Geometries

Energies and geometries (x, y, z coordinates) for all ground states and transition states discussed in the paper are listed below. These are presented in order of their appearance in the paper. B3LYP/6-31G* electronic energies in a.u. (labelled DFT energy) are followed by gCP and D3 corrections and single point B3LYP-gCP-D3/6-31G* energies (labelled DFT-gCP-D3 energy))

(4aR,8aR,9aS,10aS)-Dodecahydro-4a,9a-butanoanthracene

Ground state

DFT energy	-704.009688278
gCP correction	0.1290623392
D3 correction	-0.10753933
gCP-D3 correction	0.0215230092
DFT-gCP-D3 energy	-703.9881652688

H 3.100571 -0.398819 1.085350
C 2.335630 0.335528 1.366815
C 2.857363 1.819073 -0.607368
C 0.907498 0.088761 -0.775906
C 1.479838 1.478197 -1.191125
C 0.956444 -0.113622 0.791427
C 2.826675 1.718957 0.919894
H 3.147169 2.830575 -0.921414
H 1.515582 1.514964 -2.289644
H 3.824270 1.903216 1.339444
H 2.274223 0.298501 2.463751
H 3.627737 1.144995 -1.004534
H 0.793679 2.277698 -0.889056
H 2.177262 2.507864 1.322977
C 0.772851 -1.613511 1.175860
H 0.936381 -1.706955 2.259023
H -0.261886 -1.921469 1.005123
C -0.561206 -0.006296 -1.285862
H -0.559820 0.181165 -2.370259
H -0.927420 -1.032567 -1.162714
C -0.144701 0.737218 1.499498
H 0.191261 1.780924 1.514695
H -0.184806 0.429096 2.554720
C 1.757509 -0.991520 -1.507648
H 2.823755 -0.792831 -1.338254
H 1.599426 -0.874857 -2.589092
C -1.581924 0.751062 0.917118
H -2.072581 1.636319 1.353252
C -1.537862 0.974771 -0.611969
H -1.147183 1.987107 -0.773920
C 1.488169 -2.441702 -1.090637
H 2.166190 -3.111029 -1.636256
H 0.469938 -2.744082 -1.372994
C 1.668536 -2.605579 0.421172
H 1.418508 -3.628547 0.732040
H 2.725584 -2.458923 0.682409
C -2.480108 -0.438184 1.320119
H -2.522774 -0.501406 2.415955
H -2.062980 -1.390251 0.975796
C -2.952681 0.967869 -1.235043
H -2.871669 1.000080 -2.330211
H -3.460294 1.897431 -0.937752
C -3.892471 -0.304142 0.733014
H -4.373332 0.601756 1.132034
H -4.514932 -1.153122 1.043927
C -3.828970 -0.213808 -0.797200
H -4.836528 -0.102663 -1.218476
H -3.427916 -1.154634 -1.200296

Transition state for inversion

DFT energy	-703.982605190
gCP correction	0.1284312912
D3 correction	-0.10642257
gCP-D3 correction	0.0220087212
DFT-gCP-D3 energy	-703.9605964688

H 1.454400 -1.391000 -2.388800
C 1.397800 -1.410700 -1.291500
C 2.791000 -1.683900 0.838400
C 0.882000 0.057900 0.801800
C 1.419800 -1.274600 1.389800
C 0.829100 -0.042000 -0.810500
C 2.776000 -1.768300 -0.712200
H 3.082600 -2.646800 1.275100
H 1.470600 -1.178400 2.482700
H 3.541600 -1.103900 -1.130200
H 0.699500 -2.218800 -1.046700
H 3.548700 -0.963100 1.170400
H 0.706500 -2.084300 1.195200
H 3.046800 -2.777200 -1.046400
C 1.634900 1.058200 -1.582400
H 2.613900 0.656300 -1.873900
H 1.110000 1.262200 -2.525100
C -0.549600 0.291600 1.369600
H -0.483100 0.284000 2.467300

H -0.856800 1.311600 1.104800
C -0.645000 0.066400 -1.300100
H -0.653900 -0.130900 -2.381600
H -0.981200 1.106000 -1.186100
C 1.746100 1.237800 1.396500
H 2.331200 0.856000 2.242600
H 1.055500 1.971900 1.832100
C -1.646200 -0.884000 -0.620400
H -1.313700 -1.908100 -0.829500
C -1.652900 -0.687600 0.934600
H -1.450200 -1.658200 1.404400
C 2.661900 2.019800 0.451300
H 3.558800 1.438400 0.193300
H 3.014600 2.924700 0.963300
C 1.906600 2.359300 -0.830800
H 0.970600 2.883500 -0.586300
H 2.488400 3.037200 -1.468600
C -3.061800 -0.764200 -1.222900
H -3.662200 -1.610400 -0.863100
H -3.008700 -0.877200 -2.313900
C -3.032700 -0.221300 1.459300
H -2.938200 0.086500 2.509400
H -3.728800 -1.070800 1.455700
C -3.785000 0.548600 -0.869900
H -4.845000 0.444900 -1.132300
H -3.401400 1.363300 -1.495700
C -3.634000 0.925300 0.630900
H -4.604400 1.212300 1.053300
H -2.995100 1.811700 0.728600

trans,cis,trans-Perhydrotetracene 14

Ground state (CTCC)

DFT energy	-704.024654622
gCP correction	0.1257080926
D3 correction	-0.09888429
gCP-D3 correction	0.0268238026
DFT-gCP-D3 energy	-703.9978308194

H 2.018800 -1.131700 -0.749300
C 2.355800 -0.711500 0.212900
C 2.408900 0.822700 0.045600
C 3.754900 -1.285100 0.495200
H 3.700500 -2.379600 0.572400
C 1.007000 1.388700 -0.223200
H 0.649700 1.026600 -1.199800
H 1.058700 2.483700 -0.308100
C 1.339900 -1.104100 1.298900
H 1.719600 -0.751200 2.270000
C -0.071700 -0.521300 1.079700
H -0.634400 -0.695000 2.007700
C -0.008600 1.011800 0.872500
H 0.373200 1.436700 1.812700
C -0.841200 -1.244900 -0.048000
H -1.060700 -2.263900 0.299900
H -0.200600 -1.367400 -0.929700
C -1.416000 1.621800 0.596500
H -1.666000 2.362400 1.367400
H -1.387400 2.177800 -0.352300
C -2.553400 0.589400 0.500700
C -2.164400 -0.543900 -0.472200
H -2.002700 -0.063500 -1.451200
H 1.280900 -2.199600 1.374100
C -3.314400 -1.553300 -0.643900
H -3.027200 -2.320700 -1.375800
C -3.875700 1.253000 0.077100
H -4.153500 2.027100 0.805500
H -3.720300 1.772200 -0.881400
C -4.628300 -0.879300 -1.063400
H -5.431600 -1.624200 -1.132900
H -4.511600 -0.451200 -2.070200
C -5.015700 0.237000 -0.082800
H -5.249300 -0.206100 0.896700
H -5.929200 0.743000 -0.421200
C 3.424200 1.224800 -1.037400
H 3.474200 2.319700 -1.113200
H 3.067600 0.863200 -2.014400
C 4.771400 -0.878200 -0.582400
H 4.490000 -1.344200 -1.538500
H 5.767500 -1.264200 -0.329700
C 4.820200 0.647100 -0.760600
H 5.509600 0.913600 -1.572200
H 5.225100 1.104600 0.154400
H -3.466100 -2.079500 0.311300
H -2.713700 0.143300 1.495600
H 2.761100 1.243100 1.004300
H 4.100200 -0.922800 1.476100

Transition state for enantiomerisation

DFT energy	-704.008137542
gCP correction	0.1252677891
D3 correction	-0.09747564
gCP-D3 correction	0.0277921491
DFT-gCP-D3 energy	-703.9803453929

H -3.0411 -0.6851 1.4040
C -2.6114 -0.7291 0.3899
C -2.4882 0.7357 -0.0707
C -3.6451 -1.4639 -0.4796
H -3.7398 -2.5096 -0.1567

C -1.2896 1.3939 0.6230
H -1.3311 1.2150 1.7085
H -1.3075 2.4842 0.4849
C -1.2393 -1.4522 0.5276
H -1.2988 -2.4455 0.0631
C 0.0432 -0.7480 -0.0106
H 0.1620 -1.0414 -1.0616
C -0.0001 0.8288 0.0092
H -0.0348 1.1372 -1.0469
C 1.2515 -1.3240 0.7701
H 1.3058 -2.4065 0.5897
H 1.0263 -1.2190 1.8399
C 1.2959 1.4556 0.5582
H 1.3002 2.5302 0.3287
H 1.3511 1.3707 1.6541
C 2.4915 0.7472 -0.0904
C 2.6319 -0.6689 0.5040
H 3.1101 -0.5428 1.4892
H -1.0795 -1.6474 1.5957
C 3.6163 -1.4879 -0.3459
H 3.7238 -2.5021 0.0625
C 3.8441 1.4700 -0.0356
H 3.7530 2.4865 -0.4423
H 4.1663 1.5782 1.0112
C 4.9904 -0.7929 -0.3859
H 5.6769 -1.3407 -1.0445
H 5.4303 -0.8332 0.6216
C 4.8979 0.6780 -0.8324
H 4.6282 0.7124 -1.8984
H 5.8820 1.1568 -0.7473
C -3.8388 1.4473 0.0957
H -3.7572 2.4965 -0.2197
H -4.1198 1.4611 1.1598
C -5.0149 -0.7671 -0.3995
H -5.4105 -0.8898 0.6197
H -5.7326 -1.2563 -1.0707
C -4.9289 0.7347 -0.7251
H -5.9042 1.2108 -0.5605
H -4.7008 0.8581 -1.7942
H 3.2212 -1.6012 -1.3670
H 2.2445 0.6366 -1.1608
H -2.2660 0.7333 -1.1521
H -3.2954 -1.4897 -1.5231

C_s intermediate in the enantiomerisation

DFT energy	-704.008199836
gCP correction	0.1253035326
D3 correction	-0.09754987
gCP-D3 correction	0.0277536626
DFT-gCP-D3 energy	-703.9804461734

H -3.0411 -0.6851 1.4040
C -2.6114 -0.7291 0.3899
C -2.4882 0.7357 -0.0707
C -3.6451 -1.4639 -0.4796
H -3.7398 -2.5096 -0.1567
C -1.2896 1.3939 0.6230
H -1.3311 1.2150 1.7085
H -1.3075 2.4842 0.4849
C -1.2393 -1.4522 0.5276
H -1.2988 -2.4455 0.0631
C 0.0432 -0.7480 -0.0106
H 0.1620 -1.0414 -1.0616
C -0.0001 0.8288 0.0092
H -0.0348 1.1372 -1.0469
C 1.2515 -1.3240 0.7701
H 1.3058 -2.4065 0.5897
H 1.0263 -1.2190 1.8399
C 1.2959 1.4556 0.5582
H 1.3002 2.5302 0.3287
H 1.3511 1.3707 1.6541
C 2.4915 0.7472 -0.0904
C 2.6319 -0.6689 0.5040
H 3.1101 -0.5428 1.4892
H -1.0795 -1.6474 1.5957
C 3.6163 -1.4879 -0.3459
H 3.7238 -2.5021 0.0625
C 3.8441 1.4700 -0.0356
H 3.7530 2.4865 -0.4423
H 4.1663 1.5782 1.0112
C 4.9904 -0.7929 -0.3859
H 5.6769 -1.3407 -1.0445
H 5.4303 -0.8332 0.6216
C 4.8979 0.6780 -0.8324
H 4.6282 0.7124 -1.8984
H 5.8820 1.1568 -0.7473
C -3.8388 1.4473 0.0957
H -3.7572 2.4965 -0.2197
H -4.1198 1.4611 1.1598
C -5.0149 -0.7671 -0.3995
H -5.4105 -0.8898 0.6197
H -5.7326 -1.2563 -1.0707
C -4.9289 0.7347 -0.7251
H -5.9042 1.2108 -0.5605
H -4.7008 0.8581 -1.7942
H 3.2212 -1.6012 -1.3670
H 2.2445 0.6366 -1.1608
H -2.2660 0.7333 -1.1521
H -3.2954 -1.4897 -1.5231

Perhydrotetracene 15

Ground state (CCCC)

DFT energy	-704.023874505
gCP correction	0.1267863419
D3 correction	-0.10200354
gCP-D3 correction	0.0247828019
DFT-gCP-D3 energy	-703.9990917031

H -0.658100 2.038200 1.593300
C -0.254500 2.418300 0.643500
C 0.254500 4.671400 -0.496100
C 2.004700 2.836600 -0.499300
C 1.755500 4.351700 -0.452500
C 1.262800 2.099100 0.635500
C -0.499700 3.942300 0.627900
H -0.151100 4.378100 -1.474500
H 1.690300 2.453300 -1.479500
H 2.184400 4.760000 0.475100
H 1.678800 2.488800 1.578400
H -0.170400 4.357700 1.591900
H 0.092800 5.753600 -0.409400
H 3.080400 2.627600 -0.420900
H 2.276700 4.848100 -1.281300
H -1.577200 4.144400 0.556800
C 1.499200 0.574900 0.624300
H 1.187800 0.170000 1.596200
H 2.576300 0.369400 0.542500
C -0.996300 1.685100 -0.493300
H -2.073600 1.888400 -0.410200
H -0.686100 2.095400 -1.463600
C -0.757800 0.160200 -0.512300
H -1.167700 -0.216400 -1.461000
C 0.757800 -0.160200 -0.512300
H 1.167700 0.216400 -1.461000
C -1.499200 -0.574900 0.624300
H -2.576300 -0.369400 0.542500
H -1.187800 -0.170000 1.596200
C 0.996300 -1.685100 -0.493300
H 0.686100 -2.095400 -1.463600
H 2.073600 -1.888400 -0.410200
C 0.254500 -2.418300 0.643500
H 0.658100 -2.038200 1.593300
C -1.262800 -2.099100 0.635500
H -1.678800 -2.488800 1.578400
C 0.499700 -3.942300 0.627900
H 1.577200 -4.144400 0.556800
H 0.170400 -4.357700 1.591900
C -2.004700 -2.836600 -0.499300
H -1.690300 -2.453300 -1.479500
H -3.080400 -2.627600 -0.420900
C -0.254500 -4.671400 -0.496100
H 0.151100 -4.378100 -1.474500
H -0.092800 -5.753600 -0.409400
C -1.755500 -4.351700 -0.452500
H -2.184400 -4.760000 0.475100
H -2.276700 -4.848100 -1.281300

C’TCC intermediate

DFT energy	-704.013517795
gCP correction	0.1268115320
D3 correction	-0.10203819
gCP-D3 correction	0.024773342
DFT-gCP-D3 energy	-703.988744453

H -1.575900 -1.526800 1.083000
C -2.123300 -0.956000 0.318700
C -4.489700 -0.832000 -0.690300
C -3.327400 1.295000 0.051900
C -4.673000 0.566200 -0.083300
C -2.312100 0.478100 0.878700
C -3.484000 -1.660000 0.125200
H -4.143600 -0.734700 -1.728900
H -2.931700 1.500500 -0.951900
H -5.134000 0.470300 0.911400
H -2.746800 0.375600 1.885900
H -3.919200 -1.855200 1.116600
H -5.452400 -1.357400 -0.735500
H -3.474000 2.274000 0.528400
H -5.367000 1.160300 -0.692100
H -3.330500 -2.641900 -0.343000
C -0.950700 1.188700 1.034400
H -0.392600 0.705800 1.847700
H -1.114100 2.227700 1.354500
C -1.260700 -0.964400 -0.960000
H -1.094700 -2.004900 -1.274600
H -1.805700 -0.483100 -1.782700
C 0.099100 -0.252400 -0.787600
H 0.541200 -0.156400 -1.789700
C -0.103800 1.183100 -0.255900
H -0.687800 1.721400 -1.015300
C 1.087200 -1.085200 0.073200
H 1.603300 -1.786100 -0.594600
H 0.537500 -1.716600 0.782200
C 1.245300 1.941400 -0.109500
H 1.433900 2.511100 -1.029200
H 1.134900 2.692300 0.684000
C 2.496500 1.066600 0.186400
H 3.128900 1.626500 0.892300
C 2.119700 -0.260300 0.881900
H 1.651000 0.005000 1.839500

C 3.376500 0.813500 -1.056100
H 2.806300 0.270400 -1.823300
H 3.653800 1.776500 -1.506300
C 3.382400 -1.084400 1.214200
H 3.092400 -2.049700 1.651700
H 3.954100 -0.553400 1.989500
C 4.641100 0.018000 -0.696500
H 5.243000 -0.168100 -1.595600
H 5.265400 0.625900 -0.024400
C 4.295300 -1.307800 -0.002700
H 5.211900 -1.824000 0.310700
H 3.802700 -1.973800 -0.724900

Concerted inversion TS

DFT energy	-703.996906687
gCP correction	0.1263223580
D3 correction	-0.10067168
gCP-D3 correction	0.025650678
DFT-gCP-D3 energy	-703.971256009

H -0.612700 -2.010000 2.763800
C -0.584300 -0.922700 2.605600
C -1.020300 1.226300 3.941400
C 1.175300 0.951700 2.722000
C 0.486500 1.517400 3.972700
C 0.915800 -0.560400 2.555400
C -1.281400 -0.282000 3.824300
H -1.472700 1.752300 3.087500
H 0.804000 1.502700 1.847800
H 1.392000 -1.057200 3.415500
H -0.909300 -0.770000 4.737500
H 2.257900 1.135200 2.764100
H 0.667800 2.597400 4.047700
H -2.360300 -0.484700 3.784400
C 1.513700 -1.155100 1.271100
H 1.274600 -2.229300 1.239800
H 2.610700 -1.084600 1.288300
C -1.341900 -0.659000 1.265900
H -2.144000 0.070800 1.437800
C -0.552000 -0.183300 0.000000
H -0.629900 0.910900 0.000000
C 0.997400 -0.461200 0.000000
H 1.470600 0.528200 0.000000
C -1.341900 -0.659000 -1.265900
H -2.144000 0.070800 -1.437800
H -1.862400 -1.590400 -1.012400
C 1.513700 -1.155100 -1.271100
H 2.610700 -1.084600 -1.288300
H 1.274600 -2.229300 -1.239800
C 0.915800 -0.560400 -2.555400
H 1.392000 -1.057200 -3.415500
C -0.584300 -0.922700 -2.605600
H -0.612700 -2.010000 -2.763800
H -1.862400 -1.590400 1.012400
C 1.175300 0.951700 -2.722000
H 0.804000 1.502700 -1.847800
H 2.257900 1.135200 -2.764100
C -1.281400 -0.282000 -3.824300
H -2.360300 -0.484700 -3.784400
H -0.909300 -0.770000 -4.737500
C -1.020300 1.226300 -3.941400
H -1.472700 1.752300 -3.087500
H -1.508000 1.622200 -4.841500
C 0.486500 1.517400 -3.972700
H 0.925100 1.062800 -4.873700
H 0.667800 2.597400 -4.047700
H -1.508000 1.622200 4.841500
H 0.925100 1.062800 4.873700

Transition state for stepwise conversion of TTCC to C’TCC

DFT energy	-703.998673740
gCP correction	0.1262699257
D3 correction	-0.10070322
gCP-D3 correction	0.0255667057
SCF-gCP-D3 energy	-703.9731070343

C 4.612000 -0.658600 -0.469200
H 5.427200 -1.217500 0.008200
C 4.583100 0.788300 0.046800
H 4.508200 0.781700 1.143000
C 3.269000 -1.367700 -0.233900
H 3.112500 -1.498000 0.845400
H 3.296900 -2.379000 -0.662100
C 3.406700 1.575200 -0.555600
H 3.373800 2.589000 -0.133600
H 3.582400 1.694400 -1.634900
C 2.043900 0.878700 -0.355200
H 1.304300 1.413300 -0.972000
C 2.098500 -0.584800 -0.862600
H 2.301700 -0.545600 -1.944800
C 0.728700 -1.264600 -0.683600
H 0.800000 -2.325100 -0.966200
H 0.039100 -0.809000 -1.402900
C 0.139500 -1.169400 0.741600
H 0.741900 -1.827100 1.384400
C 1.559200 0.940600 1.104600
H 2.308100 0.469700 1.754900
H 1.503300 1.989900 1.427200
C 0.201400 0.252400 1.363800

H 0.149200 0.098500 2.450700
C -1.318200 -1.659100 0.799300
H -1.379200 -2.723000 0.531700
H -1.664200 -1.587300 1.841500
C -1.048400 1.116100 1.009700
H -0.745200 2.158700 0.842600
H -1.705500 1.143800 1.890200
C -1.911700 0.662500 -0.190200
H -1.297600 0.787500 -1.090000
C -2.272700 -0.859800 -0.117300
H -2.145000 -1.257300 -1.134500
C -3.743700 -1.121000 0.231600
H -3.971400 -0.761600 1.247200
H -3.927600 -2.204000 0.235800
C -3.117000 1.656200 -0.333400
H -3.183800 2.226600 0.600900
H -2.847600 2.397000 -1.095100
C -4.645500 -0.421200 -0.786200
H -5.692300 -0.729300 -0.669800
H -4.341300 -0.733800 -1.795900
C -4.543300 1.104700 -0.642600
H -5.210100 1.407000 0.175200
H -4.943800 1.585500 -1.543200
H 4.829100 -0.651200 -1.548000
H 5.527300 1.295000 -0.191300

Perhydrochrysene 16

Lowest energy CCCC conformer

DFT energy	-704.023719803
gCP correction	0.1269630309
D3 correction	-0.10256590
gCP-D3 correction	0.0243971309
DFT-gCP-D3 energy	-703.9993226721

H -1.199200 -0.032100 1.098300
C -0.724400 0.281900 0.155900
C 0.138400 2.433500 -0.978900
C 1.569800 0.333000 -0.978200
C 1.569800 1.866900 -0.924100
C 0.724400 -0.281900 0.155900
C -0.755400 1.833500 0.139300
H -0.283500 2.118500 -1.945000
H 2.079900 2.195200 -0.008300
H 1.199200 0.032100 1.098300
H 2.598600 -0.045400 -0.906300
H 2.153800 2.273500 -1.761100
H -1.792800 2.131000 -0.086500
C -0.426100 2.441900 1.519400
H 0.576700 2.134800 1.846000
H -1.125300 2.038800 2.264700
C 0.096700 3.976400 -0.951300
H -0.911100 4.303900 -1.246800
H 0.785600 4.377200 -1.707500
C -0.499300 3.976300 1.508100
H -1.536600 4.287300 1.312100
H -0.236300 4.375100 2.496500
C 0.415900 4.571300 0.428800
H 1.464700 4.373300 0.690800
H 0.308700 5.663200 0.397000
C -1.569800 -0.333000 -0.978200
H -1.193400 -0.008500 -1.957900
H -2.598600 0.045400 -0.906300
C -0.138400 -2.433500 -0.978900
C -1.569800 -1.866900 -0.924100
H -2.079900 -2.195200 -0.008300
H -2.153800 -2.273500 -1.761100
H 1.193400 0.008500 -1.957900
H 0.283500 -2.118500 -1.945000
C 0.755400 -1.833500 0.139300
H 1.792800 -2.131000 -0.086500
C 0.426100 -2.441900 1.519400
H -0.576700 -2.134800 1.846000
H 1.125300 -2.038800 2.264700
C -0.096700 -3.976400 -0.951300
H -0.785600 -4.377200 -1.707500
H 0.911100 -4.303900 -1.246800
C 0.499300 -3.976300 1.508100
H 0.236300 -4.375100 2.496500
H 1.536600 -4.287300 1.312100
C -0.415900 -4.571300 0.428800
H -0.308700 -5.663200 0.397000
H -1.464700 -4.373300 0.690800

Less stable CCCC conformer

DFT energy	-704.017968199
gCP correction	0.1280428482
D3 correction	-0.10503408
gCP-D3 correction	0.0230087682
DFT-gCP-D3 energy	-703.9949594308

C -0.304000 1.577900 0.640200
C -2.423300 0.152500 0.576500
C -0.282900 -0.727300 -0.525100
C -1.824700 -0.679800 -0.566200
C 0.282900 0.727300 -0.525100
C -1.859500 1.585100 0.621200
H -2.218400 -0.342400 1.535900
H -2.124900 -0.241700 -1.527700

H -0.046600 1.187200 -1.467400
H -0.002000 1.103800 1.584100
H -3.517200 0.187200 0.479700
H 0.046600 -1.187200 -1.467400
H -2.250800 -1.689600 -0.557200
H -2.184700 2.041100 1.569800
C 0.211000 3.035400 0.695900
H -0.106100 3.465200 1.657800
H 1.306500 3.063700 0.703000
C -2.423300 2.479800 -0.504200
H -3.519700 2.492700 -0.435600
H -2.184800 2.056500 -1.489000
C -1.874600 3.912800 -0.433200
H -2.237000 4.392900 0.488200
H -2.262800 4.509600 -1.268900
C -0.339600 3.922600 -0.431100
H 0.033300 4.948200 -0.312000
H 0.030800 3.571700 -1.404800
C 1.824700 0.679800 -0.566200
H 2.250800 1.689600 -0.557200
H 2.124900 0.241700 -1.527700
C 0.304000 -1.577900 0.640200
H 0.002000 -1.103800 1.584100
C 1.859500 -1.585100 0.621200
H 2.184700 -2.041100 1.569800
C 2.423300 -0.152500 0.576500
H 2.218400 0.342400 1.535900
H 3.517200 -0.187200 0.479700
C 2.423300 -2.479800 -0.504200
H 2.184800 -2.056500 -1.489000
H 3.519700 -2.492700 -0.435600
C -0.211000 -3.035400 0.695900
H -1.306500 -3.063700 0.703000
H 0.106100 -3.465200 1.657800
C 0.339600 -3.922600 -0.431100
H -0.030800 -3.571700 -1.404800
H -0.033300 -4.948200 -0.312000
C 1.874600 -3.912800 -0.433200
H 2.237000 -4.392900 0.488200
H 2.262800 -4.509600 -1.268900

Transition state for pseudorotation of CTTC’

DFT energy	-704.002530875
gCP correction	0.1271532616
D3 correction	-0.10295391
gCP-D3 correction	0.0241993516
DFT-gCP-D3 energy	-703.9783315234

C 0.260600 0.978600 0.292500
H 0.037300 1.458000 1.257700
C -0.517100 -0.399600 0.262000
H -0.916200 -0.562200 1.270600
C -1.734900 -0.361900 -0.709800
H -1.333600 -0.347600 -1.733600
C -0.257500 1.951100 -0.785100
H -0.059200 1.556300 -1.790200
H 0.296800 2.896500 -0.716000
C -2.600300 0.919600 -0.538300
H -3.312900 0.918600 -1.376500
C -1.754800 2.222700 -0.615800
H -1.898000 2.802500 0.307000
H -2.112400 2.862600 -1.432100
C 0.428700 -1.593400 -0.023800
H 0.952600 -1.856400 0.901000
H -0.155200 -2.484300 -0.278900
C 1.804100 0.843000 0.216000
H 2.193200 1.858200 0.035900
C 1.466400 -1.348900 -1.129900
H 0.968000 -1.383300 -2.107500
H 2.170100 -2.191900 -1.132300
C 2.242300 -0.002400 -1.009100
H 1.986400 0.593000 -1.894600
C -2.660600 -1.599900 -0.618600
H -2.090400 -2.528300 -0.734300
H -3.348400 -1.565200 -1.476600
C -3.441600 0.874100 0.754300
H -2.773300 0.908000 1.627500
C -3.498200 -1.654100 0.668100
H -2.842300 -1.783600 1.540500
H -4.154100 -2.534100 0.645300
C -4.328500 -0.374900 0.837900
H -4.875300 -0.392400 1.789700
C 2.448400 0.399300 1.549900
H 2.205000 1.145100 2.319500
H 2.021500 -0.546600 1.902700
C 3.774600 -0.186000 -1.040700
H 4.046100 -0.857800 -1.866300
C 4.345400 -0.704000 0.286500
H 3.954300 -1.710600 0.496400
C 3.971800 0.239900 1.437500
H 4.430900 1.223400 1.255800
H 4.383300 -0.125500 2.387300
H -5.087600 -0.330900 0.042400
H -4.059300 1.780800 0.810600
H 4.244100 0.783700 -1.264200
H 5.435800 -0.805500 0.212100

Rate limiting chair-to-twist TS (CCTC’ to CTTC’)

DFT energy	-703.999309885

gCP correction 0.1269930253
D3 correction -0.10268525
gCP-D3 correction 0.0243077753
DFT-gCP-D3 energy -703.9750021097

C 0.179000 0.848500 0.237300
H -0.205100 1.158300 1.221500
C -0.504600 -0.509400 -0.112200
H -0.717900 -0.991700 0.848600
C -1.868800 -0.300700 -0.842900
H -1.635900 -0.179500 -1.911400
C -0.259500 1.946600 -0.747000
H -0.060600 1.634200 -1.780700
H 0.327000 2.861100 -0.585900
C -2.631200 0.976600 -0.409500
H -3.489500 1.065600 -1.092500
C -1.756400 2.254800 -0.569700
H -1.882800 2.889000 0.318500
H -2.110600 2.851400 -1.419600
C 0.381000 -1.521600 -0.919000
H 0.488600 -2.425100 -0.308200
H -0.184000 -1.846700 -1.799900
C 1.708800 0.743000 0.387800
H 2.078700 1.764700 0.575100
C 1.806800 -1.104300 -1.398900
H 1.832900 -1.104400 -2.495400
H 2.499000 -1.899600 -1.100200
C 2.368600 0.257600 -0.917200
H 2.137900 1.005100 -1.687600
C -2.796800 -1.531300 -0.718000
H -2.254300 -2.447500 -0.984800
H -3.606400 -1.430300 -1.455700
C -3.230000 0.828900 1.004800
H -2.425200 0.743100 1.749100
C -3.425000 -1.678600 0.677700
H -2.647200 -1.915400 1.417400
H -4.118700 -2.529300 0.682300
C -4.152400 -0.395200 1.103900
H -4.543800 -0.498000 2.124400
C 2.152500 -0.105400 1.595800
H 1.680400 0.280500 2.509900
H 1.806200 -1.142600 1.484000
C 3.905600 0.221500 -0.766600
H 4.361500 -0.186400 -1.679400
C 4.372100 -0.579000 0.461100
H 4.155000 -1.646900 0.317200
C 3.681900 -0.097200 1.746300
H 4.014100 0.926800 1.974200
H 3.986000 -0.720000 2.597500
H 4.271600 1.254500 -0.673000
H 5.462500 -0.501600 0.561600
H -3.786000 1.741300 1.260300
H -5.023400 -0.240200 0.449600

Transition state for concerted CCTC’ to CTC’C’

DFT energy -703.993403499
gCP correction 0.1265057222
D3 correction -0.10095106
gCP-D3 correction 0.0255546622
DFT-gCP-D3 energy -703.9678488368

H 1.207400 -1.334600 -2.366000
C 1.592500 -1.742200 -1.419700
C 2.610100 -0.752600 -0.848100
H 3.410600 -0.621700 -1.594000
C 1.927400 0.622100 -0.696800
H 1.671000 0.932000 -1.720700
C 0.437500 -2.000000 -0.448900
H 0.813000 -2.649800 0.351300
C -0.228400 -0.740400 0.209200
H -0.189300 -0.959900 1.281100
C 0.590600 0.631600 0.118100
H 0.868900 0.834400 1.159400
C -1.778800 -0.694700 -0.094300
H -2.006400 -1.513200 -0.792000
C -0.218000 1.879500 -0.339000
H 0.224800 2.773000 0.118000
H -0.099500 2.006800 -1.424900
C -1.718200 1.828200 -0.058200
H -2.192300 2.744800 -0.435400
C -2.293200 0.597600 -0.768300
H -1.886900 0.620700 -1.792000
H -0.328300 -2.592600 -0.963200
H 2.075300 -2.697900 -1.666600
H -1.917000 1.797700 1.021700
C -2.566300 -1.003900 1.202900
H -2.307000 -0.262100 1.972800
H -2.233100 -1.974200 1.595400
C -3.829600 0.599000 -0.907700
H -4.116100 -0.171800 -1.639000
H -4.152800 1.559800 -1.330900
C -4.567700 0.315500 0.406400
H -4.387200 1.129500 1.123000
H -5.651100 0.290500 0.231400
C -4.087800 -1.010700 1.008500
H -4.589100 -1.207700 1.965100
H -4.366200 -1.834800 0.334300
C 2.944200 1.654500 -0.150900
H 2.461100 2.625200 0.015300
H 3.707700 1.823600 -0.924500

C 3.651300 1.190200 1.134000
H 2.929700 1.133700 1.961600
H 4.399800 1.935500 1.432800
C 3.293700 -1.221900 0.450000
H 2.546500 -1.393500 1.237400
H 3.788200 -2.188200 0.278700
C 4.310900 -0.183700 0.947500
H 4.769500 -0.518200 1.887100
H 5.127000 -0.096600 0.214600

Helicenes

Helicene 12

Helicene12GS.log

DFT energy	-1922.24557427
gCP correction	0.1520396748
D3 correction	-0.27647961
gCP-D3 correction	-0.1244399352
DFT-gCP-D3 energy	-1922.3700142052

C 0.000000 0.000000 1.221000
C 0.743900 -2.305700 2.669900
C 0.000000 0.000000 2.643300
C 0.000000 -1.286000 0.551600
C 0.543000 -2.394800 1.259600
C 0.377700 -1.180400 3.350000
H 0.423700 -1.143600 4.435500
H 1.130000 -3.175400 3.195800
C 0.000000 1.286000 0.551600
C -0.641200 3.691400 -0.784800
C 0.567100 1.539100 -0.762100
C -0.543000 2.394800 1.259600
C -0.856800 3.597800 0.559300
C 0.084200 2.673800 -1.474900
H -1.315900 4.415000 1.110000
H -0.962600 4.567700 -1.342500
C -0.743900 2.305700 2.669900
C -0.377700 1.180400 3.350000
H -1.130000 3.175400 3.195800
H -0.423700 1.143600 4.435500
C 1.616800 0.749200 -1.390700
C 3.157600 -1.138300 -2.836400
C 2.633000 -0.025200 -0.695700
C 1.693900 0.790700 -2.814600
C 2.461500 -0.182800 -3.517000
C 3.296100 -1.052800 -1.420400
H 2.452300 -0.164800 -4.604000
H 3.689500 -1.925300 -3.365400
C 1.027200 1.824900 -3.536100
C 0.335700 2.796000 -2.873200
H 1.140200 1.857900 -4.616900
H -0.082800 3.647200 -3.404300
C -0.567100 -1.539100 -0.762100
C -1.027200 -1.824900 -3.536100
C -0.084200 -2.673800 -1.474900
C -1.616800 -0.749200 -1.390700
C -1.693900 -0.790700 -2.814600
C -0.335700 -2.796000 -2.873200
H 0.082800 -3.647200 -3.404300
H -1.140200 -1.857900 -4.616900
C 3.137100 0.268800 0.641900
C 4.223600 0.796800 3.218600
C 2.926400 1.508100 1.299500
C 4.010300 -0.666200 1.284200
C 4.503700 -0.394400 2.583400
C 3.453300 1.767700 2.549700
H 2.366300 2.285800 0.798500
H 5.143500 -1.135100 3.058000
H 3.276300 2.734400 3.013400
H 4.621700 0.999800 4.209200
C 0.641200 -3.691400 -0.784800
C 0.856800 -3.597800 0.559300
H 0.962600 -4.567700 -1.342500
H 1.315900 -4.415000 1.110000
C 4.160200 -1.972500 -0.744900
C 4.455500 -1.824900 0.575100
H 4.580700 -2.794300 -1.319500
H 5.099800 -2.536100 1.086400
C -2.461500 0.182800 -3.517000
C -3.157600 1.138300 -2.836400
H -2.452300 0.164800 -4.604000
C -2.633000 0.025200 -0.695700
C -3.296100 1.052800 -1.420400
C -4.160200 1.972500 -0.744900
C -4.455500 1.824900 0.575100
H -4.580700 2.794300 -1.319500
H -5.099800 2.536100 1.086400
H -3.689500 1.925300 -3.365400
C -4.010300 0.666200 1.284200
C -3.137100 -0.268800 0.641900
C -4.503700 0.394400 2.583400
C -4.223600 -0.796800 3.218600
H -5.143500 1.135100 3.058000
H -4.621700 -0.999800 4.209200
C -2.926400 -1.508100 1.299500
C -3.453300 -1.767700 2.549700
H -2.366300 -2.285800 0.798500
H -3.276300 -2.734400 3.013400

helicene12TSb.log

DFT energy	-1922.15288472
gCP correction	0.1518054955
D3 correction	-0.26286380
gCP-D3 correction	-0.1110583045
DFT-gCP-D3 energy	-1922.2639430245

C 2.642900 1.099600 2.214500
C 2.762000 2.518300 2.222100
H 3.293900 2.993800 3.042600
C 2.246300 3.244800 1.194500
H 2.339400 4.326900 1.165900
C 1.747200 0.459600 1.300400
C 1.590900 1.146800 0.000000
C 1.884500 2.548500 0.000000
C 1.747200 0.459600 -1.300400
C 2.246300 3.244800 -1.194500
H 2.339400 4.326900 -1.165900
C 2.762000 2.518300 -2.222100
H 3.293900 2.993800 -3.042600
C 2.642900 1.099600 -2.214500
C 1.311600 -0.867600 -1.735000
C 3.458400 0.322300 -3.090700
H 4.182100 0.839400 -3.715600
C 3.411300 -1.040000 -3.033100
H 4.126600 -1.659200 -3.567600
C 2.304000 -1.665800 -2.387900
C 1.311600 -0.867600 1.735000
C 3.458400 0.322300 3.090700
H 4.182100 0.839400 3.715600
C 3.411300 -1.040000 3.033100
H 4.126600 -1.659200 3.567600
C 2.304000 -1.665800 2.387900
C -0.047300 -1.399100 1.759700
C 2.125800 -3.074800 2.496100
H 2.945300 -3.673200 2.886300
C 0.945900 -3.646700 2.124800
H 0.809100 -4.724500 2.158700
C -0.183200 -2.824200 1.843100
C -0.047300 -1.399100 -1.759700
C 2.125800 -3.074800 -2.496100
H 2.945300 -3.673200 -2.886300
C 0.945900 -3.646700 -2.124800
H 0.809100 -4.724500 -2.158700
C -0.183200 -2.824200 -1.843100
C -1.460800 -3.439200 -1.747300
H -1.515400 -4.524600 -1.776200
C -1.267900 -0.614300 -1.907100
C -2.517700 -1.268100 -1.710000
C -2.586700 -2.684300 -1.611700
H -3.561200 -3.151000 -1.491300
C -1.267900 -0.614300 1.907100
C -1.460800 -3.439200 1.747300
C -2.586700 -2.684300 1.611700
C -2.517700 -1.268100 1.710000
H -1.515400 -4.524600 1.776200
H -3.561200 -3.151000 1.491300
C -3.735600 -0.517200 1.659700
H -4.653600 -1.054400 1.434600
C -1.346700 0.747300 2.439300
C -2.569400 1.484200 2.323700
C -3.750700 0.826100 1.864100
H -4.672500 1.396700 1.782800
C -3.735600 -0.517200 -1.659700
H -4.653600 -1.054400 -1.434600
C -1.346700 0.747300 -2.439300
C -2.569400 1.484200 -2.323700
C -3.750700 0.826100 -1.864100
H -4.672500 1.396700 -1.782800
C -0.340400 1.324000 3.251700
C -0.458000 2.600200 3.769600
C -2.645800 2.812800 2.807100
C -1.599000 3.379400 3.502900
H -3.575000 3.360300 2.665600
H -1.676100 4.392600 3.888300
C -0.340400 1.324000 -3.251700
C -0.458000 2.600200 -3.769600
C -1.599000 3.379400 -3.502900
C -2.645800 2.812800 -2.807100
H -1.676100 4.392600 -3.888300
H -3.575000 3.360300 -2.665600
H 0.519800 0.731300 -3.522800
H 0.334900 2.995400 -4.398500
H 0.519800 0.731300 3.522800
H 0.334900 2.995400 4.398500

Helicene 20

Helicene20GS.log

DFT energy	-3151.30823529
gCP correction	0.2481432786
D3 correction	-0.49321451
gCP-D3 correction	-0.2450712314
DFT-gCP-D3 energy	-3151.5533065214

C -1.168800 -0.548600 0.912200
C -2.972000 -2.242600 -0.449600
C -1.920400 -1.531200 1.615400
C -1.641100 -0.170000 -0.408500
C -2.394100 -1.133400 -1.136400
C -2.829000 -2.369000 0.901900

H -3.356000 -3.147000 1.448800
H -3.582300 -2.942400 -1.015000
C 0.000000 0.000000 1.578100
C 1.760100 1.665700 3.027000
C 1.168800 0.548600 0.912200
C 0.000000 0.000000 3.001300
C 0.907100 0.845800 3.707300
C 1.920400 1.531200 1.615400
H 0.857800 0.867700 4.793200
H 2.378400 2.389000 3.552900
C -0.907100 -0.845800 3.707300
C -1.760100 -1.665700 3.027000
H -0.857800 -0.867700 4.793200
H -2.378400 -2.389000 3.552900
C 1.641100 0.170000 -0.408500
C 2.024300 -0.091200 -3.196400
C 1.428200 -1.120500 -1.039200
C 2.394100 1.133400 -1.136400
C 2.573900 0.973700 -2.542900
C 1.450800 -1.171200 -2.461100
H 3.109100 1.747200 -3.088100
H 2.072700 -0.167900 -4.279700
C 2.972000 2.242600 -0.449600
C 2.829000 2.369000 0.901900
H 3.582300 2.942400 -1.015000
H 3.356000 3.147000 1.448800
C -1.428200 1.120500 -1.039200
C -0.362100 3.337700 -2.430800
C -1.450800 1.171200 -2.461100
C -1.225900 2.373800 -0.335000
C -0.519100 3.405300 -1.014100
C -0.910800 2.306600 -3.136700
H -0.895700 2.304200 -4.223700
H 0.138800 4.158600 -2.938000
C 1.225900 -2.373800 -0.335000
C 0.178600 -4.589200 1.073700
C 1.741500 -2.684400 0.985900
C 0.519100 -3.405300 -1.014100
C 0.000000 -4.511100 -0.277300
C 1.071800 -3.693800 1.734300
H -0.582900 -5.259800 -0.808000
H -0.294000 -5.376300 1.656400
C -2.024300 0.091200 -3.196400
C -2.573900 -0.973700 -2.542900
H -2.072700 0.167900 -4.279700
H -3.109100 -1.747200 -3.088100
C 0.910800 -2.306600 -3.136700
C 0.362100 -3.337700 -2.430800
H 0.895700 -2.304200 -4.223700
H -0.138800 -4.158600 -2.938000
C 0.000000 4.511100 -0.277300
C -0.178600 4.589200 1.073700
H 0.582900 5.259800 -0.808000
H 0.294000 5.376300 1.656400
C -1.741500 2.684400 0.985900
C -1.071800 3.693800 1.734300
C -2.948100 2.118200 1.569000
C -4.871000 0.535300 2.909100
C -3.065700 2.146800 2.988800
C -4.081800 1.593300 0.819300
C -4.928100 0.659900 1.489300
C -4.041800 1.335500 3.638900
H -4.074300 1.333300 4.725600
H -5.554000 -0.155900 3.396800
C 2.948100 -2.118200 1.569000
C 4.871000 -0.535300 2.909100
C 4.081800 -1.593300 0.819300
C 3.065700 -2.146800 2.988800
C 4.041800 -1.335500 3.638900
C 4.928100 -0.659900 1.489300
H 4.074300 -1.333300 4.725600
H 5.554000 0.155900 3.396800
C 1.332200 -3.832500 3.130600
C 2.224000 -3.009100 3.753900
H 0.769500 -4.574400 3.691900
H 2.360000 -3.045000 4.832100
C -1.332200 3.832500 3.130600
C -2.224000 3.009100 3.753900
H -0.769500 4.574400 3.691900
H -2.360000 3.045000 4.832100
C -4.481200 2.000700 -0.519100
C -5.153400 2.508900 -3.252100
C -4.216400 3.310500 -1.106500
C -5.332500 1.131000 -1.253700
C -5.604300 1.382500 -2.636400
C -4.511400 3.533500 -2.489400
H -6.189800 0.647400 -3.183700
H -5.350300 2.686500 -4.306700
C -5.859000 -0.121600 0.746100
C -5.979400 0.040700 -0.603000
H -6.446700 -0.868600 1.274100
H -6.638300 -0.597700 -1.186600
C 4.481200 -2.000700 -0.519100
C 5.153400 -2.508900 -3.252100
C 5.332500 -1.131000 -1.253700
C 4.216400 -3.310500 -1.106500
C 4.511400 -3.533500 -2.489400
C 5.604300 -1.382500 -2.636400
H 6.189800 -0.647400 -3.183700
H 5.350300 -2.686500 -4.306700
C 5.979400 -0.040700 -0.603000
C 5.859000 0.121600 0.746100
H 6.638300 0.597700 -1.186600
H 6.446700 0.868600 1.274100

C -3.820000 4.439400 -0.344600
C -3.622900 5.677500 -0.925100
H -3.702600 4.341400 0.725800
H -3.329200 6.518100 -0.302400
C -4.259400 4.800000 -3.070500
C -3.809400 5.857800 -2.309200
H -4.467800 4.932400 -4.129900
H -3.636100 6.829600 -2.764000
C 3.820000 -4.439400 -0.344600
C 3.622900 -5.677500 -0.925100
H 3.702600 -4.341400 0.725800
H 3.329200 -6.518100 -0.302400
C 4.259400 -4.800000 -3.070500
C 3.809400 -5.857800 -2.309200
H 4.467800 -4.932400 -4.129900
H 3.636100 -6.829600 -2.764000

helicene20TS.log

DFT energy	-3151.20333385
gCP correction	0.2478149130
D3 correction	-0.47841855
gCP-D3 correction	-0.230603637
DFT-gCP-D3 energy	-3151.433937487

C 3.068500 -0.839800 2.156300
C 4.122000 0.120300 2.178400
H 4.859400 0.069300 2.973900
C 4.205600 1.053200 1.192000
H 5.013700 1.779500 1.171400
C 1.946800 -0.636400 1.298300
C 2.277100 0.003700 0.000000
C 3.446800 0.823200 0.000000
C 1.946800 -0.636400 -1.298300
C 4.205600 1.053200 -1.192000
H 5.013700 1.779500 -1.171400
C 4.122000 0.120300 -2.178400
H 4.859400 0.069300 -2.973900
C 3.068500 -0.839800 -2.156300
C 0.749700 -1.354100 -1.727700
C 3.184100 -2.054800 -2.897700
H 4.098400 -2.226600 -3.459700
C 2.225500 -3.020600 -2.788600
H 2.362600 -4.009900 -3.216700
C 0.951600 -2.666600 -2.253700
C 0.749700 -1.354100 1.727700
C 3.184100 -2.054800 2.897700
H 4.098400 -2.226600 3.459700
C 2.225500 -3.020600 2.788600
H 2.362600 -4.009900 3.216700
C 0.951600 -2.666600 2.253700
C -0.595400 -0.825300 1.825700
C -0.149500 -3.568200 2.330800
H 0.042700 -4.595300 2.631300
C -1.413400 -3.147400 2.031300
H -2.250000 -3.841500 2.034600
C -1.677000 -1.759700 1.845600
C -0.595400 -0.825300 -1.825700
C -0.149500 -3.568200 -2.330800
H 0.042700 -4.595300 -2.631300
C -1.413400 -3.147400 -2.031300
H -2.250000 -3.841500 -2.034600
C -1.677000 -1.759700 -1.845600
C -3.019000 -1.296800 -1.740900
H -3.820800 -2.030900 -1.730700
C -0.924600 0.572000 -2.048900
C -2.251100 0.998300 -1.749100
C -3.288000 0.033600 -1.605700
H -4.303800 0.383900 -1.442800
C -0.924600 0.572000 2.048900
C -3.019000 -1.296800 1.740900
C -3.288000 0.033600 1.605700
C -2.251100 0.998300 1.749100
H -3.820800 -2.030900 1.730700
H -4.303800 0.383900 1.442800
C -2.537500 2.389600 1.619800
H -3.546700 2.685900 1.347000
C -0.058300 1.572300 2.659700
C -0.286400 2.933300 2.303200
C -1.545700 3.315900 1.755100
H -1.723500 4.366900 1.541800
C -2.537500 2.389600 -1.619800
H -3.546700 2.685900 -1.347000
C -0.058300 1.572300 -2.659700
C -0.286400 2.933300 -2.303200
C -1.545700 3.315900 -1.755100
H -1.723500 4.366900 -1.541800
C 0.906300 1.337800 3.726300
C 1.934600 2.310800 3.908200
C 0.705100 3.919100 2.586900
C 1.833700 3.586600 3.277000
H 0.547500 4.932000 2.224300
H 2.627200 4.311800 3.438500
C 0.906300 1.337800 -3.726300
C 1.934600 2.310800 -3.908200
C 1.833700 3.586600 -3.277000
C 0.705100 3.919100 -2.586900
H 2.627200 4.311800 -3.438500
H 0.547500 4.932000 -2.224300
C 0.841300 0.254600 -4.700300
C 1.081100 -2.162200 -6.167100
C -0.359200 -0.461700 -5.109100
C 2.041500 -0.074900 -5.400400

C 2.138300 -1.301800 -6.119300
C -0.200900 -1.739800 -5.710100
H 3.085900 -1.552300 -6.590200
H 1.170700 -3.134100 -6.646300
C 0.841300 0.254600 4.700300
C 1.081100 -2.162200 6.167100
C 2.041500 -0.074900 5.400400
C -0.359200 -0.461700 5.109100
C -0.200900 -1.739800 5.710100
C 2.138300 -1.301800 6.119300
H 3.085900 -1.552300 6.590200
H 1.170700 -3.134100 6.646300
C 3.041900 2.034000 -4.761600
C 3.136800 0.837300 -5.408800
H 3.827300 2.780800 -4.848600
H 4.017900 0.585100 -5.994300
C 3.041900 2.034000 4.761600
C 3.136800 0.837300 5.408800
H 3.827300 2.780800 4.848600
H 4.017900 0.585100 5.994300
C -1.701300 0.109100 5.134300
C -4.333100 1.169600 5.341200
C -1.949200 1.503900 5.066200
C -2.821000 -0.737300 5.415100
C -4.124700 -0.187600 5.468300
C -3.225200 2.022400 5.172100
H -1.115600 2.185500 4.964200
H -4.960400 -0.858200 5.656700
H -3.369300 3.098500 5.128700
H -5.337000 1.581000 5.406800
C -1.701300 0.109100 -5.134300
C -4.333100 1.169600 -5.341200
C -2.821000 -0.737300 -5.415100
C -1.949200 1.503900 -5.066200
C -3.225200 2.022400 -5.172100
C -4.124700 -0.187600 -5.468300
H -1.115600 2.185500 -4.964200
H -3.369300 3.098500 -5.128700
H -4.960400 -0.858200 -5.656700
H -5.337000 1.581000 -5.406800
C -1.341400 -2.571200 -5.949800
C -2.605800 -2.111400 -5.745900
H -1.169800 -3.579900 -6.318200
H -3.468100 -2.749500 -5.924100
C -1.341400 -2.571200 5.949800
C -2.605800 -2.111400 5.745900
H -1.169800 -3.579900 6.318200
H -3.468100 -2.749500 5.924100

Perhydrohelicenes

B3LYP/6-31G* activation energies

Activation energies at B3LYP/6-31G* for perhydrohelicenes with 4 – 12 ring (c.f. Table 1.

No. of Rings	4	6	8	10	12
**CTtoTT TS** for [ag^-g^+a]	75.7	67.9	98.7	91.5	103.3
[ag^-g^+a] **C_s TS**	63.8	64.8	85.3	74.3	86.4
[g^+***aa***g^-] **C_s TS**	66.8	79.8	71.9	86.0	78.5

Perhydro[4]helicene

Ground state

DFT energy -704.019838411
gCP correction 0.1276637133
D3 correction -0.10429890
gCP-D3 correction 0.0233648133
DFT-gCP-D3 energy -703.9964735977

H -1.010600 -1.596900 -0.296300
C -1.167000 -0.586900 0.106800
C 0.081000 1.566300 0.703700
C -2.106700 1.687100 -0.595500
C -0.727200 2.321300 -0.370700
C -1.994200 0.192300 -0.950400
C 0.227700 0.053700 0.363300
H -0.501100 1.636000 1.634300
H -0.180700 2.341400 -1.322900
H -1.451400 0.136200 -1.906000
H -2.637900 2.213200 -1.400600
H -0.841400 3.370100 -0.063800
H 0.632900 -0.433800 1.262300
C 1.253500 -0.172400 -0.790700
H 0.856500 0.319800 -1.689900
C 1.448300 2.217100 0.986100
H 1.849200 1.808300 1.923600
H 1.315600 3.294300 1.157100
C 2.626700 0.496200 -0.485400
H 3.218700 0.433000 -1.412300
C 2.460100 1.987000 -0.143900
H 2.131300 2.523400 -1.044500
H 3.432900 2.418300 0.129800
C -1.967500 -0.790300 1.412500
H -2.147100 0.169800 1.913600
C -4.154400 -0.696700 0.130800
H -5.088700 -1.231100 -0.085000
C -3.318200 -1.476400 1.155400

H -3.872000 -1.587500 2.096700
H -2.714900 1.821600 0.309000
H -4.447000 0.271400 0.560600
C -3.368100 -0.477200 -1.171000
H -3.208200 -1.454000 -1.651100
H -3.961100 0.120200 -1.877000
C 1.497600 -1.653500 -1.165100
H 0.556900 -2.164700 -1.398200
C 3.422400 -0.276300 0.589000
H 2.913900 -0.219000 1.560500
H 4.398700 0.208100 0.728300
C 3.617600 -1.752600 0.212800
H 4.143500 -2.283400 1.017200
H 4.262700 -1.815700 -0.676300
H -3.140300 -2.493900 0.776000
H -1.368900 -1.391500 2.110500
H 2.081900 -1.668700 -2.097400
C 2.276800 -2.433900 -0.095500
H 1.681400 -2.508800 0.825600
H 2.444600 -3.463900 -0.436200

CT to TT TS for [ag^-g^+a]

DFT energy	-703.997071519
gCP correction	0.1278861995
D3 correction	-0.10393307
gCP-D3 correction	0.0239531295
DFT-gCP-D3 energy	-703.9731183895

H 1.893900 1.959800 1.838400
C 2.375800 1.783100 0.865500
C 2.540500 0.276800 0.653900
H 3.172500 -0.118400 1.465100
C 1.165200 -0.426100 0.803200
H 0.905800 -0.318700 1.867800
C 1.542900 2.395400 -0.259300
C 0.162500 1.716900 -0.505200
H 0.033100 1.675700 -1.595500
C -0.026300 0.208400 -0.004700
H -0.139400 -0.390500 -0.916900
C -0.959400 2.655400 -0.015400
H -0.903600 2.792500 1.073700
C -1.360900 0.095700 0.813500
H -1.198100 0.700400 1.717800
C -2.594800 0.699700 0.082400
H -3.389100 0.746500 0.842800
C -2.334100 2.138000 -0.431700
H -3.123200 2.818300 -0.087600
H 1.380500 3.460900 -0.053500
H -2.381800 2.150700 -1.530500
H -0.794800 3.648700 -0.452700
H 2.133200 2.366200 -1.182200
H 3.356700 2.276500 0.908900
C -3.115000 -0.221200 -1.038300
H -2.353600 -0.301200 -1.828800
H -3.992200 0.244600 -1.508300
C -1.765500 -1.309700 1.320900
H -0.954200 -1.778500 1.885500
C -2.283400 -2.249100 0.219800
H -1.485300 -2.469800 -0.502300
C -3.470200 -1.622100 -0.524800
H -3.788400 -2.266100 -1.355000
C 1.362700 -1.943600 0.537300
H 0.413300 -2.480500 0.571800
C 2.074700 -2.264400 -0.784800
H 1.455700 -1.939100 -1.634400
C 3.265900 -0.059900 -0.666200
H 4.241600 0.445700 -0.679900
H 2.703600 0.333100 -1.523500
C 3.438200 -1.570100 -0.860400
H 3.928900 -1.778600 -1.820000
H -4.328200 -1.548000 0.160100
H 4.096200 -1.972900 -0.076000
H -2.574800 -3.211700 0.660300
H -2.579600 -1.164100 2.046900
H 2.187500 -3.351700 -0.885100
H 1.971500 -2.349400 1.359500

Pseudorotation TS for [ag^-g^+a]

DFT energy	-703.995989565
gCP correction	0.1278256931
D3 correction	-0.10375792
gCP-D3 correction	0.0240677731
DFT-gCP-D3 energy	-703.9719217919

H 2.536000 0.062400 3.411600
C 2.003300 -0.290000 2.519500
C 0.503100 0.088300 2.598500
H 0.373300 0.788100 3.437400
C -0.018100 0.863300 1.340400
H 0.514400 1.825200 1.312200
C 2.651800 0.253300 1.252700
C 1.900800 -0.225600 0.000000
H 1.975100 -1.324100 0.000000
C 0.338200 0.126200 0.000000
H -0.201600 -0.828700 0.000000
C 2.651800 0.253300 -1.252700
H 2.689000 1.351400 -1.277400
C -0.018100 0.863300 -1.340400
H 0.514400 1.825200 -1.312200
C 0.503100 0.088300 -2.598500

H 0.373300 0.788100 -3.437400
C 2.003300 -0.290000 -2.519500
H 2.536000 0.062400 -3.411600
H 2.689000 1.351400 1.277400
H 2.108000 -1.384500 -2.516700
H 3.695300 -0.082300 -1.188200
H 3.695300 -0.082300 1.188200
H 2.108000 -1.384500 2.516700
C -0.358700 -1.149800 -2.921000
H -0.264000 -1.881500 -2.104700
H 0.048800 -1.641200 -3.815200
C -1.498700 1.208300 -1.650100
H -1.947800 1.828500 -0.875300
C -2.386700 -0.009700 -1.945900
H -2.442600 -0.661400 -1.064300
C -1.837500 -0.807700 -3.134400
H -2.424200 -1.721800 -3.294400
C -1.498700 1.208300 1.650100
H -1.947800 1.828500 0.875300
C -2.386700 -0.009700 1.945900
H -2.442600 -0.661400 1.064300
C -0.358700 -1.149800 2.921000
H 0.048800 -1.641200 3.815200
H -0.264000 -1.881500 2.104700
C -1.837500 -0.807700 3.134400
H -2.424200 -1.721800 3.294400
H -1.940000 -0.205700 -4.049600
H -1.940000 -0.205700 4.049600
H -3.413500 0.321500 -2.149500
H -1.487300 1.839400 -2.552100
H -3.413500 0.321500 2.149500
H -1.487300 1.839400 2.552100

Pseudorotation TS for [g$^+$*aa*g$^-$]

DFT energy	-703.994690648
gCP correction	0.1273213358
D3 correction	-0.10387154
gCP-D3 correction	0.0234497958
DFT-gCP-D3 energy	-703.9712408522

H -0.142800 1.752500 0.979100
C -0.206600 0.968100 1.740500
C -1.692500 0.502300 1.788400
H -2.299000 1.200900 1.198700
C -1.928000 -0.931900 1.290000
H -3.003700 -1.090600 1.135200
C 0.778300 -0.148800 1.300800
C 0.344400 -0.897700 0.000000
H 0.939500 -1.821300 0.000000
C -1.174400 -1.338300 0.000000
H -1.187700 -2.435600 0.000000
C 0.778300 -0.148800 -1.300800
H 1.726700 0.359900 -1.066300
C -1.928000 -0.931900 -1.290000
H -1.646800 -1.628700 -2.087900
H -3.003700 -1.090600 -1.135200
C -1.692500 0.502300 -1.788400
H -2.299000 1.200900 -1.198700
C -0.206600 0.968100 -1.740500
H -0.142800 1.752500 -0.979100
H 1.726700 0.359900 1.066300
H -2.077700 0.576300 -2.813900
C 1.122300 -1.131900 -2.446300
H 0.264800 -1.773700 -2.682600
H 1.917800 -1.806700 -2.101200
C 0.248700 1.626800 -3.061500
H 1.172700 2.194300 -2.875100
H -0.504400 2.360200 -3.380700
C 0.521800 0.613400 -4.179800
H -0.407900 0.092200 -4.452300
H 0.859900 1.134300 -5.084800
C 1.568200 -0.413400 -3.728100
H 1.759800 -1.146600 -4.522300
H 2.522000 0.104000 -3.544300
H -2.077700 0.576300 2.813900
H -1.646800 -1.628700 2.087900
C 1.122300 -1.131900 2.446300
H 0.264800 -1.773700 2.682600
H 1.917800 -1.806700 2.101200
C 0.248700 1.626800 3.061500
H -0.504400 2.360200 3.380700
H 1.172700 2.194300 2.875100
C 0.521800 0.613400 4.179800
H -0.407900 0.092200 4.452300
H 0.859900 1.134300 5.084800
C 1.568200 -0.413400 3.728100
H 1.759800 -1.146600 4.522300
H 2.522000 0.104000 3.544300

TS for concerted inversion

DFT energy	-703.994575580
gCP correction	0.1262316416
D3 correction	-0.10023618
gCP-D3 correction	0.0259954616
DFT-gCP-D3 energy	-703.9685801184

H -0.958000 1.644700 1.849900
C -0.502800 0.755300 2.314000
C -1.651800 -0.235600 2.541400
H -2.296900 0.109000 3.361400

C -2.464000 -0.320700 1.250800
H -2.883200 0.679500 1.067700
C 0.517900 0.176500 1.305700
C -0.098600 -0.466900 0.000000
H 0.323000 -1.477200 0.000000
C -1.665800 -0.772500 0.000000
H -1.715000 -1.869400 0.000000
C 0.517900 0.176500 -1.305700
H 1.152600 1.019100 -0.996600
C -2.464000 -0.320700 -1.250800
H -3.326900 -0.989500 -1.370400
H -2.883200 0.679500 -1.067700
C -1.651800 -0.235600 -2.541400
H -2.296900 0.109000 -3.361400
C -0.502800 0.755300 -2.314000
H -0.958000 1.644700 -1.849900
H 1.152600 1.019100 0.996600
H -1.275800 -1.224600 -2.836400
C 1.461600 -0.845400 -1.989200
H 0.894800 -1.752800 -2.245600
H 2.225100 -1.162100 -1.266300
C 0.183700 1.242800 -3.607500
H 0.795900 2.125100 -3.367600
H -0.583600 1.583900 -4.315900
C 1.088800 0.191000 -4.261100
H 0.486700 -0.658900 -4.613000
H 1.575900 0.614700 -5.149000
C 2.135100 -0.304900 -3.256500
H 2.768900 -1.080300 -3.706400
H 2.802700 0.528300 -2.989400
H -1.275800 -1.224600 2.836400
H -3.326900 -0.989500 1.370400
C 1.461600 -0.845400 1.989200
H 0.894800 -1.752800 2.245600
H 2.225100 -1.162100 1.266300
C 0.183700 1.242800 3.607500
H -0.583600 1.583900 4.315900
H 0.795900 2.125100 3.367600
C 1.088800 0.191000 4.261100
H 0.486700 -0.658900 4.613000
H 1.575900 0.614700 5.149000
C 2.135100 -0.304900 3.256500
H 2.768900 -1.080300 3.706400
H 2.802700 0.528300 2.989400

Perhydro[6]helicene

Ground state

DFT energy	-1016.10996239
gCP correction	0.1847658923
D3 correction	-0.16057233
gCP-D3 correction	0.0241935623
DFT-gCP-D3 energy	-1016.0857688277

H 0.883200 -0.802800 0.485700
C 1.268600 0.182500 0.201800
C 0.511100 2.478800 -0.632800
C 2.174100 2.323300 1.291300
C 1.029900 3.130300 0.664300
C 1.782100 0.848800 1.510800
C 0.084400 0.997700 -0.408400
H 1.355200 2.471000 -1.338100
H 0.217400 3.228600 1.395700
H 0.944900 0.850600 2.224600
H 2.464700 2.767100 2.253500
H 1.366700 4.153000 0.445000
H -0.124100 0.580100 -1.399600
C -1.255100 0.910200 0.401400
H -1.074700 1.359100 1.388100
C -0.614100 3.296100 -1.292600
H -0.768700 2.928900 -2.316400
H -0.301900 4.345100 -1.389400
C -2.378900 1.757700 -0.269700
H -3.217500 1.799500 0.440400
C -1.931200 3.210100 -0.513400
H -1.816900 3.714900 0.455400
H -2.721500 3.754600 -1.048200
C 2.443600 -0.078200 -0.794800
H 2.878700 0.893500 -1.064300
C 4.066600 -0.255300 1.176500
H 4.816900 -0.898100 1.657000
C 3.586400 -0.904900 -0.133800
H 4.431800 -0.886100 -0.840000
H 3.058900 2.394500 0.645100
H 4.580000 0.686200 0.938900
C 2.914500 0.024100 2.151200
H 2.500600 -0.927900 2.510800
H 3.294900 0.545600 3.040300
C 2.039100 -0.765900 -2.120600
H 1.236900 -0.215900 -2.625400
C 3.206600 -2.390800 0.043000
H 2.392400 -2.496800 0.772300
H 4.065000 -2.931600 0.464900
C 2.779100 -3.036900 -1.283200
H 3.643900 -3.069600 -1.962800
H 2.472800 -4.078100 -1.118500
C 1.648900 -2.242500 -1.952400
H 0.734500 -2.326500 -1.348100
C -1.767200 -0.535600 0.670100
H -0.912700 -1.153500 0.976400
C -2.929600 1.084300 -1.542300
H -2.157200 1.035300 -2.321500

H -3.738000 1.703700 -1.954700
C -3.448300 -0.330200 -1.252500
H -3.788300 -0.808200 -2.181400
H -4.332200 -0.260800 -0.604100
C -2.366500 -1.207100 -0.594500
H -1.555200 -1.307900 -1.331200
C -2.748600 -0.591200 1.863500
H -3.634300 0.028500 1.671400
H -2.260800 -0.159900 2.748400
C -2.866500 -2.632100 -0.274000
H -3.345000 -3.063600 -1.163900
H -1.996900 -3.268500 -0.052100
C -3.827600 -2.684600 0.924100
H -4.766300 -2.169600 0.676100
H -4.097100 -3.726700 1.139500
C -3.201400 -2.028300 2.162600
H -2.332100 -2.621600 2.484300
H -3.910700 -2.035800 3.000400
H 1.409200 -2.676300 -2.932000
H 2.904800 -0.715100 -2.797900

Pseudorotation TS for [a$g^{-}g^{+}$a]

DFT energy	-1016.08430546
gCP correction	0.1844333284
D3 correction	-0.15867707
gCP-D3 correction	0.0257562584
DFT-gCP-D3 energy	-1016.0585492016

H -3.404600 3.522300 -0.536200
C -2.506700 3.111600 -0.057600
C -2.597300 1.568200 0.030500
H -3.458800 1.261200 -0.574600
C -1.363700 0.817100 -0.582700
H -1.345400 1.050800 -1.657600
C -1.249200 3.551600 -0.790100
C 0.000100 2.964200 -0.117200
H 0.000200 3.352800 0.913100
C 0.000100 1.363000 0.004800
H 0.000000 1.131500 1.076800
C 1.249400 3.551500 -0.790200
H 1.290200 3.256900 -1.848000
C 1.363800 0.817000 -0.582700
H 1.345500 1.050700 -1.657600
C 2.597400 1.568100 0.030600
H 3.458900 1.260900 -0.574400
C 2.506900 3.111500 -0.057800
H 3.404900 3.522000 -0.536500
H -1.290000 3.257000 -1.847900
H 2.480300 3.539300 0.954800
H 1.169900 4.646600 -0.784500
H -1.169600 4.646700 -0.784400
H -2.479900 3.539300 0.955000
C 2.892800 1.115000 1.473500
H 2.050900 1.392100 2.125500
H 3.761500 1.672200 1.850100
C 1.678700 -0.710700 -0.494800
H 0.802700 -1.270800 -0.819700
C 2.000800 -1.194700 0.943400
H 1.112100 -1.007200 1.563900
C 3.151500 -0.391100 1.572300
H 3.277700 -0.690500 2.622000
C -1.678700 -0.710600 -0.494800
C -2.001000 -1.194600 0.943400
H -1.112300 -1.007200 1.564000
C -2.892900 1.115100 1.473400
H -3.761600 1.672400 1.849900
H -2.051000 1.392100 2.125500
C -3.151700 -0.391000 1.572200
H -3.278000 -0.690400 2.621800
H 4.101300 -0.624900 1.072900
H -4.101500 -0.624700 1.072700
C 2.781200 -1.147300 -1.491500
H 2.500700 -0.821700 -2.502700
H 3.741300 -0.665900 -1.270200
C 2.253900 -2.718000 0.960400
H 1.309400 -3.233500 0.730900
H 2.539100 -3.033900 1.973300
C 3.315400 -3.170200 -0.055200
H 3.395400 -4.265000 -0.046300
H 4.303200 -2.788800 0.238700
C 2.979400 -2.671700 -1.468100
H 2.057100 -3.162200 -1.814400
H 3.769200 -2.961400 -2.173400
C -2.254200 -2.717900 0.960300
H -2.539400 -3.033800 1.973200
H -0.802700 -1.270800 -0.819500
H -1.309700 -3.233400 0.730900
C -2.781100 -1.147200 -1.491600
H -2.500500 -0.821600 -2.502800
H -3.741200 -0.665600 -1.270400
C -3.315600 -3.170000 -0.055300
H -3.395700 -4.264800 -0.046400
H -4.303400 -2.788500 0.238500
C -2.979400 -2.671600 -1.468200
H -2.057100 -3.162100 -1.814400
H -3.769200 -2.961200 -2.173500

CT to TT TS for [a$g^{-}g^{+}$a]

DFT energy	-1016.08372276
gCP correction	0.1842261355

D3 correction -0.15852018
gCP-D3 correction 0.0257059555
DFT-gCP-D3 energy -1016.0580168045

H 2.886000 3.764300 -0.573000
C 2.119300 3.139400 -1.045700
C 2.407800 1.655900 -0.688300
H 3.375900 1.638500 -0.169300
C 1.384000 1.014500 0.290000
H 1.488400 1.542500 1.251100
C 0.730700 3.733000 -0.685200
C -0.387900 2.801100 -0.124200
H -1.266200 2.967200 -0.757700
C -0.092000 1.268600 -0.163200
H -0.191200 0.944400 -1.208600
C -0.793100 3.230300 1.298500
H 0.009900 2.959900 1.998500
C -1.146700 0.466900 0.693900
H -0.660700 0.252800 1.657500
C -2.439300 1.247600 1.065100
H -2.958300 0.630100 1.811500
C -2.127500 2.603300 1.745800
H -2.119200 2.475700 2.835700
H 0.884900 4.535900 0.045000
H -2.950000 3.299000 1.532600
H -0.869500 4.323500 1.344400
H 0.336500 4.237200 -1.575000
H 2.281400 3.261500 -2.123300
C -3.432300 1.373200 -0.109700
H -3.019700 2.001700 -0.909900
H -4.337200 1.886500 0.243600
C -1.520400 -0.907100 0.060700
H -0.610800 -1.328900 -0.381200
C -2.537700 -0.775800 -1.104800
H -2.056300 -0.181300 -1.897100
C -3.797600 0.000600 -0.684800
H -4.462600 0.115000 -1.551900
C 1.791400 -0.463800 0.586600
C 2.077500 -1.304600 -0.688500
H 1.141000 -1.377500 -1.259900
C 2.608900 0.807200 -1.959600
H 3.344400 1.304800 -2.605800
H 1.674200 0.763100 -2.538800
C 3.078100 -0.610800 -1.625900
H 3.191300 -1.204500 -2.543400
H -4.367500 -0.564700 0.065000
H 4.075200 -0.561700 -1.167200
C -1.994000 -1.937200 1.110400
H -1.223700 -2.041100 1.887200
H -2.896900 -1.581600 1.623700
C -2.854500 -2.161600 -1.707200
H -1.949600 -2.544000 -2.202300
H -3.615500 -2.057000 -2.492800
C -3.308500 -3.191200 -0.660200
H -3.458600 -4.167500 -1.139100
H -4.283200 -2.899600 -0.244600
C -2.285500 -3.306800 0.478200
H -1.349200 -3.727300 0.080600
H -2.641300 -4.009200 1.243200
C 2.490700 -2.745600 -0.319900
H 2.745500 -3.298800 -1.234200
H 0.955400 -0.950700 1.104100
H 1.622700 -3.259500 0.119900
C 2.973600 -0.538300 1.582300
H 2.707900 0.015100 2.493400
H 3.862300 -0.040600 1.172000
C 3.652700 -2.809200 0.682300
H 3.858400 -3.853700 0.949700
H 4.571400 -2.423600 0.218400
C 3.335500 -1.987900 1.939300
H 2.489700 -2.451700 2.468900
H 4.185300 -2.005300 2.634100

Pseudorotation TS for [g$^+$*aa*g$^-$]

DFT energy -1016.08020433
gCP correction 0.1849392540
D3 correction -0.16097649
gCP-D3 correction 0.023962764
DFT-gCP-D3 energy -1016.056241566

H 1.682300 -1.641700 0.941300
C 1.526500 -0.894000 1.724600
C 2.817600 -0.022700 1.777800
H 3.597600 -0.518100 1.186600
C 2.636600 1.419700 1.288000
H 3.621700 1.880400 1.134600
C 0.249100 -0.103800 1.310100
C 0.467000 0.738700 0.000000
H -0.343000 1.465400 0.000000
C 1.797900 1.591800 0.000000
H 1.495600 2.647300 0.000000
C 0.249100 -0.103800 -1.310100
H -0.503000 -0.864500 -1.057600
C 2.636600 1.419700 -1.288000
H 2.167200 2.004300 -2.087700
H 3.621700 1.880400 -1.134600
C 2.817600 -0.022700 -1.777800
H 3.597600 -0.518100 -1.186600
C 1.526500 -0.894000 -1.724600
H 1.682300 -1.641700 -0.941300
H -0.503000 -0.864500 1.057600
H 3.207500 0.009800 -2.803600

C -0.354500 0.727200 -2.495000
H 0.355000 1.528400 -2.737200
C 1.321700 -1.692300 -3.026900
H 0.618000 -2.515100 -2.842000
H 2.270700 -2.165100 -3.314500
C 0.796100 -0.820100 -4.170400
H 1.547500 -0.059300 -4.426700
H 0.652200 -1.425800 -5.075700
C -0.517400 -0.116700 -3.794200
H -0.753900 0.597900 -4.598600
H 3.207500 0.009800 2.803600
H 2.167200 2.004300 2.087700
C -0.354500 0.727200 2.495000
H 0.355000 1.528400 2.737200
C 1.321700 -1.692300 3.026900
H 2.270700 -2.165100 3.314500
H 0.618000 -2.515100 2.842000
C 0.796100 -0.820100 4.170400
H 1.547500 -0.059300 4.426700
H 0.652200 -1.425800 5.075700
C -0.517400 -0.116700 3.794200
H -0.753900 0.597900 4.598600
C -1.707300 1.419700 2.193600
H -1.652500 2.029200 1.284900
C -1.712000 -1.092300 3.721700
H -1.799100 -1.621700 4.680600
H -1.534900 -1.866100 2.962800
C -3.027100 -0.370500 3.396800
H -3.848400 -1.094100 3.312400
H -3.286400 0.301900 4.228300
C -2.898200 0.453600 2.109200
H -2.776900 -0.223400 1.251800
C -1.707300 1.419700 -2.193600
H -1.652500 2.029200 -1.284900
C -1.712000 -1.092300 -3.721700
H -1.534900 -1.866100 -2.962800
H -1.799100 -1.621700 -4.680600
C -3.027100 -0.370500 -3.396800
H -3.848400 -1.094100 -3.312400
H -3.286400 0.301900 -4.228300
C -2.898200 0.453600 -2.109200
H -2.776900 -0.223400 -1.251800
H -3.820700 1.019100 -1.924500
H -1.900500 2.129800 -3.011700
H -3.820700 1.019100 1.924500
H -1.900500 2.129800 3.011700

Perhydro[8]helicene

Ground state

DFT energy	-1328.19954293
gCP correction	0.2421984781
D3 correction	-0.21826653
gCP-D3 correction	0.0239319481
DFT-gCP-D3 energy	-1328.1756109819

H 0.552700 0.503200 -1.498000
C 0.141700 1.207400 -0.767800
C 0.437500 2.000700 1.648900
C 0.203400 3.716900 -0.215800
C 0.817100 3.409900 1.155400
C 0.547200 2.630700 -1.254000
C 0.808300 0.899500 0.612100
H -0.657100 1.992200 1.752800
H 1.907900 3.514900 1.091700
H 1.642100 2.635800 -1.360300
H 0.561100 4.690000 -0.580000
H 0.483300 4.152900 1.893000
H 0.380200 -0.036600 0.987300
C 2.360000 0.676900 0.554800
H 2.818700 1.617800 0.219800
C 1.022300 1.687700 3.037400
H 0.528400 0.792700 3.440700
H 0.784700 2.505200 3.731900
C 2.943600 0.383400 1.971300
H 4.038800 0.428500 1.882100
C 2.537700 1.461300 2.992600
H 3.040200 2.403500 2.735600
H 2.907300 1.182600 3.989000
C -1.417700 1.023700 -0.801000
H -1.836600 1.770000 -0.118100
C -1.558400 2.751900 -2.691200
H -1.926600 2.922300 -3.712300
C -1.990000 1.354100 -2.213900
H -3.085800 1.372500 -2.120100
H -0.884700 3.815200 -0.107900
H -2.043700 3.511100 -2.063200
C -0.038200 2.942400 -2.643100
H 0.437000 2.287900 -3.386500
H 0.221700 3.970000 -2.932600
C -1.924600 -0.375900 -0.327600
H -1.373700 -0.639800 0.581800
C -1.660100 0.255900 -3.243900
H -0.579400 0.217500 -3.434400
H -2.128400 0.510900 -4.204600
C -2.137600 -1.123500 -2.776600
H -3.235400 -1.141400 -2.774500
H -1.822700 -1.895500 -3.492300

C -1.588600 -1.473800 -1.379400
H -0.493000 -1.507000 -1.475900
C 2.826300 -0.416300 -0.451200
H 2.268700 -0.284900 -1.387700
C 2.610300 -1.043100 2.450900
H 1.528700 -1.159000 2.602400
H 3.074100 -1.209400 3.433000
C 3.096200 -2.103300 1.454300
H 2.804200 -3.106300 1.794900
H 4.194200 -2.096900 1.431400
C 2.527900 -1.856600 0.044800
H 1.435100 -1.961400 0.124600
C 4.312100 -0.251000 -0.845800
H 4.963400 -0.335100 0.034100
H 4.466700 0.761400 -1.243500
C 3.006700 -2.900200 -0.987000
H 2.840500 -3.912200 -0.593000
H 2.382100 -2.813400 -1.888500
C 4.477800 -2.723100 -1.394500
H 5.133200 -2.942400 -0.540100
H 4.737700 -3.450600 -2.174400
C 4.745600 -1.294000 -1.887700
H 4.188400 -1.124700 -2.821500
H 5.808100 -1.166100 -2.132100
C -2.039000 -2.866100 -0.899700
H -1.837000 -3.609900 -1.682600
C -3.439400 -0.408000 0.056800
H -4.021600 -0.179200 -0.846300
C -3.887200 -1.825500 0.523600
H -4.986100 -1.795300 0.596000
C -3.522800 -2.904800 -0.511300
H -4.139700 -2.761400 -1.408600
H -3.782200 -3.897700 -0.119200
H -1.427200 -3.160100 -0.035800
C -3.869300 0.621600 1.129900
H -4.969300 0.627200 1.159000
H -3.577700 1.638500 0.845000
C -3.373500 -2.166800 1.939500
H -3.760300 -3.152900 2.231200
H -2.279000 -2.257200 1.942200
C -3.355400 0.294700 2.541000
H -3.727000 1.043400 3.252800
H -2.258800 0.362200 2.570100
C -3.792200 -1.111400 2.973200
H -4.887000 -1.128000 3.082300
H -3.376900 -1.357400 3.959100

Pseudorotation TS for [g$^+$*aa*g$^-$]

DFT energy	-1328.17155093
gCP correction	0.2416958296
D3 correction	-0.21653066
gCP-D3 correction	0.0251651696
DFT-gCP-D3 energy	-1328.1463857604

H -0.765600 -3.138900 -1.137100
C -1.610800 -2.741400 -0.569900
C -1.735600 -3.606100 0.719400
H -1.173800 -4.535800 0.568400
C -1.265100 -2.912300 2.007800
H -1.103300 -3.666700 2.789400
C -1.273700 -1.248000 -0.286100
C 0.017900 -1.112000 0.605300
H -0.005800 -0.091800 0.986100
C 0.006200 -2.037400 1.888600
H -0.026600 -1.369700 2.759500
C 1.354700 -1.196300 -0.236000
H 1.116000 -0.787000 -1.228200
C 1.300700 -2.860600 2.057300
H 2.101200 -2.199600 2.409500
H 1.154100 -3.594700 2.861200
C 1.770300 -3.575200 0.788500
H 1.123800 -4.440000 0.596400
C 1.799400 -2.676300 -0.485500
H 1.068600 -3.091000 -1.184900
H -1.014000 -0.803900 -1.257300
H 2.768200 -3.993100 0.973700
C 2.507500 -0.292300 0.343600
H 2.659400 -0.599600 1.386200
C 3.159700 -2.783500 -1.198000
H 3.069700 -2.416000 -2.228600
H 3.438000 -3.843100 -1.278100
C 4.245500 -1.997700 -0.464300
H 4.381100 -2.409300 0.546200
H 5.212400 -2.108700 -0.973800
C 3.880700 -0.509600 -0.364900
H 4.639800 -0.030800 0.271000
H -2.777400 -3.919600 0.864900
H -2.080300 -2.283500 2.382800
C -2.534600 -0.463700 0.235800
H -2.813100 -0.900700 1.202700
C -2.838800 -2.899800 -1.484800
H -3.066900 -3.967700 -1.607200
H -2.593800 -2.524400 -2.487900
C -4.059700 -2.155800 -0.942300
H -4.377300 -2.615200 0.004300
H -4.908700 -2.260300 -1.631700
C -3.768000 -0.667100 -0.698000
H -4.639400 -0.257900 -0.166700
C -2.326800 1.058600 0.502300
H -1.356300 1.197200 0.997600
C -3.649600 0.141300 -2.004100
H -4.545500 -0.029600 -2.616700

H -2.798400 -0.206300 -2.604800
C -3.487600 1.637500 -1.714100
H -3.363600 2.198100 -2.650800
H -4.410900 2.013600 -1.252800
C -2.281100 1.908400 -0.796500
H -1.385000 1.603500 -1.359100
C 2.177900 1.235000 0.395500
H 1.124700 1.354400 0.682200
C 3.967800 0.190700 -1.735500
H 3.245200 -0.241700 -2.440500
H 4.962300 0.011100 -2.166900
C 3.703900 1.692500 -1.615900
H 3.738600 2.168000 -2.605700
H 4.505400 2.156300 -1.025000
C 2.333000 1.956400 -0.972000
H 1.581000 1.531500 -1.655100
C 2.026000 3.463600 -0.840300
H 0.955900 3.589000 -0.625100
H 2.205200 3.959000 -1.804300
C 2.985300 1.948000 1.505900
H 4.064000 1.849700 1.323500
C 2.633600 3.439100 1.613900
H 3.240100 3.914900 2.395600
C 2.828500 4.156300 0.271500
H 2.521700 5.207300 0.351500
H 3.897400 4.166900 0.016200
C -3.370800 1.604500 1.505700
H -4.389900 1.464100 1.122200
C -2.121700 3.409100 -0.468100
C -3.166700 3.933100 0.527900
C -3.159300 3.094500 1.812800
H -3.930300 3.449500 2.509000
H -4.168700 3.902800 0.077900
H -2.152300 3.993800 -1.397800
H -2.964900 4.987300 0.758100
H -1.125100 3.571300 -0.033800
H -2.193300 3.226400 2.323400
H -3.311900 1.019800 2.433900
H 1.583900 3.541500 1.928700
H 2.791900 1.448300 2.464900

Pseudorotation TS for [a$g^{-}g^{+}$a]

DFT energy	-1328.16747606
gCP correction	0.2440246614
D3 correction	-0.22125314
gCP-D3 correction	0.0227715214
DFT-gCP-D3 energy	-1328.1447045386

H -3.743100 -1.437100 3.403700
C -3.574200 -0.830900 2.504800
C -2.202600 -0.114800 2.581900
H -1.696500 -0.494000 3.476400
C -1.244400 -0.431700 1.381000
H -1.065300 -1.513700 1.378300
C -3.681900 -1.682300 1.249400
C -3.392200 -0.838600 0.000000
H -4.144800 -0.034300 0.000000
C -1.956900 -0.110600 0.000000
H -2.166700 0.963700 0.000000
C -3.681900 -1.682300 -1.249400
H -2.998800 -2.541400 -1.300700
C -1.244400 -0.431700 -1.381000
H -1.065300 -1.513700 -1.378300
C -2.202600 -0.114800 -2.581900
H -1.696500 -0.494000 -3.476400
C -3.574200 -0.830900 -2.504800
H -3.743100 -1.437100 -3.403700
H -2.998800 -2.541400 1.300700
H -4.381500 -0.084900 -2.486100
H -4.691500 -2.104800 -1.163000
H -4.691500 -2.104800 1.163000
H -4.381500 -0.084900 2.486100
C -2.378300 1.404100 -2.781800
H -2.902000 1.828600 -1.913000
H -3.039400 1.575700 -3.642300
C 0.127800 0.244900 -1.721000
H 0.806000 0.082800 -0.881000
C -0.023200 1.782200 -1.897700
H -0.428900 2.183100 -0.961100
C -1.047400 2.137600 -2.992200
H -1.211100 3.224100 -2.995400
C 0.127800 0.244900 1.721000
C -0.023200 1.782200 1.897700
H -0.428900 2.183100 0.961100
C -2.378300 1.404100 2.781800
H -3.039400 1.575700 3.642300
H -2.902000 1.828600 1.913000
C -1.047400 2.137600 2.992200
H -1.211100 3.224100 2.995400
H -0.655400 1.890100 -3.986400
H -0.655400 1.890100 3.986400
C 0.842500 -0.351300 -2.987300
H 0.237300 -0.097200 -3.865000
C 1.348900 2.447100 -2.116700
H 1.944000 2.337200 -1.199700
H 1.219300 3.527800 -2.267100
C 2.119500 1.846100 -3.300300
H 3.122500 2.290800 -3.359000
H 1.613900 2.117700 -4.236900
C 2.231000 0.311900 -3.226600
H 2.570000 -0.035500 -4.215900
C 1.348900 2.447100 2.116700

H 1.219300 3.527800 2.267100
H 0.806000 0.082800 0.881000
H 1.944000 2.337200 1.199700
C 0.842500 -0.351300 2.987300
H 0.237300 -0.097200 3.865000
C 2.119500 1.846100 3.300300
H 3.122500 2.290800 3.359000
H 1.613900 2.117700 4.236900
C 2.231000 0.311900 3.226600
H 2.570000 -0.035500 4.215900
C 3.291600 -0.179000 2.220600
H 3.042200 0.150300 1.202900
H 4.258300 0.284500 2.460800
C 3.291600 -0.179000 -2.220600
H 4.258300 0.284500 -2.460800
H 3.042200 0.150300 -1.202900
C 1.016000 -1.886600 3.015400
H 0.060100 -2.397100 2.852100
H 1.331300 -2.163400 4.032700
C 1.016000 -1.886600 -3.015400
H 1.331300 -2.163400 -4.032700
H 0.060100 -2.397100 -2.852100
C 2.070300 -2.399800 -2.026200
H 2.179300 -3.487600 -2.125900
H 1.729200 -2.218700 -0.999700
C 3.425100 -1.709800 -2.242900
H 3.831100 -2.018600 -3.218000
H 4.148400 -2.040700 -1.486200
C 2.070300 -2.399800 2.026200
H 1.729200 -2.218700 0.999700
H 2.179300 -3.487600 2.125900
C 3.425100 -1.709800 2.242900
H 4.148400 -2.040700 1.486200
H 3.831100 -2.018600 3.218000

CT to TT TS for [a*g*$^-$*g*$^+$a]

DFT energy	-1328.16272608
gCP correction	0.2444634076
D3 correction	-0.22211185
gCP-D3 correction	0.0223515576
DFT-gCP-D3 energy	-1328.1403745224

H -2.560100 2.181900 -3.248600
C -2.492200 2.575900 -2.226500
C -2.718000 1.436100 -1.205800
H -3.251100 0.641400 -1.740100
C -1.378300 0.816600 -0.705900
H -0.834100 0.471000 -1.595800
C -1.143100 3.275600 -2.030200
C -0.751900 3.332000 -0.542500
H -1.644400 3.669700 -0.006600
C -0.434800 1.885500 0.005100
H -0.703700 1.894700 1.065600
C 0.312200 4.456600 -0.328500
H 0.565000 4.858800 -1.315900
C 1.115300 1.635800 -0.080300
H 1.388100 1.833100 -1.126200
C 1.873500 2.669200 0.805300
H 2.944000 2.488900 0.650700
C 1.640500 4.136100 0.394400
H 2.463500 4.437500 -0.264900
H -0.350500 2.759700 -2.591800
H 1.740800 4.768600 1.285400
H -0.183200 5.285900 0.189700
H -1.191500 4.290400 -2.443700
H -3.303700 3.310800 -2.131200
C 1.636800 2.410000 2.305500
H 0.578900 2.548800 2.568300
H 2.192900 3.155300 2.890000
C 1.650700 0.208400 0.276500
H 1.043200 -0.522200 -0.269800
C 1.472400 -0.086300 1.796500
H 0.395300 -0.068300 2.010300
C 2.099000 1.000900 2.692500
H 1.847100 0.788000 3.740600
C -1.744200 -0.445400 0.154700
C -2.619800 -0.039700 1.375400
H -2.047500 0.684100 1.972400
C -3.636800 1.839000 -0.028200
H -4.585500 2.218800 -0.431900
H -3.197100 2.669900 0.535300
C -3.913300 0.675100 0.934700
H -4.442700 1.045100 1.823600
H 3.194500 0.964800 2.631300
H -4.598200 -0.033300 0.453400
C 3.141600 -0.020600 -0.156400
H 3.755900 0.706100 0.388900
C 1.983900 -1.493800 2.155800
H 1.351700 -2.243200 1.660300
H 1.868100 -1.664000 3.235100
C 3.447000 -1.711200 1.748600
H 3.751800 -2.741100 1.979500
H 4.093900 -1.062000 2.353400
C 3.694700 -1.418900 0.256700
H 4.785200 -1.366100 0.108800
C -2.888300 -1.252800 2.285800
H -3.517100 -0.948900 3.134200
H -0.818000 -0.868000 0.552800
H -1.934500 -1.587300 2.717300
C -2.474100 -1.587200 -0.639900
H -3.448900 -1.196700 -0.952500
C -3.550900 -2.416600 1.537500

H -3.645300 -3.285700 2.202900
H -4.575800 -2.133900 1.263100
C -2.788700 -2.819300 0.262000
H -3.461700 -3.462900 -0.327300
C 3.195100 -2.559100 -0.653000
H 3.699500 -3.490800 -0.361400
H 2.124900 -2.733400 -0.492400
C 3.432700 0.204100 -1.659400
H 4.525500 0.238900 -1.783700
H 3.066100 1.180800 -1.994100
C 2.884000 -0.910400 -2.561400
H 3.140600 -0.701900 -3.608200
H 1.786600 -0.930500 -2.512100
C 3.446000 -2.276100 -2.141900
H 4.529800 -2.286600 -2.332300
H 3.014700 -3.076400 -2.757200
C -1.536600 -3.671200 0.548000
H -0.811000 -3.104800 1.147200
H -1.822400 -4.540100 1.156400
C -1.783000 -2.080800 -1.931700
H -1.523600 -1.240900 -2.587100
H -2.518700 -2.679000 -2.489900
C -0.547500 -2.952300 -1.673500
H 0.236000 -2.341800 -1.210500
H -0.136000 -3.310200 -2.626400
C -0.869100 -4.138300 -0.753800
H 0.043000 -4.708400 -0.534000
H -1.548300 -4.827100 -1.278200

Perhydro[10]helicene

Ground state

DFT energy	-1640.28953435
gCP correction	0.2998082079
D3 correction	-0.27651769
gCP-D3 correction	0.0232905179
DFT-gCP-D3 energy	-1640.2662438321

H -2.715800 -0.540000 0.180500
C -2.951300 0.190300 -0.601400
C -2.209200 2.452300 -1.534900
C -2.659100 0.519800 -3.131500
C -1.834200 1.786700 -2.874200
C -2.563000 -0.472100 -1.956100
C -2.088200 1.464800 -0.335300
H -3.269800 2.733000 -1.615000
H -0.766800 1.530100 -2.892100
H -1.505600 -0.766500 -1.884200
H -2.314500 0.025600 -4.050400
H -1.986200 2.507500 -3.689600
H -2.521200 1.977200 0.530000
C -0.583300 1.182500 0.013600
H -0.139000 0.731900 -0.879400
C -1.422500 3.750800 -1.281800
H -1.906600 4.307700 -0.468200
H -1.479300 4.397300 -2.168400
C 0.190000 2.511600 0.264300
H 1.257400 2.254700 0.334600
C 0.043200 3.486100 -0.918200
H 0.568300 3.072000 -1.789700
H 0.548200 4.432600 -0.680900
C -4.493000 0.436500 -0.522200
H -4.764900 1.136100 -1.324000
C -4.885900 -1.533900 -2.108300
H -5.416000 -2.488700 -2.228900
C -5.300800 -0.869700 -0.782800
H -6.355600 -0.567900 -0.881600
H -3.704100 0.802300 -3.313000
H -5.211900 -0.897600 -2.942200
C -3.371000 -1.759900 -2.202100
H -3.067100 -2.516800 -1.466100
H -3.114600 -2.173400 -3.187100
C -4.990200 1.066100 0.801000
H -4.455900 1.995900 1.025400
C -5.241400 -1.843600 0.413500
H -4.221200 -2.227500 0.547600
H -5.868300 -2.718600 0.193300
C -5.704900 -1.180600 1.719400
H -6.775000 -0.937000 1.639300
H -5.607200 -1.882400 2.557900
C -4.919700 0.107700 1.999800
H -3.873200 -0.143600 2.222800
C -0.358000 0.194400 1.202600
H -1.041200 -0.649500 1.064500
C -0.189300 3.159700 1.610900
H -1.231200 3.505300 1.592400
H 0.425100 4.057600 1.763900
C -0.004400 2.189300 2.783800
H -0.363300 2.649500 3.714800
H 1.067200 2.002000 2.931700
C -0.758700 0.865700 2.550800
H -1.824600 1.126700 2.478100
C 1.089200 -0.410000 1.293100
H 1.759800 0.424000 1.521400
C -0.619900 -0.113500 3.730500
H -0.851200 0.405200 4.671100
H -1.375900 -0.904000 3.626500
C 0.774100 -0.747600 3.810900
H 1.511300 0.021000 4.080500
H 0.801500 -1.491300 4.619300
C 1.201200 -1.405100 2.485900
H 2.264000 -1.668600 2.589100

C 1.605800 -1.085100 -0.016300
H 1.386900 -0.407100 -0.848400
C 0.449000 -2.722000 2.209800
H -0.633700 -2.544200 2.171500
H 0.612000 -3.413700 3.047700
C 0.897200 -3.376700 0.895000
H 0.273600 -4.255800 0.681400
H 1.921300 -3.753600 1.012700
C 0.816000 -2.396500 -0.292100
H -0.243800 -2.122400 -0.396900
C 1.245900 -3.033600 -1.626200
H 0.933300 -2.375800 -2.449200
H 0.712600 -3.982500 -1.775700
C 3.152100 -1.344700 -0.053400
H 3.384900 -2.077300 0.731500
C 2.759600 -3.266300 -1.704600
H 3.029400 -3.651800 -2.697400
H 3.045000 -4.048300 -0.988000
C 3.577800 -1.997300 -1.403900
H 4.623900 -2.316800 -1.290700
C 4.035600 -0.099900 0.242600
H 3.609100 0.428700 1.105500
C 3.564200 -0.979600 -2.562400
H 2.540900 -0.637900 -2.767300
H 3.908500 -1.474700 -3.480900
C 4.058400 0.913000 -0.932700
H 3.033000 1.290000 -1.065600
C 4.451700 0.236300 -2.259700
H 4.381400 0.968500 -3.075900
H 5.501600 -0.085100 -2.229600
C 4.944000 2.129600 -0.586800
H 4.454400 2.698800 0.217400
H 4.999400 2.804100 -1.452100
C 5.466400 -0.488900 0.680500
H 5.981000 -1.048300 -0.111700
H 5.406000 -1.167000 1.542900
C 6.304400 0.746200 1.043500
H 5.860200 1.237900 1.922200
H 7.318700 0.444500 1.335400
C 6.357400 1.746400 -0.120500
H 6.921500 1.304900 -0.953900
H 6.907100 2.648800 0.177100
H -5.313500 0.604300 2.896000
H -6.041600 1.356000 0.655300

Pseudorotation TS for [a*g*⁻*g*⁺a]

DFT energy -1640.26019560
gCP correction 0.3005160739
D3 correction -0.27710371
gCP-D3 correction 0.0234123639
DFT-gCP-D3 energy -1640.2367832361

H -2.890700 -2.983200 3.402000
C -3.116900 -2.390500 2.506600
C -2.427200 -1.005500 2.582400
H -1.794400 -1.013200 3.476800
C -1.465200 -0.699200 1.380400
H -0.693700 -1.477400 1.369900
C -2.712800 -3.137900 1.246700
C -2.973100 -2.280000 0.000000
H -4.053600 -2.067000 0.000000
C -2.233100 -0.850800 0.000000
H -3.028100 -0.098600 0.000000
C -2.712800 -3.137900 -1.246700
H -1.656400 -3.437000 -1.292800
C -1.465200 -0.699200 -1.380400
H -0.693700 -1.477400 -1.369900
C -2.427200 -1.005500 -2.582400
H -1.794400 -1.013200 -3.476800
C -3.116900 -2.390500 -2.506600
H -2.890700 -2.983200 -3.402000
H -1.656400 -3.437000 1.292800
H -4.208500 -2.260600 -2.496600
H -3.285800 -4.070200 -1.158600
H -3.285800 -4.070200 1.158600
H -4.208500 -2.260600 2.496600
C -3.460600 0.120200 -2.782700
H -4.132100 0.159500 -1.912700
H -4.098100 -0.130400 -3.641600
C -0.740700 0.649000 -1.725500
H -0.103100 0.919700 -0.882000
C -1.767900 1.804400 -1.906900
H -2.329700 1.890700 -0.968700
C -2.808800 1.490200 -2.998700
H -3.574600 2.278200 -3.004200
C -0.740700 0.649000 1.725500
C -1.767900 1.804400 1.906900
H -2.329700 1.890700 0.968700
C -3.460600 0.120200 2.782700
H -4.098100 -0.130400 3.641600
H -4.132100 0.159500 1.912700
C -2.808800 1.490200 2.998700
H -3.574600 2.278200 3.004200
H -2.347300 1.512900 -3.993900
H -2.347300 1.512900 3.993900
C 0.201600 0.578200 -2.987300
H -0.434200 0.412800 -3.865300
C -1.056200 3.150300 -2.133300
H -0.504900 3.418500 -1.221500
H -1.800700 3.944400 -2.283600
C -0.089600 3.107100 -3.322300
H 0.455100 4.058200 -3.399700

H -0.663400 3.011800 -4.253900
C 0.913700 1.942400 -3.236700
H 1.393800 1.872000 -4.223600
C -1.056200 3.150300 2.133300
H -1.800700 3.944400 2.283600
H -0.103100 0.919700 0.882000
H -0.504900 3.418500 1.221500
C 0.201600 0.578200 2.987300
H -0.434200 0.412800 3.865300
C -0.089600 3.107100 3.322300
H 0.455100 4.058200 3.399700
H -0.663400 3.011800 4.253900
C 0.913700 1.942400 3.236700
H 1.393800 1.872000 4.223600
C 2.052200 2.193700 -2.231500
H 2.539300 3.149900 -2.466900
H 1.657200 2.297100 -1.211900
C 1.245100 -0.576600 -2.993800
H 0.750600 -1.493400 -2.645000
C 2.428900 -0.316200 -2.027500
H 2.014700 -0.291500 -1.010100
C 3.083600 1.057300 -2.266100
H 3.598400 1.072600 -3.236500
H 3.860000 1.224900 -1.506900
C 2.052200 2.193700 2.231500
H 1.657200 2.297100 1.211900
H 2.539300 3.149900 2.466900
C 1.245100 -0.576600 2.993800
H 0.750600 -1.493400 2.645000
C 2.428900 -0.316200 2.027500
H 2.014700 -0.291500 1.010100
C 3.083600 1.057300 2.266100
H 3.860000 1.224900 1.506900
H 3.598400 1.072600 3.236500
C 1.739400 -0.904100 -4.422100
H 2.223900 -0.030600 -4.877600
H 0.873800 -1.138300 -5.057100
C 3.442700 -1.479500 -2.075200
H 2.968800 -2.375400 -1.647400
H 4.302000 -1.248700 -1.430600
C 3.919100 -1.813600 -3.497300
H 4.517100 -0.983600 -3.899000
H 4.584900 -2.685900 -3.471000
C 2.728300 -2.079900 -4.429000
H 2.208300 -2.991700 -4.098100
H 3.077700 -2.274900 -5.451200
C 3.442700 -1.479500 2.075200
H 4.302000 -1.248700 1.430600
H 2.968800 -2.375400 1.647400
C 1.739400 -0.904100 4.422100
H 2.223900 -0.030600 4.877600
H 0.873800 -1.138300 5.057100
C 2.728300 -2.079900 4.429000
H 3.077700 -2.274900 5.451200
H 2.208300 -2.991700 4.098100
C 3.919100 -1.813600 3.497300
H 4.517100 -0.983600 3.899000
H 4.584900 -2.685900 3.471000

Pseudorotation TS for [g^+*aa*g^-]

DFT energy	-1640.25687558
gCP correction	0.3011753377
D3 correction	-0.27986198
gCP-D3 correction	0.0213133577
DFT-gCP-D3 energy	-1640.2355622223

H 3.909200 -0.362800 0.866500
C 3.310700 0.046400 1.681400
C 3.639000 1.566900 1.761700
H 4.546500 1.757600 1.175700
C 2.512800 2.493400 1.282900
H 2.914300 3.503100 1.123700
C 1.822800 -0.252900 1.316500
C 1.386600 0.508000 0.000000
H 0.296300 0.473300 0.000000
C 1.774300 2.043900 0.000000
H 0.833200 2.609800 0.000000
C 1.822800 -0.252900 -1.316500
H 1.779900 -1.321800 -1.067600
C 2.512800 2.493400 -1.282900
H 1.777600 2.602300 -2.087800
H 2.914300 3.503100 -1.123700
C 3.639000 1.566900 -1.761700
H 4.546500 1.757600 -1.175700
C 3.310700 0.046400 -1.681400
H 3.909200 -0.362800 -0.866500
H 1.779900 -1.321800 1.067600
H 3.895800 1.844200 -2.792300
C 0.886400 -0.041000 -2.569000
H 0.955400 1.017900 -2.827200
C 3.784500 -0.704100 -2.939800
H 3.830000 -1.780200 -2.724400
H 4.813500 -0.398900 -3.174900
C 2.869100 -0.460700 -4.137900
H 2.920900 0.597000 -4.433700
H 3.212400 -1.038600 -5.007000
C 1.411100 -0.817900 -3.815800
H 0.808000 -0.487700 -4.673600
H 3.895800 1.844200 2.792300
H 1.777600 2.602300 2.087800
C 0.886400 -0.041000 2.569000
H 0.955400 1.017900 2.827200

C 3.784500 -0.704100 2.939800
H 4.813500 -0.398900 3.174900
H 3.830000 -1.780200 2.724400
C 2.869100 -0.460700 4.137900
H 2.920900 0.597000 4.433700
H 3.212400 -1.038600 5.007000
C 1.411100 -0.817900 3.815800
H 0.808000 -0.487700 4.673600
C -0.631300 -0.357300 2.365200
H -0.942200 0.069300 1.404100
C 1.186000 -2.335800 3.687400
H 1.550600 -2.835600 4.595600
H 1.775400 -2.745700 2.857100
C -0.294300 -2.662600 3.465500
H -0.424000 -3.740400 3.295800
H -0.852100 -2.431600 4.382500
C -0.882800 -1.891400 2.267400
H -0.349500 -2.249200 1.373300
C -0.631300 -0.357300 -2.365200
H -0.942200 0.069300 -1.404100
C 1.186000 -2.335800 -3.687400
H 1.775400 -2.745700 -2.857100
H 1.550600 -2.835600 -4.595600
C -0.294300 -2.662600 -3.465500
H -0.424000 -3.740400 -3.295800
H -0.852100 -2.431600 -4.382500
C -0.882800 -1.891400 -2.267400
H -0.349500 -2.249200 -1.373300
C -2.376900 -2.205200 -2.061000
H -2.685000 -1.847200 -1.070800
H -2.526200 -3.293600 -2.049100
C -1.544500 0.292300 -3.459600
H -1.265300 -0.144600 -4.427300
C -3.049300 -0.047900 -3.248300
H -3.574500 0.293000 -4.154800
C -3.272300 -1.565500 -3.129900
H -4.328000 -1.770100 -2.904600
H -3.073400 -2.032000 -4.104000
C -1.544500 0.292300 3.459600
H -1.265300 -0.144600 4.427300
C -2.376900 -2.205200 2.061000
C -3.272300 -1.565500 3.129900
C -3.049300 -0.047900 3.248300
H -3.574500 0.293000 4.154800
H -3.073400 -2.032000 4.104000
H -2.526200 -3.293600 2.049100
H -4.328000 -1.770100 2.904600
H -2.685000 -1.847200 1.070800
C -1.406500 1.826500 3.612600
H -0.363500 2.120400 3.774100
H -1.939900 2.115000 4.530700
C -3.665800 0.737700 2.072400
H -3.212500 0.424300 1.123500
H -4.733800 0.491700 1.995900
C -3.484300 2.253800 2.233500
H -3.892600 2.780900 1.361300
H -4.063000 2.596300 3.104400
C -2.007700 2.618400 2.441400
H -1.445100 2.416100 1.518600
H -1.905900 3.694900 2.631100
C -1.406500 1.826500 -3.612600
H -1.939900 2.115000 -4.530700
H -0.363500 2.120400 -3.774100
C -3.665800 0.737700 -2.072400
H -3.212500 0.424300 -1.123500
H -4.733800 0.491700 -1.995900
C -3.484300 2.253800 -2.233500
H -4.063000 2.596300 -3.104400
H -3.892600 2.780900 -1.361300
C -2.007700 2.618400 -2.441400
H -1.445100 2.416100 -1.518600
H -1.905900 3.694900 -2.631100

CT to TT TS for [a*g*$^-$*g*$^+$a]

DFT energy	-1640.25465527
gCP correction	0.3013544362
D3 correction	-0.27848076
gCP-D3 correction	0.0228736762
DFT-gCP-D3 energy	-1640.2317815938

H 3.178700 -4.166700 -0.758000
C 2.172700 -4.227100 -0.325700
C 1.986400 -3.024000 0.625300
H 2.996000 -2.646800 0.829100
C 1.192000 -1.840600 0.000800
H 1.707300 -1.560200 -0.926100
C 1.161800 -4.375200 -1.490100
C -0.034400 -3.378500 -1.610300
H -0.931100 -4.003200 -1.610200
C -0.226300 -2.329500 -0.458200
H -0.676200 -2.863600 0.382700
C -0.018100 -2.660200 -2.971900
H 0.720900 -1.846100 -2.943000
C -1.255100 -1.206800 -0.918300
H -0.646300 -0.388700 -1.326100
C -2.199900 -1.669400 -2.065400
H -2.779700 -0.785300 -2.357700
C -1.410600 -2.114700 -3.320800
H -1.316000 -1.275200 -4.021500
H 1.725300 -4.327300 -2.428700
H -1.983300 -2.888400 -3.850300
H 0.318700 -3.345100 -3.759500

H 0.747800 -5.389900 -1.465200
H 2.174700 -5.145600 0.274100
C -3.240000 -2.716700 -1.599600
H -2.759200 -3.681200 -1.401500
H -3.940600 -2.902200 -2.425600
C -2.154900 -0.628700 0.228300
H -1.499000 -0.337800 1.054500
C -3.101600 -1.741900 0.764000
H -2.478600 -2.582800 1.098700
C -4.021900 -2.283400 -0.348800
H -4.599600 -3.132700 0.041300
C 1.290600 -0.611100 0.967600
C 0.785900 -0.986900 2.394400
H -0.275400 -1.254300 2.302900
C 1.423600 -3.421700 2.003000
H 2.013400 -4.257400 2.403000
H 0.392800 -3.793200 1.916800
C 1.480600 -2.236200 2.969800
H 1.008400 -2.495800 3.927300
H -4.761300 -1.526700 -0.635400
H 2.533100 -2.023000 3.198200
C -3.017100 0.627000 -0.158200
H -3.750900 0.293700 -0.901600
C -3.880300 -1.248200 1.996200
H -3.167100 -1.041100 2.806600
H -4.542500 -2.043500 2.366200
C -4.695900 0.012900 1.692800
H -5.173100 0.385600 2.609700
H -5.516400 -0.246600 1.011300
C -3.856800 1.135900 1.055200
H -4.576100 1.869400 0.660800
C 0.878000 0.212500 3.354000
H 0.527000 -0.084000 4.352100
H 0.624500 0.172400 0.585400
H 0.194800 1.002000 3.012300
C 2.739900 0.002200 1.028700
H 3.402800 -0.792500 1.394900
C 2.302300 0.770800 3.442800
H 2.322400 1.645300 4.107500
H 2.958900 0.024200 3.908100
C 2.871400 1.159700 2.066200
H 3.949100 1.329500 2.206800
C -2.264100 1.813200 -0.825200
H -1.532400 1.405800 -1.537400
C -2.989800 1.901100 2.070000
H -2.234300 1.239100 2.513200
H -3.620400 2.245700 2.901000
C -1.460900 2.668800 0.187300
H -0.661100 2.026500 0.582100
C -2.302300 3.098500 1.402700
H -1.654700 3.618100 2.122400
H -3.069400 3.825300 1.103100
C 3.320400 0.446700 -0.347800
C 2.293400 2.493100 1.558200
H 2.473100 3.270100 2.313900
H 1.205300 2.423700 1.443500
C 2.745000 1.801500 -0.836600
H 1.661500 1.668100 -0.979800
C 2.916600 2.906200 0.219800
H 2.458600 3.835600 -0.143800
C -3.224100 2.678200 -1.675200
H -4.036700 3.074600 -1.052600
H -3.703500 2.040100 -2.430000
C -0.784100 3.864900 -0.515800
H -0.269700 4.486600 0.229700
H -0.003900 3.485100 -1.191600
C -2.505800 3.848300 -2.361500
H -3.226500 4.452400 -2.928100
H -1.785600 3.454500 -3.094600
C -1.759900 4.717300 -1.340800
H -2.487200 5.205300 -0.677600
H -1.214500 5.523300 -1.848500
C 3.340200 2.182400 -2.208700
H 2.963800 3.167900 -2.516300
H 3.980600 3.128300 0.374800
C 4.876100 2.171400 -2.226000
H 5.266300 2.973100 -1.583600
H 5.237900 2.387600 -3.239700
C 4.865900 0.452600 -0.358700
H 5.229700 -0.540900 -0.062500
H 5.262600 1.156300 0.385000
C 5.422000 0.820900 -1.743100
H 6.519500 0.837100 -1.719200
H 5.137700 0.039600 -2.464400
H 2.978400 1.464800 -2.960000
H 3.035800 -0.299400 -1.100300

Perhydro[12]helicene

Ground state

DFT energy	-1952.37927293
gCP correction	0.3573230678
D3 correction	-0.33458431
gCP-D3 correction	0.0227387578
DFT-gCP-D3 energy	-1952.3565341722

H 1.454400 1.312400 0.031700
C 1.749300 0.807800 -0.893800
C 1.366900 -1.353400 -2.212900
C 1.238400 0.886600 -3.412900
C 0.718900 -0.554800 -3.360600
C 1.076000 1.598300 -2.056100

C 1.189200 -0.651000 -0.833200
H 2.445900 -1.377900 -2.424500
H -0.373200 -0.539200 -3.250400
H -0.003600 1.620900 -1.846800
H 0.701100 1.454200 -4.185200
H 0.920600 -1.064500 -4.312800
H 1.804900 -1.211300 -0.122200
C -0.294400 -0.767600 -0.328600
H -0.917600 -0.269300 -1.078400
C 0.887200 -2.815600 -2.172300
H 1.559300 -3.394500 -1.524000
H 0.971800 -3.261200 -3.172900
C -0.753900 -2.257100 -0.297300
H -1.835800 -2.257400 -0.097900
C -0.552100 -2.942900 -1.660100
H -1.241700 -2.497000 -2.389900
H -0.832900 -4.002600 -1.585400
C 3.314000 0.911900 -0.979500
H 3.607500 0.473800 -1.939100
C 3.081100 3.176900 -2.152700
H 3.380000 4.233600 -2.115200
C 3.771200 2.401700 -1.015900
H 4.848400 2.403400 -1.237600
H 2.292100 0.877200 -3.721000
H 3.442600 2.793000 -3.116400
C 1.553500 3.061200 -2.097600
H 1.177100 3.585500 -1.208700
H 1.106500 3.570800 -2.962200
C 4.087200 0.144600 0.138600
H 3.641000 -0.852000 0.230000
C 3.605900 3.090400 0.353500
H 2.542800 3.189800 0.608200
H 3.997800 4.115000 0.290200
C 4.322400 2.324200 1.473600
H 5.407200 2.411500 1.330200
H 4.107000 2.790000 2.445200
C 3.901400 0.841900 1.517000
H 2.825700 0.830500 1.746000
C -0.577300 -0.085500 1.048700
H -0.095400 0.897400 1.035300
C -0.099800 -3.040600 0.857500
H 0.981100 -3.138100 0.692500
H -0.498000 -4.064500 0.869700
C -0.340900 -2.366200 2.213100
H 0.208100 -2.898700 3.001900
H -1.403900 -2.452300 2.473600
C 0.098400 -0.888600 2.200500
H 1.179800 -0.890100 1.999600
C -2.096400 0.163400 1.362400
H -2.557200 -0.824200 1.459900
C -0.100300 -0.200500 3.562800
H 0.336800 -0.820600 4.357600
H 0.462200 0.743200 3.570600
C -1.575900 0.081000 3.868600
H -2.105900 -0.869200 4.020600
H -1.665100 0.633600 4.814100
C -2.271900 0.865700 2.741700
H -3.348100 0.863300 2.969500
C 4.606900 0.057500 2.639200
H 4.503700 0.597700 3.590300
C 5.603200 -0.080400 -0.171200
H 6.087200 0.904300 -0.218200
C 6.311900 -0.865900 0.972600
H 7.391400 -0.822500 0.756400
C 6.090700 -0.195200 2.340200
H 6.634700 0.758800 2.359800
H 6.533200 -0.813300 3.133400
H 4.092600 -0.903700 2.777700
C 5.904400 -0.784900 -1.515000
H 5.428100 -0.263000 -2.352400
C 5.934200 -2.363000 0.973800
H 6.499000 -2.873500 1.765900
H 4.873200 -2.493700 1.225100
C 5.522200 -2.273100 -1.526700
H 4.432900 -2.383100 -1.431200
C 6.214100 -3.026900 -0.382400
H 7.299500 -3.033000 -0.563100
H 5.895400 -4.077300 -0.364900
C -2.879800 0.942300 0.258400
H -2.611200 0.505200 -0.709400
C -1.839500 2.344700 2.696700
H -0.755300 2.424300 2.542300
H -2.041100 2.810000 3.671500
C -2.562600 3.118200 1.586400
H -2.164700 4.140300 1.519500
H -3.622000 3.223600 1.853200
C -2.417000 2.428400 0.215900
H -1.342400 2.426000 -0.018500
C -3.120100 3.201400 -0.913900
H -2.780200 2.806400 -1.881200
H -2.812000 4.255800 -0.890400
C -4.441000 0.838800 0.368300
H -4.741200 1.313800 1.312600
C -4.647200 3.099200 -0.827900
H -5.107300 3.610500 -1.684700
H -4.999400 3.630100 0.066800
C -5.140900 1.641900 -0.770800
H -6.212200 1.679500 -0.525400
C -4.996500 -0.614700 0.415400
H -4.374400 -1.200600 1.105000
C -5.036800 0.926400 -2.132800
H -3.992800 0.886400 -2.471100
H -5.578600 1.511900 -2.888300
C -4.923500 -1.327100 -0.961400

H -3.861200 -1.409100 -1.236800
C -5.600100 -0.499600 -2.070200
H -5.461900 -1.004500 -3.036200
H -6.684700 -0.450400 -1.905200
C -5.475800 -2.764900 -0.861900
H -4.792800 -3.359700 -0.237200
H -5.470400 -3.232800 -1.855900
C -6.421500 -0.681600 1.011000
H -7.127400 -0.098100 0.405700
H -6.416300 -0.215500 2.006000
C -6.928900 -2.128300 1.113100
H -6.300500 -2.681300 1.827600
H -7.948700 -2.144000 1.519100
C -6.883300 -2.835700 -0.249100
H -7.612200 -2.368500 -0.926000
H -7.191000 -3.884200 -0.144300
H 5.792400 -2.718500 -2.492900
H 6.987100 -0.707000 -1.694300

Pseudorotation TS for [g^{+}*aa*g^{-}]

DFT energy	-1952.34947294
gCP correction	0.3579153516
D3 correction	-0.33637142
gCP-D3 correction	0.0215439316
DFT-gCP-D3 energy	-1952.3279290084

H -1.116000 4.179800 -1.115300
C -1.854400 3.396700 -1.297300
C -1.744500 3.012800 -2.804700
H -1.089000 3.737400 -3.303800
C -1.227700 1.594400 -3.067000
H -0.996100 1.480600 -4.134600
C -1.487100 2.231500 -0.318100
C -0.108400 1.553500 -0.705500
H -0.103000 0.604200 -0.168400
C 0.011300 1.186500 -2.240500
H 0.049600 0.090600 -2.302600
C 1.136700 2.349900 -0.152600
H 0.804800 2.791900 0.796500
C 1.332000 1.667100 -2.892900
H 2.126600 0.969300 -2.606600
H 1.243300 1.564100 -3.982500
C 1.785100 3.098400 -2.552100
H 1.299100 3.814500 -3.226200
C 1.505200 3.538900 -1.086700
H 0.631100 4.190800 -1.116400
H -1.321500 2.703600 0.659800
H 2.857400 3.175700 -2.773600
C 2.431400 1.503100 0.161300
H 2.813500 1.166300 -0.803900
C 2.640000 4.407600 -0.512200
H 2.271900 4.927800 0.382800
H 2.899200 5.193600 -1.235100
C 3.881200 3.589300 -0.153600
H 4.344300 3.203100 -1.072600
H 4.636400 4.231300 0.320500
C 3.551200 2.403400 0.766200
H 4.461000 1.788600 0.820000
H -2.722100 3.119700 -3.292700
H -2.035600 0.880200 -2.870600
C -2.689200 1.223700 -0.134500
H -2.824800 0.736900 -1.102300
C -3.212900 4.052100 -0.987100
H -3.431600 4.801900 -1.759800
H -3.146300 4.605100 -0.041000
C -4.339600 3.025100 -0.901500
H -4.471500 2.539400 -1.879100
H -5.293900 3.518200 -0.670700
C -4.034200 1.956600 0.156800
H -4.828500 1.199300 0.086200
C -2.474000 0.089800 0.925300
H -1.452800 -0.292000 0.810800
C -4.099300 2.520200 1.588400
H -5.074600 3.002100 1.744100
H -3.345400 3.305100 1.732000
C -3.879700 1.420300 2.628000
H -3.856700 1.849900 3.639000
H -4.734800 0.732300 2.609200
C -2.566400 0.655900 2.375700
H -1.752900 1.389000 2.486600
C 2.244400 0.232600 1.050100
H 1.360000 -0.305100 0.688000
C 3.231600 2.822000 2.212300
H 2.343200 3.466200 2.244000
H 4.059400 3.425900 2.609200
C 3.005500 1.593700 3.102800
H 2.694700 1.906000 4.109300
H 3.961300 1.068500 3.230200
C 1.943900 0.636800 2.524200
H 0.993700 1.191900 2.515400
C 1.741900 -0.600700 3.418500
H 0.824100 -1.118900 3.112900
H 1.580300 -0.284000 4.458100
C 3.445300 -0.777400 0.964800
H 4.337700 -0.259100 1.341400
C 3.232800 -2.007600 1.898800
H 4.186500 -2.554200 1.930900
C 2.919300 -1.578100 3.342600
H 2.709500 -2.466400 3.954100
H 3.813300 -1.113800 3.780000
C -3.435100 -1.140200 0.735100
H -4.462800 -0.774300 0.867200

C -2.336000 -0.440500 3.430600
C -3.283600 -1.631700 3.250400
C -3.217100 -2.225300 1.832500
H -4.051600 -2.936500 1.746900
H -4.314700 -1.316900 3.459300
H -2.456000 -0.013500 4.435800
H -3.050100 -2.412500 3.987300
H -1.297200 -0.787100 3.371000
C -3.387800 -1.798800 -0.676000
H -3.373900 -1.002600 -1.431600
C -1.931200 -3.040600 1.597200
H -1.046200 -2.400800 1.707600
H -1.846400 -3.812300 2.374600
C -1.910300 -3.689600 0.207700
H -0.959200 -4.216800 0.050000
H -2.695600 -4.455500 0.156000
C -2.106500 -2.643800 -0.904200
H -1.244500 -1.960900 -0.850200
C 3.796700 -1.258400 -0.474700
H 3.777600 -0.389200 -1.145500
C 2.190900 -2.991100 1.332600
H 1.196700 -2.527500 1.308200
H 2.106700 -3.854700 2.006800
C 2.559300 -3.461800 -0.079900
H 3.469200 -4.074800 -0.027900
H 1.772600 -4.117600 -0.478100
C 2.766400 -2.273100 -1.036600
H 1.797100 -1.756200 -1.112100
C -4.663600 -2.616200 -0.983100
H -5.542900 -1.968600 -0.862500
H -4.787500 -3.435400 -0.262900
C -2.105700 -3.260700 -2.319300
H -2.008200 -2.448200 -3.054700
H -1.219700 -3.898300 -2.444400
C -3.382200 -4.055400 -2.636400
H -3.430800 -4.954600 -2.006400
H -3.350800 -4.410600 -3.674600
C -4.637000 -3.201700 -2.403700
H -5.542500 -3.795100 -2.585400
H -4.653000 -2.378000 -3.133400
C 3.155300 -2.713600 -2.464100
H 3.050300 -1.850800 -3.138500
H 2.446700 -3.473200 -2.822000
C 5.240100 -1.804200 -0.574000
H 5.939400 -1.033800 -0.221300
H 5.379200 -2.668100 0.089000
C 4.595600 -3.239700 -2.565200
H 4.832500 -3.477200 -3.610400
H 4.691000 -4.181300 -2.007100
C 5.596900 -2.216500 -2.010700
H 5.594100 -1.324100 -2.654600
H 6.616700 -2.621400 -2.045600

Pseudorotation TS for [ag^-g^+a]

DFT energy -1952.34655486
gCP correction 0.3599562081
D3 correction -0.33948675
gCP-D3 correction 0.0204694581
DFT-gCP-D3 energy -1952.3260854019

H 1.999500 -3.419700 -3.403000
C 2.420000 -2.943500 -2.508100
C 2.246500 -1.406100 -2.578600
H 1.658400 -1.193800 -3.477300
C 1.434500 -0.796000 -1.383500
H 0.445400 -1.268900 -1.379500
C 1.788900 -3.513300 -1.247600
C 2.320400 -2.793100 0.000000
H 3.409700 -2.954700 0.000000
C 2.102600 -1.197900 0.000000
H 3.105100 -0.758900 0.000000
C 1.788900 -3.513300 1.247600
H 0.693300 -3.442400 1.293000
C 1.434500 -0.796000 1.383500
H 0.445400 -1.268900 1.379500
C 2.246500 -1.406100 2.578600
H 1.658400 -1.193800 3.477300
C 2.420000 -2.943500 2.508100
H 1.999500 -3.419700 3.403000
H 0.693300 -3.442400 -1.293000
H 3.489800 -3.196800 2.504900
H 2.016800 -4.583700 1.160500
H 2.016800 -4.583700 -1.160500
H 3.489800 -3.196800 -2.504900
C 3.601600 -0.696700 2.768500
H 4.242500 -0.886100 1.895400
H 4.121900 -1.147600 3.624700
C 1.206900 0.716200 1.730900
H 0.694500 1.183400 0.888600
C 2.565000 1.458900 1.902300
H 3.113800 1.349600 0.959200
C 3.450600 0.813400 2.985600
H 4.437100 1.297300 2.979600
C 1.206900 0.716200 -1.730900
C 2.565000 1.458900 -1.902300
H 3.113800 1.349600 -0.959200
C 3.601600 -0.696700 -2.768500
H 4.121900 -1.147600 -3.624700
H 4.242500 -0.886100 -1.895400
C 3.450600 0.813400 -2.985600
H 4.437100 1.297300 -2.979600
H 3.035400 0.991900 3.985700

H 3.035400 0.991900 -3.985700
C 0.308600 0.973300 3.005100
H 0.886000 0.635800 3.869100
C 2.351800 2.967100 2.126600
H 1.933200 3.406100 1.210600
H 3.321300 3.459700 2.283900
C 1.418700 3.261800 3.307300
H 1.225400 4.341400 3.372700
H 1.920900 2.987400 4.244900
C 0.083500 2.499200 3.220700
H -0.405600 2.613800 4.199000
C 2.351800 2.967100 -2.126600
H 3.321300 3.459700 -2.283900
H 0.694500 1.183400 -0.888600
H 1.933200 3.406100 -1.210600
C 0.308600 0.973300 -3.005100
H 0.886000 0.635800 -3.869100
C 1.418700 3.261800 -3.307300
H 1.225400 4.341400 -3.372700
H 1.920900 2.987400 -4.244900
C 0.083500 2.499200 -3.220700
H -0.405600 2.613800 -4.199000
C -0.887600 3.090500 2.183000
H -1.056900 4.152800 2.406300
H -0.445300 3.059300 1.178800
C -1.056600 0.219200 3.047200
H -0.875300 -0.821000 2.751900
C -2.038700 0.816100 2.001400
H -1.578000 0.668800 1.015200
C -2.225100 2.337100 2.174700
H -2.760000 2.552200 3.108800
H -2.865600 2.714700 1.365700
C -0.887600 3.090500 -2.183000
H -0.445300 3.059300 -1.178800
H -1.056900 4.152800 -2.406300
C -1.056600 0.219200 -3.047200
H -0.875300 -0.821000 -2.751900
C -2.038700 0.816100 -2.001400
H -1.578000 0.668800 -1.015200
C -2.225100 2.337100 -2.174700
H -2.865600 2.714700 -1.365700
H -2.760000 2.552200 -3.108800
C -1.713300 0.169800 4.466800
H -1.932700 1.202300 4.769700
C -3.379300 0.058600 1.992700
H -3.210200 -0.961200 1.620400
H -4.068100 0.531700 1.279700
C -4.027800 -0.003500 3.381800
H -4.335500 1.005700 3.687300
H -4.948400 -0.601800 3.340500
C -3.079700 -0.580100 4.447900
H -3.543900 -0.402400 5.431200
C -3.379300 0.058600 -1.992700
H -4.068100 0.531700 -1.279700
H -3.210200 -0.961200 -1.620400
C -1.713300 0.169800 -4.466800
H -1.932700 1.202300 -4.769700
C -3.079700 -0.580100 -4.447900
H -3.543900 -0.402400 -5.431200
C -4.027800 -0.003500 -3.381800
H -4.335500 1.005700 -3.687300
H -4.948400 -0.601800 -3.340500
C -0.832800 -0.438500 5.584900
H -1.321300 -0.219300 6.546100
H 0.145200 0.053700 5.632600
C -2.907300 -2.108600 4.325400
H -3.897100 -2.584100 4.359300
H -2.476800 -2.371400 3.349600
C -2.013700 -2.674700 5.438100
H -2.516400 -2.540400 6.407700
H -1.876800 -3.755700 5.305000
C -0.655700 -1.960900 5.474100
H -0.055700 -2.326100 6.317800
H -0.091700 -2.210200 4.564800
C -0.832800 -0.438500 -5.584900
H -1.321300 -0.219300 -6.546100
H 0.145200 0.053700 -5.632600
C -2.907300 -2.108600 -4.325400
H -3.897100 -2.584100 -4.359300
H -2.476800 -2.371400 -3.349600
C -0.655700 -1.960900 -5.474100
H -0.091700 -2.210200 -4.564800
H -0.055700 -2.326100 -6.317800
C -2.013700 -2.674700 -5.438100
H -1.876800 -3.755700 -5.305000
H -2.516400 -2.540400 -6.407700

CT to TT TS for [a*g*$^-$*g*$^+$a]

DFT energy	-1952.34038236
gCP correction	0.3613613223
D3 correction	-0.34179583
gCP-D3 correction	0.0195654923
DFT-gCP-D3 energy	-1952.3208168677

H 3.786000 -2.700900 -2.135100
C 2.954000 -3.280300 -1.716500
C 2.598700 -2.677000 -0.344100
H 3.498400 -2.140600 -0.020700
C 1.448900 -1.628600 -0.392700
H 1.721200 -0.872600 -1.141900
C 1.827300 -3.324000 -2.771600
C 0.396200 -2.816000 -2.404600

H -0.262700 -3.678900 -2.538700
C 0.135500 -2.295000 -0.938100
H -0.055500 -3.183600 -0.329700
C -0.062700 -1.770200 -3.435700
H 0.401300 -0.801400 -3.199700
C -1.193300 -1.416900 -0.911900
H -0.877300 -0.385600 -1.121200
C -2.194100 -1.824900 -2.032500
H -3.033800 -1.125900 -1.965400
C -1.589500 -1.656600 -3.446700
H -1.890500 -0.690700 -3.872500
H 2.163900 -2.743500 -3.637600
H -2.007200 -2.425200 -4.111300
H 0.296900 -2.041800 -4.435700
H 1.728200 -4.349800 -3.144700
H 3.358400 -4.288700 -1.562000
C -2.804200 -3.225400 -1.785600
H -2.043000 -4.007300 -1.892200
H -3.549600 -3.427600 -2.567300
C -1.995500 -1.419600 0.439400
H -1.306500 -1.172900 1.250800
C -2.535400 -2.851500 0.727100
H -1.675300 -3.534300 0.748000
C -3.460400 -3.349500 -0.402000
H -3.732800 -4.396800 -0.211500
C 1.389600 -0.910300 0.997500
C 1.167100 -1.955900 2.134500
H 0.202100 -2.443600 1.943900
C 2.352400 -3.738100 0.744900
H 3.190500 -4.448500 0.751400
H 1.455700 -4.333500 0.523200
C 2.220400 -3.081000 2.122700
H 1.951300 -3.829000 2.881100
H -4.403700 -2.791000 -0.401200
H 3.201600 -2.689000 2.418900
C -3.201900 -0.404300 0.520300
H -3.949700 -0.763300 -0.189700
C -3.191600 -2.922800 2.116600
H -2.420800 -2.745300 2.879500
H -3.573500 -3.937100 2.298300
C -4.322600 -1.901500 2.280700
H -4.702100 -1.921000 3.311600
H -5.167800 -2.191200 1.642000
C -3.893700 -0.468100 1.915200
H -4.817900 0.122300 1.832300
C 1.072400 -1.279000 3.513000
H 0.945700 -2.043600 4.291700
H 0.515500 -0.248400 0.988900
H 0.169400 -0.654700 3.550300
C 2.651700 -0.020000 1.310400
H 3.507000 -0.700900 1.349700
C 2.302800 -0.419000 3.820400
H 2.178600 0.084900 4.788900
H 3.182100 -1.067800 3.927300
C 2.579500 0.628300 2.725200
H 3.576600 1.046400 2.930600
C -3.057300 0.214500 3.009800
H -3.600600 0.170800 3.963800
H -2.112600 -0.321200 3.172200
C -2.893400 1.076200 0.133000
H -2.246800 1.059300 -0.753400
C -2.087100 1.784400 1.257000
H -1.130900 1.250700 1.341800
C -2.763000 1.672000 2.638300
H -3.700900 2.242200 2.654500
H -2.112100 2.134700 3.393300
C 1.595000 1.807600 2.786800
H 0.565400 1.449500 2.668800
H 1.644900 2.266400 3.784200
C 2.973900 1.065600 0.232700
H 2.856200 0.604400 -0.753700
C 1.946400 2.227200 0.304900
H 0.953000 1.795000 0.116400
C 1.894600 2.856600 1.709600
H 1.133200 3.647200 1.726200
H 2.846700 3.349000 1.946100
C -4.167100 1.900600 -0.255700
H -4.826400 1.925900 0.623000
C -1.766400 3.244700 0.891300
H -1.070400 3.257200 0.041600
H -1.239600 3.724300 1.726900
C -3.019300 4.050900 0.532900
H -3.655200 4.161900 1.421900
H -2.737000 5.069300 0.232300
C -3.834300 3.386400 -0.588100
H -4.800600 3.912200 -0.650200
C 2.198800 3.268700 -0.801000
H 1.486000 4.098800 -0.698600
H 1.992400 2.809700 -1.778200
C 4.436100 1.615100 0.286500
H 4.566300 2.131300 1.246900
C 4.683500 2.682100 -0.825300
H 5.663300 3.136700 -0.607600
C 3.635100 3.806400 -0.779500
H 3.789500 4.399900 0.131600
H 3.793100 4.494200 -1.621500
C -5.009900 1.317600 -1.417100
H -5.973500 1.847900 -1.423200
H -5.254500 0.264200 -1.241300
C -3.173000 3.549100 -1.973000
H -2.993700 4.616900 -2.159600
H -2.186400 3.066400 -1.992100
C -4.043000 2.956200 -3.088700
H -4.983500 3.523600 -3.153600

H -3.545200 3.061100 -4.061400
C -4.366600 1.484300 -2.803400
H -5.040200 1.084600 -3.572700
H -3.443100 0.893300 -2.867000
C 5.554300 0.550800   0.203700
H 6.503700 1.047300   0.454200
H 5.419500 -0.226000   0.965200
C 4.808600 2.042900 -2.225100
H 5.015300 2.831100 -2.961600
H 3.857200 1.584400 -2.527400
C 5.696400 -0.088200 -1.186200
H 4.795200 -0.670400 -1.424100
H 6.530200 -0.802700 -1.182600
C 5.912300 0.978200 -2.269400
H 5.954500 0.512900 -3.262800
H 6.887600 1.461900 -2.109400

Perhydro[20]helicene

Ground state

DFT energy	-3200.73959284
gCP correction	0.5874015720
D3 correction	-0.56692514
gCP-D3 correction	0.0204764319999999
DFT-gCP-D3 energy	-3200.719116408

H -5.638000 0.288600 -1.245500
C -6.092900 0.954700 -0.505500
C -5.950800 1.611000 1.964900
C -6.056300 3.430700 0.188200
C -5.533300 3.042600 1.576600
C -5.650700 2.399800 -0.883600
C -5.521200 0.562200 0.895100
H -7.050000 1.607500 1.996000
H -4.440000 3.143500 1.590000
H -4.551100 2.403400 -0.913900
H -5.669900 4.418700 -0.097800
H -5.911200 3.745100 2.332200
H -5.986600 -0.384300 1.187500
C -3.967700 0.327800 0.932900
H -3.504100 1.295300 0.718200
C -5.463600 1.217700 3.370900
H -6.000900 0.316200 3.696000
H -5.731100 2.005400 4.088600
C -3.502000 -0.072600 2.365200
H -2.402200 -0.062700 2.360900
C -3.953300 0.957800 3.415900
H -3.414100 1.899900 3.246300
H -3.660200 0.616300 4.418100
C -7.647600 0.788100 -0.654500
H -8.108900 1.505000 0.032500
C -7.649200 2.605900 -2.460200
H -7.942900 2.831000 -3.494700
C -8.115100 1.187000 -2.086300
H -9.214800 1.207000 -2.072500
H -7.148400 3.533600 0.230900
H -8.178700 3.332200 -1.828700
C -6.136500 2.790200 -2.291200
H -5.607600 2.176900 -3.033300
H -5.858000 3.831700 -2.502800
C -8.195900 -0.627100 -0.285400
H -7.722700 -0.932900 0.654200
C -7.713500 0.136100 -3.140700
H -6.622500 0.105500 -3.258700
H -8.115300 0.435500 -4.118500
C -8.218700 -1.264900 -2.772100
H -9.313200 -1.284800 -2.856000
H -7.845800 -2.002900 -3.495700
C -7.777900 -1.678100 -1.354700
H -6.678600 -1.710800 -1.367700
C -3.429600 -0.697200 -0.116900
H -3.909600 -0.472700 -1.074200
C -3.919400 -1.510300 2.730700
H -5.011500 -1.584700 2.816300
H -3.519400 -1.760600 3.722900
C -3.426900 -2.525200 1.691900
H -3.809300 -3.527000 1.931200
H -2.332700 -2.596600 1.749500
C -3.869600 -2.142400 0.266200
H -4.969500 -2.153700 0.271400
C -1.880200 -0.633700 -0.371800
H -1.397900 -0.935100 0.563200
C -3.418200 -3.169900 -0.787100
H -3.692500 -4.181600 -0.457800
H -3.972600 -2.993200 -1.719000
C -1.912500 -3.099500 -1.066800
H -1.358000 -3.431200 -0.178300
H -1.646000 -3.800400 -1.869800
C -1.450800 -1.680600 -1.443600
H -0.351200 -1.696600 -1.455700
C -8.263300 -3.088600 -0.972300
H -8.002000 -3.798300 -1.769200
C -9.738100 -0.668400 -0.033500
H -10.240700 -0.408100 -0.974500
C -10.220100 -2.101600 0.343300
H -11.321300 -2.073300 0.329200
C -9.772900 -3.139900 -0.701100
H -10.318200 -2.960100 -1.637600
H -10.060600 -4.147500 -0.370700
H -7.718500 -3.422100 -0.078200
C -10.258500 0.323900 1.033700
H -9.946100 1.349900 0.808400

C -9.818700 -2.492400 1.781500
H -10.222800 -3.488700 2.007200
H -8.727300 -2.580500 1.866600
C -9.859800 -0.051500 2.469300
H -8.769500 0.017500 2.590000
C -10.322900 -1.474000 2.814700
H -11.422900 -1.496600 2.834500
H -9.987300 -1.754400 3.821500
C -1.332500 0.780800 -0.749000
H -1.788700 1.503800 -0.065500
C -1.893500 -1.275000 -2.862800
H -2.987800 -1.300600 -2.948400
H -1.516200 -2.014100 -3.582800
C -1.393500 0.126200 -3.235400
H -1.796800 0.422900 -4.213400
H -0.302500 0.099000 -3.353700
C -1.798900 1.176000 -2.182100
H -2.898800 1.191300 -2.170200
C -1.337600 2.596100 -2.556300
H -1.868700 3.321100 -1.924500
H -1.632600 2.820400 -3.590500
C 0.223200 0.948300 -0.602200
H 0.677700 0.283000 -1.342900
C 0.174700 2.784000 -2.388400
H 0.450600 3.826200 -2.599800
H 0.704200 2.172400 -3.131500
C 0.663200 2.393900 -0.981600
H 1.762700 2.397900 -1.014300
C 0.799000 0.556900 0.797000
H 0.335300 -0.389700 1.091400
C 0.260000 3.425100 0.090600
H -0.831900 3.528200 0.136200
H 0.645400 4.413000 -0.196900
C 0.372000 1.606000 1.867700
H -0.727400 1.601600 1.902200
C 0.787200 3.037700 1.477700
H 0.411200 3.740200 2.234300
H 1.880400 3.139100 1.487800
C 0.863500 1.213600 3.272400
H 0.327600 0.312100 3.599500
H 0.597700 2.001500 3.990500
C 2.352800 0.323300 0.830500
H 2.815600 1.290800 0.614100
C 2.822500 -0.076300 2.261700
H 3.922300 -0.066200 2.254200
C 2.374100 0.954600 3.313000
H 2.912400 1.896800 3.141200
H 2.670400 0.613900 4.314600
H -10.292200 0.669800 3.174800
H -11.357000 0.328200 0.973000
C 2.888400 -0.702100 -0.220300
H 2.405900 -0.478400 -1.176500
C 2.406500 -1.513800 2.629100
H 1.314600 -1.588600 2.717600
H 2.809100 -1.763400 3.620400
C 2.896800 -2.529200 1.589700
H 2.515300 -3.531000 1.830500
H 3.991100 -2.600000 1.644700
C 2.450400 -2.147400 0.164900
H 1.350600 -2.159600 0.172600
C 4.437100 -0.637600 -0.479000
H 4.921800 -0.937000 0.455400
C 2.900300 -3.175500 -0.888600
H 2.627100 -4.187000 -0.558000
H 2.344000 -2.999800 -1.819600
C 4.405400 -3.104500 -1.171100
H 4.670900 -3.806000 -1.973900
H 4.961600 -3.435100 -0.283200
C 4.865500 -1.685600 -1.550200
H 5.965200 -1.700800 -1.564100
C 4.419200 -1.282200 -2.968900
H 3.324700 -1.309800 -3.051900
H 4.795900 -2.021700 -3.688900
C 4.983100 0.776500 -0.859800
H 4.529400 1.500600 -0.175700
C 4.915800 0.119300 -3.345100
H 4.508800 0.413900 -4.322200
H 6.006400 0.093400 -3.467000
C 4.512700 1.170200 -2.292000
H 3.412900 1.185900 -2.277500
C 4.974000 2.589800 -2.668500
H 4.675300 2.814000 -3.701700
H 4.446000 3.315500 -2.034900
C 6.539000 0.942200 -0.719300
H 6.989600 0.275700 -1.460900
C 6.487300 2.776100 -2.506800
H 7.012700 2.163700 -3.252100
H 6.763300 3.818000 -2.719800
C 6.982000 2.385800 -1.102100
H 8.081300 2.385600 -1.140500
C 6.585300 3.418200 -0.028400
H 5.493800 3.525000 0.019400
H 6.973500 4.404900 -0.316400
C 7.120200 0.551900 0.677800
H 6.662800 -0.398600 0.973500
C 7.114400 3.029500 1.357900
H 6.742700 3.733900 2.114900
H 8.207800 3.126900 1.364400
C 6.697000 1.598900 1.750200
H 5.597900 1.595800 1.790200
C 7.197000 1.202800 3.150600
H 6.671200 0.293800 3.473800
H 6.927400 1.983700 3.874800
C 8.671000 0.316900 0.697500

H 9.154500 1.271100 0.446600
C 8.710200 0.958800 3.181400
H 9.016600 0.619600 4.180400
H 9.236800 1.907100 3.008200
C 9.165900 -0.065300 2.126300
H 10.264900 -0.030500 2.105300
C 8.789200 -1.512500 2.500700
H 7.699100 -1.621200 2.579000
H 9.190100 -1.741700 3.497600
C 9.189300 -0.720600 -0.341200
H 8.690700 -0.528300 -1.300300
C 9.324400 -2.520500 1.474800
H 8.999600 -3.537000 1.736100
H 10.421900 -2.529600 1.521200
C 8.848500 -2.183400 0.049100
H 7.752100 -2.278700 0.054900
C 10.697000 -0.550100 -0.636700
H 11.293900 -0.695300 0.273100
H 10.883600 0.482300 -0.962800
C 9.380500 -3.171500 -1.010900
H 9.184100 -4.202400 -0.686000
H 8.811300 -3.027400 -1.941400
C 11.181200 -1.534800 -1.712200
H 12.256900 -1.404800 -1.888300
H 10.679300 -1.304800 -2.664300
C 10.874300 -2.987000 -1.320200
H 11.477100 -3.262400 -0.443500
H 11.174400 -3.670500 -2.125100

Cs structure (presumed TS)

DFT energy	-3200.70076436
gCP correction	0.5881953505
D3 correction	-0.56668436
gCP-D3 correction	0.0215109905
DFT-gCP-D3 energy	-3200.6792533695

H 4.362800 -1.674600 3.402300
C 3.937500 -2.142500 2.505400
C 2.390300 -2.149100 2.576600
H 2.111000 -1.594900 3.478900
C 1.688000 -1.407400 1.385900
H 2.044200 -0.370600 1.387300
C 4.428400 -1.444600 1.247300
C 3.773500 -2.054200 0.000000
H 4.058600 -3.118100 0.000000
C 2.164000 -2.020700 0.000000
H 1.843900 -3.066700 0.000000
C 4.428400 -1.444600 -1.247300
H 4.230000 -0.365000 -1.295700
C 1.688000 -1.407400 -1.385900
H 2.044200 -0.370600 -1.387300
C 2.390300 -2.149100 -2.576600
H 2.111000 -1.594900 -3.478900
C 3.937500 -2.142500 -2.505400
H 4.362800 -1.674600 -3.402300
H 4.230000 -0.365000 1.295700
H 4.311100 -3.176200 -2.495600
H 5.518000 -1.546100 -1.158400
H 5.518000 -1.546100 1.158400
H 4.311100 -3.176200 2.495600
C 1.845100 -3.578800 -2.759000
H 2.103200 -4.187900 -1.880700
H 2.356300 -4.049200 -3.610200
C 0.157800 -1.359000 -1.733000
H -0.366000 -0.903300 -0.891200
C -0.416800 -2.797400 -1.901400
H -0.245600 -3.329500 -0.957800
C 0.329700 -3.601600 -2.982400
H -0.040100 -4.636200 -2.981500
C 0.157800 -1.359000 1.733000
C -0.416800 -2.797400 1.901400
H -0.245600 -3.329500 0.957800
C 1.845100 -3.578800 2.759000
H 2.356300 -4.049200 3.610200
H 2.103200 -4.187900 1.880700
C 0.329700 -3.601600 2.982400
H -0.040100 -4.636200 2.981500
H 0.111700 -3.205000 -3.982100
H 0.111700 -3.205000 3.982100
C -0.204100 -0.498200 -3.009000
H 0.212500 -1.028700 -3.868400
C -1.938500 -2.771500 -2.132000
H -2.430900 -2.399200 -1.223200
H -2.308600 -3.795400 -2.280000
C -2.334600 -1.894200 -3.324600
H -3.429000 -1.835400 -3.402100
H -1.991300 -2.366400 -4.255100
C -1.746300 -0.474400 -3.239800
H -1.914100 -0.007000 -4.221500
C -1.938500 -2.771500 2.132000
H -2.308600 -3.795400 2.280000
H -0.366000 -0.903300 0.891200
H -2.430900 -2.399200 1.223200
C -0.204100 -0.498200 3.009000
H 0.212500 -1.028700 3.868400
C -2.334600 -1.894200 3.324600
H -3.429000 -1.835400 3.402100
H -1.991300 -2.366400 4.255100
C -1.746300 -0.474400 3.239800
H -1.914100 -0.007000 4.221500
C -2.470700 0.414100 -2.213600
H -3.544000 0.438500 -2.447200

H -2.389900 -0.014400 -1.205800
C 0.373500 0.954300 -3.040200
H 1.415300 0.902300 -2.706800
C -0.372300 1.848400 -2.006800
H -0.188700 1.402500 -1.019700
C -1.900700 1.837000 -2.209700
H -2.167300 2.329600 -3.154100
H -2.371700 2.431500 -1.414600
C -2.470700 0.414100 2.213600
H -2.389900 -0.014400 1.205800
H -3.544000 0.438500 2.447200
C 0.373500 0.954300 3.040200
H 1.415300 0.902300 2.706800
C -0.372300 1.848400 2.006800
H -0.188700 1.402500 1.019700
C -1.900700 1.837000 2.209700
H -2.371700 2.431500 1.414600
H -2.167300 2.329600 3.154100
C 0.387000 1.627000 -4.460800
H -0.659900 1.728200 -4.762000
C 0.196000 3.278300 -1.969200
H 1.215300 3.249900 -1.561300
H -0.391900 3.891300 -1.272900
C 0.216300 3.932400 -3.354700
H -0.813800 4.098100 -3.698200
H 0.680900 4.926300 -3.295600
C 0.960000 3.074900 -4.393100
H 0.781600 3.537400 -5.375200
C 0.196000 3.278300 1.969200
H -0.391900 3.891300 1.272900
H 1.215300 3.249900 1.561300
C 0.387000 1.627000 4.460800
H -0.659900 1.728200 4.762000
C 0.960000 3.074900 4.393100
H 0.781600 3.537400 5.375200
C 0.216300 3.932400 3.354700
H -0.813800 4.098100 3.698200
H 0.680900 4.926300 3.295600
C 1.120600 0.811700 -5.575300
H 0.806300 -0.234400 -5.485300
C 2.485700 3.088300 -4.177900
H 2.842400 4.127500 -4.190600
H 2.738500 2.693500 -3.185200
C 3.215100 2.267500 -5.246700
H 3.126000 2.778700 -6.214100
H 4.290000 2.224700 -5.023200
C 2.660600 0.833100 -5.338700
H 2.844800 0.368100 -4.359600
C 1.120600 0.811700 5.575300
H 0.806300 -0.234400 5.485300
C 2.485700 3.088300 4.177900
H 2.842400 4.127500 4.190600
H 2.738500 2.693500 3.185200
C 2.660600 0.833100 5.338700
H 2.844800 0.368100 4.359600
C 3.215100 2.267500 5.246700
H 4.290000 2.224700 5.023200
H 3.126000 2.778700 6.214100
C 0.769000 1.250900 -7.040500
H 1.102500 2.290700 -7.162800
C 3.403200 -0.015500 -6.385700
H 3.138800 -1.072600 -6.243900
H 4.486800 0.053700 -6.217300
C 3.066800 0.409200 -7.819700
H 3.464400 1.415700 -8.006200
H 3.569500 -0.253700 -8.537300
C 1.552000 0.408600 -8.090900
H 1.401100 0.893400 -9.066900
C 0.769000 1.250900 7.040500
H 1.102500 2.290700 7.162800
C 3.403200 -0.015500 6.385700
H 4.486800 0.053700 6.217300
H 3.138800 -1.072600 6.243900
C 3.066800 0.409200 7.819700
H 3.464400 1.415700 8.006200
H 3.569500 -0.253700 8.537300
C 1.552000 0.408600 8.090900
H 1.401100 0.893400 9.066900
C 0.975800 -1.012700 -8.218800
H 1.110900 -1.571100 -7.282900
H 1.539100 -1.564000 -8.984300
C -0.750800 1.243800 -7.378600
H -1.286800 1.691300 -6.531200
C -1.339600 -0.179500 -7.572200
H -1.229600 -0.709400 -6.612100
C -0.514300 -0.996400 -8.587200
H -0.622900 -0.591400 -9.600700
H -0.886300 -2.026400 -8.624900
C -0.750800 1.243800 7.378600
H -1.286800 1.691300 6.531200
C 0.975800 -1.012700 8.218800
H 1.539100 -1.564000 8.984300
H 1.110900 -1.571100 7.282900
C -0.514300 -0.996400 8.587200
H -0.622900 -0.591400 9.600700
H -0.886300 -2.026400 8.624900
C -1.339600 -0.179500 7.572200
H -1.229600 -0.709400 6.612100
C -1.097600 2.143800 -8.582800
H -0.649400 3.136400 -8.438500
H -0.666900 1.748200 -9.511300
C -2.881900 -0.089400 -7.803400
C -2.618800 2.285500 -8.726200
H -3.001100 2.795000 -7.829400

H -2.865800 2.934900 -9.577200
C -3.348700 0.935700 -8.877100
C -1.097600 2.143800 8.582800
H -0.649400 3.136400 8.438500
H -0.666900 1.748200 9.511300
C -2.881900 -0.089400 7.803400
C -2.618800 2.285500 8.726200
H -3.001100 2.795000 7.829400
H -2.865800 2.934900 9.577200
C -3.348700 0.935700 8.877100
H -4.414000 1.123200 -8.665200
H -3.258000 0.308000 -6.847300
H -3.258000 0.308000 6.847300
H -4.414000 1.123200 8.665200
C -3.315900 0.385100 -10.316200
H -3.725600 1.145500 -10.995500
H -2.285200 0.203600 -10.646600
C -3.590900 -1.452300 -8.002800
H -4.632500 -1.337900 -7.668600
H -3.142500 -2.203800 -7.338100
C -4.128200 -0.912400 -10.433200
H -5.185000 -0.691900 -10.219100
H -4.092500 -1.296400 -11.460800
C -3.621100 -1.976700 -9.450700
H -2.622000 -2.300200 -9.764400
H -4.260700 -2.868100 -9.496900
C -3.590900 -1.452300 8.002800
H -4.632500 -1.337900 7.668600
H -3.142500 -2.203800 7.338100
C -3.315900 0.385100 10.316200
H -3.725600 1.145500 10.995500
H -2.285200 0.203600 10.646600
C -4.128200 -0.912400 10.433200
H -5.185000 -0.691900 10.219100
H -4.092500 -1.296400 11.460800
C -3.621100 -1.976700 9.450700
H -4.260700 -2.868100 9.496900
H -2.622000 -2.300200 9.764400

Propellane 6

Ground state

DFT energy	-547.967398847
gCP correction	0.1012492799
D3 correction	-0.08159913
gCP-D3 correction	0.0196501499
DFT-gCP-D3 energy	-547.9477486971

H 0.471800 1.326000 -2.439300
C 0.582400 1.332700 -1.345500
C 0.044000 2.608500 0.764700
C 0.000000 0.000000 0.790700
C -0.582400 1.332700 1.345500
C -0.000000 0.000000 -0.790700
C -0.044000 2.608500 -0.764700
H 1.096000 2.693900 1.068900
H -1.661900 1.371000 1.153900
H -1.096000 2.693900 -1.068900
H 1.661900 1.371000 -1.153900
H -0.465300 3.488100 1.179800
H -0.471800 1.326000 2.439300
H 0.465300 3.488100 -1.179800
C -1.445400 -0.162000 -1.345500
H -2.018300 0.753800 -1.153900
H -1.384300 -0.254400 -2.439300
C 1.445400 -0.162000 1.345500
H 2.018300 0.753800 1.153900
H 1.384300 -0.254400 2.439300
C 0.862900 -1.170800 -1.345500
H 0.912400 -1.071600 -2.439300
H 0.356400 -2.124800 -1.153900
C -0.862900 -1.170800 1.345500
H -0.912400 -1.071600 2.439300
H -0.356400 -2.124800 1.153900
C 2.281000 -1.266200 -0.764700
H 2.881000 -0.397800 -1.068900
H 2.788100 -2.147000 -1.179800
C 2.237000 -1.342300 0.764700
H 3.253500 -1.341100 1.179800
H 1.785000 -2.296100 1.068900
C -2.281000 -1.266200 0.764700
H -2.881000 -0.397800 1.068900
H -2.788100 -2.147000 1.179800
C -2.237000 -1.342300 -0.764700
H -1.785000 -2.296100 -1.068900
H -3.253500 -1.341100 -1.179800

Transition sate for inversion

DFT energy	-547.940498986
gCP correction	0.1007773434
D3 correction	-0.08064727
gCP-D3 correction	0.0201300734
DFT-gCP-D3 energy	-547.9203689126

H -0.710500 -1.549900 2.300600
C -1.093500 -1.018700 1.419500
C 0.076500 -0.024800 -0.788900
C -2.333000 -1.186200 -0.710100
C -1.185500 -0.604400 -1.543300
C -1.773200 -1.992300 0.459200

C 0.074400 -0.161300 0.823800
H -2.976200 -0.388400 -0.311600
H -0.834100 -1.412500 -2.197500
H -1.066700 -2.751100 0.091200
H -1.880300 -0.353000 1.797800
H -2.969400 -1.801800 -1.358800
H -1.582400 0.160800 -2.221800
H -2.570100 -2.533700 0.985300
C 1.396000 -0.827700 1.311400
H 1.349500 -1.907700 1.112900
H 1.437400 -0.728600 2.404900
C 1.293000 -0.796900 -1.381400
H 1.278600 -0.675500 -2.473200
H 1.136600 -1.869200 -1.203000
C 0.226700 1.469800 -1.179700
H 0.252400 1.545200 -2.275400
H 1.197900 1.843500 -0.834200
C -0.024300 1.244500 1.489900
H 0.935600 1.770300 1.419500
H -0.206800 1.102500 2.564000
C -1.118900 2.133600 0.887300
H -2.095700 1.656000 1.032900
H -1.166200 3.082200 1.435400
C -0.875800 2.384600 -0.623600
H -0.586800 3.428300 -0.799200
H -1.807300 2.234100 -1.182900
C 2.680000 -0.261100 0.695600
H 3.547000 -0.774500 1.128800
H 2.789100 0.792500 0.980200
C 2.677300 -0.399600 -0.846500
H 3.010300 0.535700 -1.311100
H 3.399300 -1.162300 -1.164100

S4 hydrocarbon 7

Ground state

DFT energy -664.696279710
gCP correction 0.1216374046
D3 correction -0.10302116
gCP-D3 correction 0.0186162446
DFT-gCP-D3 energy -664.6776634654

C 0.000000 0.000000 0.000000
C -0.109700 -1.304700 0.876000
H 0.737300 -1.317900 1.573200
C 0.109700 1.304700 0.876000
H -0.737300 1.317900 1.573200
C -1.304700 0.109700 -0.876000
H -1.317900 -0.737300 -1.573200
C 1.304700 -0.109700 -0.876000
H 1.317900 0.737300 -1.573200
C 1.396200 1.350500 1.726700
H 1.387000 0.538700 2.465700
H 1.411100 2.284700 2.304700
C 2.663000 1.238000 0.869000
H 3.551300 1.188100 1.512300
H 2.784300 2.140500 0.256000
C 2.601600 0.000000 -0.031800
H 3.459400 -0.007300 -0.718600
H 2.717200 -0.890000 0.598200
C 0.000000 2.601600 0.031800
H 0.890000 2.717200 -0.598200
H 0.007300 3.459400 0.718600
C -1.350500 1.396200 -1.726700
H -0.538700 1.387000 -2.465700
H -2.284700 1.411100 -2.304700
C -1.238000 2.663000 -0.869000
H -2.140500 2.784300 -0.256000
H -1.188100 3.551300 -1.512300
C -1.396200 -1.350500 1.726700
H -1.411100 -2.284700 2.304700
H -1.387000 -0.538700 2.465700
C -2.601600 0.000000 -0.031800
H -3.459400 0.007300 -0.718600
H -2.717200 0.890000 0.598200
C -2.663000 -1.238000 0.869000
H -2.784300 -2.140500 0.256000
H -3.551300 -1.188100 1.512300
C 1.350500 -1.396200 -1.726700
H 2.284700 -1.411100 -2.304700
H 0.538700 -1.387000 -2.465700
C 0.000000 -2.601600 0.031800
H -0.890000 -2.717200 -0.598200
H -0.007300 -3.459400 0.718600
C 1.238000 -2.663000 -0.869000
H 2.140500 -2.784300 -0.256000
H 1.188100 -3.551300 -1.512300

CTTT2 state (second lowest CTTT)

DFT energy -664.670884459
gCP correction 0.1212629979
D3 correction -0.10274287
gCP-D3 correction 0.0185201279
SCF-gCP-D3 energy -664.6523643311

C 0.022000 0.098000 -0.051900
C 1.405700 0.183800 -0.848200
H 1.154000 0.235300 -1.917100
C -1.152200 0.037500 -1.103000
H -0.853100 0.706200 -1.920100

C -0.167300 1.393700 0.812400
H 0.626700 1.399200 1.567400
C 0.020100 -1.143300 0.925400
H -0.742400 -0.945700 1.685800
C -1.301100 -1.365200 -1.722700
H -0.390000 -1.623400 -2.282100
H -2.116600 -1.347400 -2.458000
C -1.554000 -2.438100 -0.661200
H -1.710900 -3.416800 -1.133200
H -2.479100 -2.209400 -0.114100
C -0.367600 -2.509900 0.305300
H -0.589300 -3.202300 1.129400
H 0.483000 -2.949400 -0.227600
C -2.524100 0.581900 -0.590900
H -3.338200 -0.025200 -1.008200
H -2.675600 1.591800 -0.994200
C -1.518100 1.439000 1.588400
H -1.375300 1.078800 2.614500
H -1.813200 2.491700 1.687300
C -2.670100 0.650600 0.931600
H -3.630800 1.114600 1.188800
H -2.714600 -0.365500 1.340100
C 2.193100 1.476500 -0.556700
H 2.563000 1.484500 0.477600
H 3.085600 1.495300 -1.196300
C 0.022600 2.692100 -0.007000
H -0.000200 3.531500 0.699500
H -0.836700 2.845300 -0.670600
C 1.339700 2.719900 -0.822800
H 1.914300 3.626800 -0.598300
H 1.115800 2.760800 -1.897200
C 1.357400 -1.257300 1.688100
H 1.284700 -2.082600 2.408400
H 1.498500 -0.353700 2.292700
C 2.315100 -1.051500 -0.690100
H 3.266600 -0.854700 -1.202000
H 1.866100 -1.893900 -1.226500
C 2.589000 -1.458500 0.769900
H 2.914800 -2.505600 0.788200
H 3.431700 -0.879700 1.166700

TTTT1 state (lowest TTTT)

DFT energy	-664.666855626
gCP correction	0.1213445897
D3 correction	-0.10246320
gCP-D3 correction	0.0188813897
SCF-gCP-D3 energy	-664.6479742363

C 0.000000 0.000000 0.000000
C -0.904000 0.924800 0.919800
H -0.349900 1.082200 1.857300
C 0.904000 -0.924800 0.919800
H 0.349900 -1.082200 1.857300
C 0.904000 0.924800 -0.919800
H 0.349900 1.082200 -1.857300
C -0.904000 -0.924800 -0.919800
H -0.349900 -1.082200 -1.857300
C 1.223300 -2.328900 0.349800
H 1.850500 -2.853000 1.083100
H 1.846500 -2.225400 -0.542000
C 0.000000 -3.189400 0.000000
H 0.255300 -3.850700 -0.837800
C -1.223300 -2.328900 -0.349800
H -1.846500 -2.225400 0.542000
C 2.244400 -0.249900 1.265600
H 2.798900 -0.881800 1.971300
H 2.059600 0.691100 1.795800
C 2.244400 0.249900 -1.265600
H 2.059600 -0.691100 -1.795800
H 2.798900 0.881800 -1.971300
C 3.105300 0.000000 0.000000
H 3.767100 0.855500 0.177700
C -1.223300 2.328900 0.349800
H -1.846500 2.225400 -0.542000
H -1.850500 2.853000 1.083100
C 1.223300 2.328900 -0.349800
H 1.850500 2.853000 -1.083100
H 1.846500 2.225400 0.542000
C 0.000000 3.189400 0.000000
H -0.255300 3.850700 -0.837800
C -2.244400 -0.249900 -1.265600
H -2.798900 -0.881800 -1.971300
H -2.059600 0.691100 -1.795800
C -2.244400 0.249900 1.265600
H -2.798900 0.881800 1.971300
H -2.059600 -0.691100 1.795800
C -3.105300 0.000000 0.000000
H -3.767100 -0.855500 0.177700
H -3.767100 0.855500 -0.177700
H 0.255300 3.850700 0.837800
H 3.767100 -0.855500 -0.177700
H -0.255300 -3.850700 0.837800
H -1.850500 -2.853000 -1.083100

Transition state with D_2 symmetry. (midpoint in the overall inversion scheme for 7)

DFT energy	-664.664498259
gCP correction	0.1216126952

D3 correction -0.10236451
gCP-D3 correction 0.0192481852
DFT-gCP-D3 energy -664.6452500738

C 0.000000 0.000000 0.000000
C -0.904000 0.924800 0.919800
H -0.349900 1.082200 1.857300
C 0.904000 -0.924800 0.919800
H 0.349900 -1.082200 1.857300
C 0.904000 0.924800 -0.919800
H 0.349900 1.082200 -1.857300
C -0.904000 -0.924800 -0.919800
H -0.349900 -1.082200 -1.857300
C 1.223300 -2.328900 0.349800
H 1.850500 -2.853000 1.083100
H 1.846500 -2.225400 -0.542000
C 0.000000 -3.189400 0.000000
H 0.255300 -3.850700 -0.837800
C -1.223300 -2.328900 -0.349800
H -1.846500 -2.225400 0.542000
C 2.244400 -0.249900 1.265600
H 2.798900 -0.881800 1.971300
H 2.059600 0.691100 1.795800
C 2.244400 0.249900 -1.265600
H 2.059600 -0.691100 -1.795800
H 2.798900 0.881800 -1.971300
C 3.105300 0.000000 0.000000
H 3.767100 0.855500 0.177700
C -1.223300 2.328900 0.349800
H -1.846500 2.225400 -0.542000
H -1.850500 2.853000 1.083100
C 1.223300 2.328900 -0.349800
H 1.850500 2.853000 -1.083100
H 1.846500 2.225400 0.542000
C 0.000000 3.189400 0.000000
H -0.255300 3.850700 -0.837800
C -2.244400 -0.249900 -1.265600
H -2.798900 -0.881800 -1.971300
H -2.059600 0.691100 -1.795800
C -2.244400 0.249900 1.265600
H -2.798900 0.881800 1.971300
H -2.059600 -0.691100 1.795800
C -3.105300 0.000000 0.000000
H -3.767100 -0.855500 0.177700
H -3.767100 0.855500 -0.177700
H 0.255300 3.850700 0.837800
H 3.767100 -0.855500 -0.177700
H -0.255300 -3.850700 0.837800
H -1.850500 -2.853000 -1.083100

Rate-limiting transition state for inversion of 7

DFT energy -664.662036136
gCP correction 0.1207488408
D3 correction -0.10144060
gCP-D3 correction 0.0193082408
SCF-gCP-D3 energy -664.6427278952

C1 0.0302 0.0456 -0.0643
C2 0.4244 -1.1277 0.9468
H3 -0.1439 -0.9385 1.8658
C4 -0.4383 1.2335 0.8675
H5 0.2358 1.1998 1.7350
C6 -1.1137 -0.3543 -1.1212
H7 -0.6805 -0.1636 -2.1091
C8 1.3012 0.5113 -0.8879
H9 0.9364 0.8540 -1.8668
C10 -0.2801 2.6693 0.3060
H11 -0.4670 3.3542 1.1433
H12 -1.0621 2.8927-0.4249
C13 1.1120 2.9649-0.3142
H14 0.9875 3.2922-1.3548
C15 2.0283 1.7362-0.3009
H16 2.9233 1.9406-0.9046
H17 2.3885 1.5465 0.7161
C18 -1.8609 1.0112 1.4276
H19 -2.1312 1.8755 2.0474
H20 -1.8416 0.1572 2.1142
C21 -2.3401 0.5860-1.0554
H22 -2.0544 1.5656-1.4527
C23 -2.9426 0.7642 0.3437
H24 -3.5260 -0.1273 0.6065
C25 0.0370 -2.5655 0.5573
H26 0.6794 -2.9537-0.2458
H27 0.2158 -3.2086 1.4301
C28 -1.5564 -1.8475-1.1855
H29 -2.5880 -1.8937-1.5578
C30 -1.4163 -2.6438 0.1083
H31 -1.7203 -3.6852-0.0602
C32 2.2541 -0.6652-1.1530
H33 3.0471 -0.3532-1.8451
H34 1.6944 -1.4545-1.6720
C35 1.9124 -1.0740 1.3454
H36 2.1086 -1.8592 2.0873
H37 2.1144 -0.1286 1.8614
C38 2.8707 -1.2286 0.1444
H39 3.8135 -0.7149 0.3683
H40 3.1316 -2.2834-0.0044
H41 1.5975 3.7982 0.2083
H42 -3.1085 0.2095-1.7432
H43 -3.6612 1.5924 0.3183

H44 -0.9453 -2.3611-1.9415
H45 -2.0749 -2.2468 0.8944

Structures and relative B3LYP/6-31G* energies (kJ mol^{-1}) for conformers of hexadecahydronaphtho[1,8-de]naphthalene 7

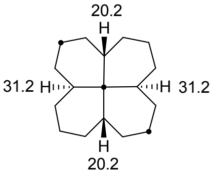

BoatC2 (75.1)

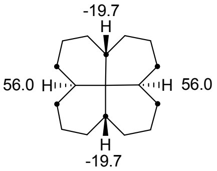

BoatC2c (79.8)

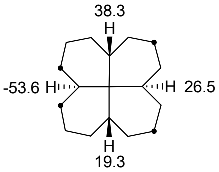

Boat4a (89.5)

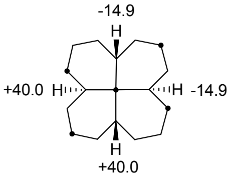

Boat322 (112.3)

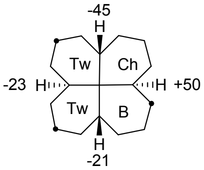

Chair1a (61.9)

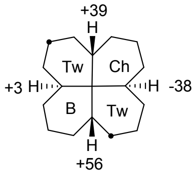

mmod30 (65.5)

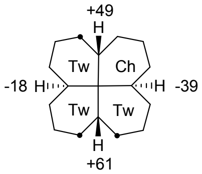

Boat4aircend (63.8)

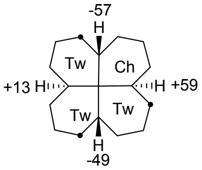

Chair1b (66.9)

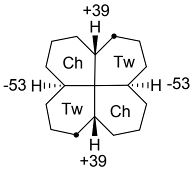

Chair2op, C_2 (52.8)

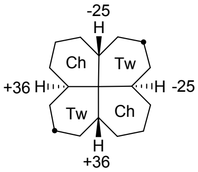

Chair2opb, C_2 (+68.5)

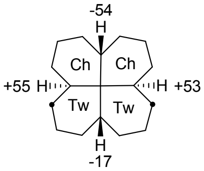

Chair2aj1 (45.2)

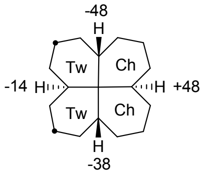

Chair2aj2 (+45.4)

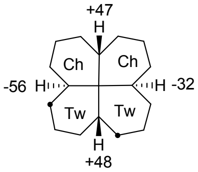

Chair2ajc (+60.9)

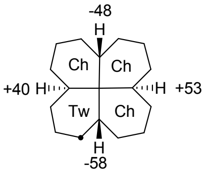

Chair3 (23.6)

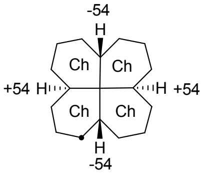

Chair4, S_4 (0.0)
